# Parasite Prevalence Corresponds to Host Life History in a Diverse Assemblage of Afrotropical Birds and Haemosporidian Parasites

**DOI:** 10.1371/journal.pone.0121254

**Published:** 2015-04-08

**Authors:** Holly L. Lutz, Wesley M. Hochachka, Joshua I. Engel, Jeffrey A. Bell, Vasyl V. Tkach, John M. Bates, Shannon J. Hackett, Jason D. Weckstein

**Affiliations:** 1 Department of Ecology and Evolutionary Biology, College of Agricultural and Life Sciences, Cornell University, Ithaca, New York, United States of America; 2 Department of Population Medicine and Diagnostic Sciences, College of Veterinary Medicine, Cornell University, Ithaca, New York, United States of America; 3 Cornell Lab of Ornithology, Cornell University, Ithaca, New York, United States of America; 4 Department of Zoology, Field Museum of Natural History, Chicago, Illinois, United States of America; 5 Department of Biology, University of North Dakota, Grand Forks, North Dakota, United States of America; 6 Academy of Natural Sciences of Drexel University, Ornithology Department and Department of Biodiversity, Earth, and Environmental Sciences, Philadelphia, Pennsylvania, United States of America; Bernhard Nocht Institute for Tropical Medicine, GERMANY

## Abstract

Avian host life history traits have been hypothesized to predict rates of infection by haemosporidian parasites. Using molecular techniques, we tested this hypothesis for parasites from three haemosporidian genera (*Plasmodium*, *Haemoproteus*, and *Leucocytozoon*) collected from a diverse sampling of birds in northern Malawi. We found that host life history traits were significantly associated with parasitism rates by all three parasite genera. Nest type and nest location predicted infection probability for all three parasite genera, whereas flocking behavior is an important predictor of *Plasmodium* and *Haemoproteus* infection and habitat is an important predictor of *Leucocytozoon* infection. Parasite prevalence was 79.1% across all individuals sampled, higher than that reported for comparable studies from any other region of the world. Parasite diversity was also exceptionally high, with 248 parasite cytochrome *b* lineages identified from 152 host species. A large proportion of *Plasmodium*, *Haemoproteus*, and *Leucocytozoon* parasite DNA sequences identified in this study represent new, previously undocumented lineages (n = 201; 81% of total identified) based on BLAST queries against the avian malaria database, MalAvi.

## Introduction

Vector-borne pathogens are responsible for a vast number of diseases that negatively impact animal and human health. Many of these pathogens can infect multiple hosts, and prevalence varies among susceptible host species [1, 2]. Although factors contributing to variation in host susceptibility are complex [3–6], host-vector encounter rates are known to play an important role in the transmission of some vector-borne pathogens [7, 8]. Host life history traits and behaviors have been associated with host-vector encounter rates [9, 10], and may be an important filter for transmission of vector-borne pathogens such as malarial parasites. Empirically identifying which life history traits have the greatest filtering effects on transmission within vector-borne disease systems will improve epidemiological models [4], as well as our understanding of the fundamental ecological and evolutionary mechanisms underlying pathogen virulence, prevalence within populations, and host susceptibility across taxa.

Avian models have provided important insights into the relationships between host life history traits and transmission of vector-borne pathogens such as the bacterial Lyme disease agent *Borrelia burgdorferi*, [11, 12], St. Louis encephalitis [13], West Nile virus (WNV) [7, 8, 14], Western equine encephalomyelitis [15], and Eastern equine encephalitis [16]. Among other host life history traits, those related to nesting behavior may be particularly relevant to parasite transmission. For altricial nestlings, a combination of naïve immune system, bare skin exposed by poor feather coverage, and a stationary position in the nest are generally expected to increase susceptibility to vector-borne pathogens [9, 10, 17–19]. Likewise, adult hosts, due to their relatively stationary position during brooding and the accumulation of chemical cues for the vectors of pathogens, may be more highly exposed [20, 21]. For example, a “host funnel” for WNV at the end of the nesting season produces an increase in contact rates between the ornithophilic vectors, *Culex* spp. mosquitoes (Diptera: Culicidae) and nesting birds [22], resulting in amplification of the pathogen [23].

Avian haemosporidian parasites (phylum Apicomplexa) have served as important models for the study of host-parasite interactions [24–27], parasite-mediated selection [28–31], and genetics and epidemiology of human malaria [32–34]. Avian haemosporidia, which include malaria parasites (*Plasmodium*) and closely-related genera (*Haemoproteus*, *Leucocytozoon*), maintain labile associations with vertebrate hosts through time and space [35–37]. Diversity among haemosporidian parasites has been drastically underestimated by microscopy-based studies [38, 39] and a growing number of molecular studies have now identified over 1,500 unique parasite lineages [36, 40–47].

Tropical bird communities and their haemosporidian parasites are a good model system for investigating the role of avian life history traits on probabilities of infection with vector-borne pathogens. Tropical bird species occupy a broad range of habitats, often in close proximity to each other, and exhibit a wide variety of flocking behaviors, habitat preferences, nest types, and nest placements. This system is therefore suitable for testing the ability of certain host life history traits to predict rates of parasitism across a broad spectrum of hosts. Furthermore, improved sampling of little-studied tropical avian haemosporidia, particularly outside of the Western Hemisphere, which have been poorly studied relative to their temperate counterparts, has important implications for our ability to understand haemosporidian ecology and evolution on a broader biogeographic scale [48].

In this study, we tested the hypothesis that life history traits of Afrotropical birds predict rates of parasitism by three haemosporidian parasite genera (*Plasmodium*, *Haemoproteus*, and *Leucocytozoon*). We included in our analyses traits that are known to be associated with host-vector encounter rates—nest type, nest placement, and flocking behavior. We have combined broad taxonomic sampling of host species from a wide variety of habitats and life histories in Northern Malawi with the application of rigorous PCR-based methods to detect rates of parasitism. Additionally, we describe an unprecedented number of novel parasite lineages and their associations with birds in Malawi.

## Methods

### Field Sites and Sampling

**Fig 1:**
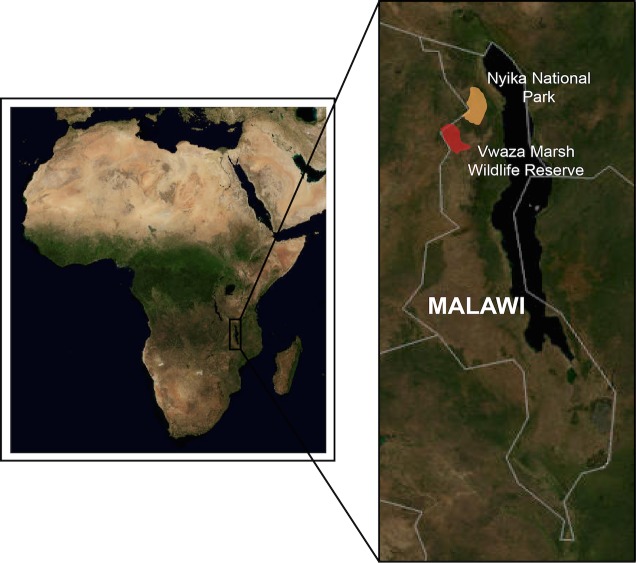
Map of sampling locations in northern Malawi. Vwaza Marsh Wildlife Reserve, red; Nyika National Park, orange. Image credit: USGS National Map Viewer.

Sampling was carried out from October—November of 2009 in Vwaza Marsh Wildlife Reserve and Nyika National Park in Malawi (Fig. 1) as part of a larger project to assess parasite and pathogen diversity in African birds. Birds were sampled from a variety of habitats at each field site [49] to thoroughly assess host and parasite diversity (Table 1). For statistical analyses, these habitats were grouped into five broad classes: aquatic, evergreen forest, forest edge, grassland/marsh, and riparian forest/woodland. Host voucher specimens were collected and deposited at the Field Museum of Natural History (Chicago, IL) and the Museums of Malawi (Blantyre, Malawi). The Malawi Department of Forestry (License No. 3/12/2007/1, granted 10 July 2009, valid 10 July 2009–10 July 2010) and the Department of National Parks and Wildlife (Ref. No. NPW/2/1/12, granted 6 October 2009, valid October-November 2009) provided permits for collecting vertebrate specimens in Vwaza Marsh Wildlife Reserve (Base camp: 11°08.03’S, 33°39.31’E; elevation 1092m) and Nyika National Park (Base camp: 10°73.33’S, 33°96.67’E; elevation 2233m). Birds were euthanized via thoracic compression following the euthanization guidelines published in the American Ornithologists' Union's "Guidelines to the Use of Wild Birds in Research." (http://www.nmnh.si.edu/BIRDNET/guide/). The protocols described in this document were approved for use by the Field Museum’s Institutional Animal Care and Use Committee. We did not collect specimens of endangered or threatened species. Avian specimens are being used in ongoing morphological studies of geographic variation in African birds.

**Table 1:** Sampling localities and habitat types in Malawi.

Field Site	Habitats Sampled
Vwaza Marsh Wildlife Reserve	Lake edge
Latitude: 11° 08.033’ S	Marsh
Longitude: 33° 39.307’ E	Miombo woodland
Elevation: 1071–1170 m	Riparian woodland
Nyika National Park	Evergreen forest
Latitude: 10° 35.307’ S	Miombo woodland
Longitude: 33° 48.670’ E	Montane grassland (open)
Elevation: 1647–2347 m	Montane grassland (scrub-lined watercourse)
	Riparian woodland

In total, we sampled 532 birds from 16 orders, 50 families, 100 genera, and 152 species. Blood was collected via brachial venipuncture from live birds and stored on Whatman FTA Classic Cards (GE Healthcare, Piscataway, NJ). Of the 532 birds originally sampled, data from 469 individuals were included in our statistical analyses of host life history traits, after removing data from: Palearctic migrants that do not nest in Africa, nest-parasitic species for which we could not classify nest height, and domestic species (i.e. *Gallus gallus domesticus*). We also excluded individuals sustaining coinfections from our analyses when parasite identities could not be differentiated (see section 2.3). Lastly, “aquatic” host species were removed from analyses of *Haemoproteus* and *Leucocytozoon* parasitism rates, as none of these host individuals were infected by these two parasite genera. This was necessary because the models containing habitat as a predictor did not converge to solutions when no variation in response was found within this habitat class.

### Molecular detection of haemosporidian parasites

Parasites were detected via replicated applications of a nested polymerase chain reaction (PCR) method based on Hellgren *et al*. [50] and Waldenström *et al*. [51]. Genomic DNA was extracted from whole blood stored on Whatman FTA Classic Cards using Qiagen Blood and Tissue Mini kits, following the protocol for dried blood spots (Qiagen, Valencia, CA). Parasitemia, or the quantity of parasites circulating in an individual host, can vary highly between patent and chronic stages of infection. Haemosporidian infections in birds can persist in a chronic stage for years [52], and therefore the starting quantity of parasite DNA in blood samples may be low, leading to a high occurrence of false negatives [53, 54]. We applied PCR protocols multiple times to all individual samples, which had the effect of increasing probability of detecting low-level infections that were not found in all of the multiple repeated tests. To our knowledge, our application of triplicate screens is a novel approach for improving detection of haemosporidian parasites from blood samples. For detection of *Plasmodium* and *Haemoproteus*, PCR screens targeting the mitochondrial cytochrome *b* gene (cyt *b*) were conducted three times from the extracted DNA using identical conditions, and for detection of *Leucocytozoon*, PCR screens targeting cyt *b* were conducted using each of two sets of *Leucocytozoon*-specific primers a single time (S1 Table). Negative controls were included in all PCR runs to control for false positives due to the high sensitivity of nested PCR (sensu Hellgren *et al*. [55]). PCR products identified as positive for haemosporidian infection were prepared for direct sequencing by enzymatic ExoSAP-IT (Affymetrix) or gelase b-agarose gel-digesting purification (Epicentre Technologies, Madison, Wisconsin). Purified PCR products were sequenced using ABI Prism Dye Terminator Cycle Sequencing Ready Reaction Kits with AmpliTaq DNA Polymerase FS (Perkin-Elmer) and run on an ABI 3730 Automated DNA Sequencer (Applied Biosystems, Foster City, California) using both the forward and reverse primers HAEMF and HAEMR2 for *Plasmodium* and *Haemoproteus*, and either HAEMFL and HAEMR2L [50] or L545F and L825R (this study; S1 Table) for *Leucocytozoon*. Sequence data were edited using Sequencher v.5.0.1 (GeneCodes, Ann Arbor, Michigan) and Geneious v.6.1.6 (Biomatters).

Although microscopic methods may be less sensitive than molecular methods [53, 56], we also visually screened blood films for the presence of malarial parasites. Blood films for 431 of the 532 birds sampled were prepared in the field and fixed with 100% methanol. Blood films were then stained with Giemsa and screened for infection by *Plasmodium*, *Haemoproteus*, or *Leucocytozoon* (*Haemoproteus* and *Plasmodium* identifications were scored without distinguishing between genera). Blood films were screened under 1000× magnification, and 100 fields were screened for each film. Blood films from individuals that screened positive for haemosporidian infection based on PCR, but were negative by the initial round of microscopy, were subjected to an additional round of microscopic screening at 1000×. In the second round of microscopy, 200 fields were examined for each film. We found molecular methods to be more sensitive, with false negatives occurring in <0.2% of molecular screens with respect to microscopically identified positives, whereas false negatives occurred in >37% and >55% of microscopic screens for *Leucocytozoon* and *Plasmodium*/*Haemoproteus* infections respectively. Thus, we chose to rely solely on infections detected by molecular methods for our statistical analyses.

### Identification of haemosporidian parasite lineages

In cases where multiple parasite lineages were detected within one individual host, unique DNA sequences were considered to correspond to individual parasites (i.e., if an individual screened positive for the presence of malaria parasites two out of three times, both PCR products were sequenced; if these sequences differed, and did not contain ambiguities or double peaks, they were considered unique parasite lineages that coinfected the same host). If data from any positive PCR contained sequence ambiguities that could not be differentiated (i.e., two or more clean double peaks on the chromatogram), the host was scored as having an ambiguous coinfection. Relatively few parasite sequences were ambiguous (<12% of total coinfections detected) after performing multiple rounds of PCR amplification and sequencing. Ambiguous sequences and associated host data were removed from subsequent statistical analyses.

*Plasmodium*, *Haemoproteus*, and *Leucocytozoon* sequences were collapsed to unique haplotypes, which were then subjected to a BLAST search against the MalAvi database [57] to identify parasite lineages. Lineages previously identified in the MalAvi database (100% pairwise identity compared to known sequences) were named accordingly, and novel lineages from this study (<100% pairwise identity compared to published sequences) were assigned names prefixed according to geographic location (Africa = “AFR” in S2 Table). Lineages identified in this study are available both on GenBank (Accession Numbers KM056404-056650) and the MalAvi database [57].

### Selection and scoring of life history and ecological parameters for host species

We included life history traits in our models that are linked to host-vector encounter rates during the nesting period (e.g. nest type, and nest height) because the blood parasites in our study are vector-borne and nestlings have been shown to be more vulnerable to host-seeking vectors [9, 10, 17–19]. We also included host flocking behavior, because of the known role of kairomones (olfactory cues emitted by hosts) in attracting the dipteran hosts of haemosporidian parasites [58–60], and habitat, to account for variation of haemosporidian and vector prevalences at sampled sites. Nest type was categorized as open cup, closed cup, or cavity. Closed cup includes nests other than cavities that are built with a covering, creating an enclosed chamber, such as pendant or spherical nests built by weavers. Nest location was categorized as ground, understory, canopy/sub-canopy, or cliff/bank. Species were classified as having understory nests if nests occur predominantly <3 meters above the ground; nests above 3 meters were classified as canopy/sub-canopy. Flocking behavior was categorized as solitary (species that predominantly forage singly or in pairs, e.g., Cuculidae); single-species flock (including species that forage primarily as family groups, e.g., *Plocepasser rufocapulatus*, or species that form larger single-species flocks, e.g., *Lamprotornis chalybeus)*; or mixed-species flock (e.g., *Parus* and many other forest and woodland passerines). Habitat was categorized as described in methods section 2.1. Parameter scores for all species sampled in our study can be found in S3 Table. Data for these parameters were obtained from *The Birds of Africa* series [61–67]. Four species in our study lacked nesting data (*Cisticola njombe*, *Serinus citrenilloides*, *Serinus striolatus*, *Laniarius fuelleborni*). For these species, nesting habits are consistent for all members of their respective genera, so values were inferred from their close relatives. Species that commonly occur in multiple habitats (e.g., *Zosterops senegalensis*) were scored according to the habitat from which they were collected in our study.

### Statistical Analyses

We used generalized linear mixed models to identify which combination of host life history, behavioral, and ecological factors best predicted the probability of an individual bird being parasitized. Independently for each parasite genus (*Plasmodium*, *Haemoproteus*, and *Leucocytozoon*), we assessed the ability of 15 different logistic regression models (Table 2) to predict the binomial response variable, uninfected vs. infected. All fixed effects—nest type, nest location, flocking behavior, and habitat—were treated as categorical variables. To account for host phylogenetic constraints on parasitism due to factors that we did not measure, we included three nested random effects: host family, host genus nested within host family, and host species nested within host genus nested within host family (Table 3). This approach allowed us both to account for statistical non-independence in our data owing to host phylogenetic constraint, and to identify the taxonomic level at which these unexplained effects (if any) are occurring. Because our samples came from two sites at different elevations during different months, Vwaza Marsh Wildlife Reserve (1071–1170 m; October) and Nyika National Park (1647–2347 m; November), we statistically controlled for any differences in baseline levels of parasitism between sites/months by including site as a fixed effect in all of our analyses.

**Table 2:** Fixed effects in the set of 15 models and the relative support of data for these models (ΔAIC_C_ values) from AIC-based multi-model comparisons. We used these comparisons to identify life history traits associated with rates of haemosporidian parasitism of avian hosts. An “X” indicates that a given trait (column) was used as a fixed effect, categorical variable in a given model (row). For each parasite genus the model with the ΔAIC_C_ value of zero is the best-supported model.

Model #	Nest Location^a^	Nest Type^b^	Flocking Behavior^c^	Habitat^d^	Study Site^e^	ΔAIC_C_ Plasmodium	ΔAIC_C_ Haemoproteus	ΔAIC_C_ Leucocytozoon
1	X				X	5.224	2.435	11.471
2		X			X	1.852	3.096	24.121
3			X		X	0.222	5.594	22.932
4				X	X	7.633	10.063	11.162
5	X	X			X	2.276	1.680	12.450
6	X		X		X	3.690	0.662	12.770
7	X			X	X	11.716	8.230	1.939
8		X	X		X	0	2.539	24.176
9		X		X	X	7.274	6.723	9.258
10			X	X	X	6.020	7.331	13.148
11	X	X	X		X	0.744	0	14.211
12	X	X		X	X	13.336	5.140	0
13	X		X	X	X	10.380	4.579	5.262
14		X	X	X	X	4.615	3.951	12.133
15	X	X	X	X	X	6.896	4.038	3.719

^a^Nest Location: Ground, Understory, Canopy/Sub-canopy, Cliff or bank

^b^Nest Type: Open cup, Closed cup, Cavity

^c^Flocking Behavior: Solitary, Monospecific flock or family group, Mixed-species flock

^d^Habitat: Woodland, Grassland or Marsh, Forest edge, Aquatic, Evergreen forest

^e^Study Site: Vwaza Marsh Wildlife Reserve, Nyika National Park

**Table 3:** Tests of statistical significance of host phylogenetic constraints on probability of parasitism. Phylogenetic effects were examined in our analyses by including nested random effects of host family, genus (within family), and species (within genus) on the probabilities of parasitism with each of the three genera of parasites. Statistical tests were likelihood ratio tests each with a single degree of freedom.

Parasite genus	Host taxonomic level	Chi-squared value	P-value
*Plasmodium*			
	Family	4.53	0.03
	Genus (within Family)	0.00	1
	Species (within Genus)	0.00	1
*Haemoproteus*			
	Family	0.00	0.97
	Genus (within Family)	1.27	0.26
	Species (within Genus)	6.40	0.01
*Leucocytozoon*			
	Family	0.30	0.59
	Genus (within Family)	1.82	0.18
	Species (within Genus)	0.00	0.97

Our main conclusions are based on the approach to model comparisons and weighted averaging outlined by Burnham and Anderson [68]. Models were ranked by importance based on weights calculated using Akaike’s Information Criterion (AIC) (Table 4). We assessed the relative importance of each fixed-effect predictor variable by calculating the cumulative support for each predictor as the sum of weights of all models containing that predictor. The effect of each predictor and its precision were estimated by calculating weighted average (“model-averaged”) regression coefficients, standard errors, and 95% confidence limits (Table 5). To make qualitative comparisons among all categories, we produced graphs illustrating the size of each effect (see below) for which we found significant regression coefficients (i.e., coefficients with model-averaged confidence limits not overlapping zero).

**Table 4:** AIC-based support for fixed effects. Values are sums of model weight values for all models in the set (Table 2).

Fixed Effect	*Plasmodium*	*Haemoproteus*	*Leucocytozoon*
Nest type	0.68	0.65	0.72
Nest location	0.34	0.78	0.99
Flocking Behavior	0.78	0.70	0.14
Habitat	0.06	0.15	1

**Table 5:** Model-averaged regression coefficients, standard errors, and 95% confidence limits used to estimate effects of predictors and precision of effects. Note that for each predictor, the regression coefficients are interpreted as describing deviations in parasitism rates from a reference category whose effect is subsumed into the intercept term of the statistical model. Thus, although we can compare parasitism rates of understory nesting species with ground nesting species (the reference category for nest location) using the model-averaged regression coefficients, we cannot use these coefficients to directly compare parasitism rates of understory and canopy nesters.

		*Plasmodium*			*Haemoproteus*			*Leucocytozoon*		
		Model-averaged beta and			Model-averaged beta and			Model-averaged beta and		
		95% confidence limits			95% confidence limits			95% confidence limits		
Parameter	Parameter description	Beta	Lower CL	Upper CL	Beta	Lower CL	Upper CL	Beta	Lower CL	Upper CL
(Intercept)		−1.53	−2.49	−0.57	−1.14	−2.64		0.37	−3.48	−5.21	−1.74
Nest Location	Understory	0.38	−0.38	1.14	0.39	−0.81		1.60	1.83	0.64	3.02
Nest Location	Canopy/Subcanopy	0.62	−0.29	1.52	1.55	0.28		2.83	2.35	0.97	3.72
Nest Location	Cliff/Bank	1.94	0.07	3.81	0.51	−1.97		2.99	−0.49	−3.32	2.34
Nest Type	Closed cup	0.71	0.06	1.36	−1.12	−2.07		−0.17	−0.28	−1.18	0.62
Nest Type	Cavity	−0.24	−1.08	0.6	0.42	−0.65		1.50	1.38	0.19	2.57
Flocking Behavior	Same-species flock or family group	0.5	−0.12	1.12	−0.87	−1.75		0.02	−0.12	−0.94	0.69
Flocking Behavior	Mixed-species flock	0.89	0.17	1.61	−1.28	−2.32		−0.23	0.19	−0.74	1.12
Habitat	Grassland/Marsh	−0.27	−1.07	0.54	−0.37	−1.56		0.83	1.67	0.37	2.97
Habitat	Forest edge	−0.02	−0.77	0.73	0.27	−0.74		1.28	1.47	0.30	2.64
Habitat	Aquatic*	−0.68	−3.29	1.94	NA	NA		NA	NA	NA	NA
Habitat	Evergreen forest	−0.53	−1.44	0.38	0.52	−0.67		1.71	2.49	1.25	3.73
Site	Vwaza Marsh Wildlife Reserve	0.84	0.36	1.31	−0.08	−0.84		0.68	−1.38	−2.13	−0.62

*Aquatic habitat was only included as a fixed effect for *Plasmodium* analysis, as zero individuals from aquatic habitats were infected by *Haemoproteus* or *Leucocytozoon*.

Although our major conclusions are based on the multi-model procedures outlined above, two additional results are based on examining output from single models. First, to display variation in the expected probabilities of parasitism for each haemosporidian genus, we calculated least-squares mean probabilities of parasitism from the single model in each set that contained all of the predictor variables identified as important based on model-averaged coefficients and their confidence intervals (see previous paragraph). Second, we used these same models (one for each haemosporidian genus) to model parasitism rates, then calculated the statistical significance of each of the three random effects used to account for unexplained host phylogenetic constraints. The significance of each random effect was determined using a likelihood ratio test that compared the full model (all fixed and random effects present) with a model in which only the focal random effect was removed from the full list.

All models were fit using restricted maximum likelihood implemented with the glmer function from the lme4 package [69] within R (version 3.0.2; R Development Core Team 2013). Model weights and model-averaged regression coefficients were calculated using the aictab.mer and modavg.mer functions found in the R package AICcmodavg [70]. We used the R package lsmeans to calculate least-squares means and their confidence intervals. The statistical significance of host phylogeny (random effects) was calculated with Chi-squared likelihood ratio tests using the rand function in the lmerTest package [71] within R.

## Results

### General patterns, model selection and effect of life history traits on rates of parasitism

**Fig 2:**
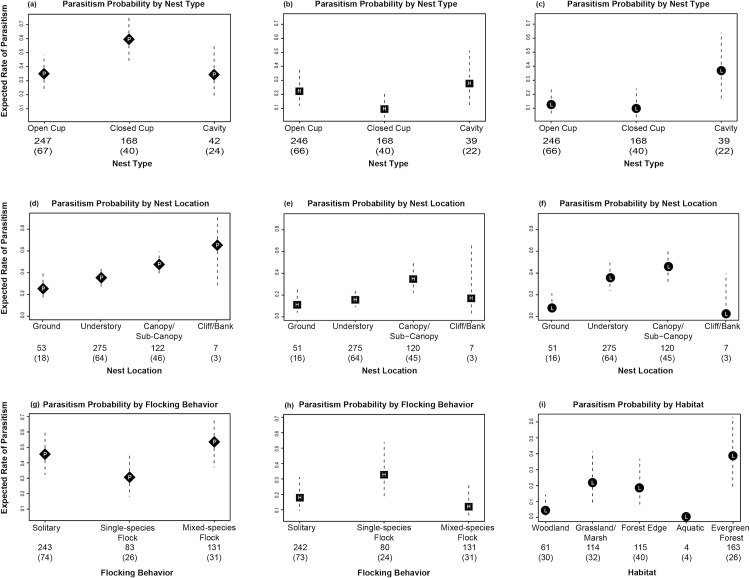
Predicted (least-squares mean) probabilities of parasitism and their 95% confidence intervals. Expected rates of parasitism illustrated according to (a—c) host nest type, (d—f) host nest location, (g—h) host flocking behavior, and (i) habitat. For all panels, *Plasmodium* is represented by a black diamond and the letter “P”, *Haemoproteus* is represented by a black square and the letter “H”, and *Leucocytozoon* is represented by a black circle and the letter “L”. Number of individuals and species comprising each trait are listed below their respective traits (number of individuals above, number of species below in parentheses). Note that the parasitism rate of zero has been plotted for the aquatic habitat without confidence intervals; because the four individual aquatic-habitat birds sampled lack *Leucocytozoon* infections and therefore could not be used in the statistical analysis (as noted in the Methods section) Thus no measure of statistical confidence is associated with this aquatic habitat plotted point.

Host nest type and location were important predictors of infection for all three parasite genera (Table 5). We also found that host flocking behavior was an important predictor of *Plasmodium* and *Haemoproteus* infections, and that habitat was an important predictor of infection by *Leucocytozoon* only (Fig. 2A-I). In general, cisticolas (Cisticolidae), weavers (Ploceidae), and estrildid finches (Estrildidae), all of which build closed cup nests and thrive in a range of habitats, were frequently parasitized by *Plasmodium* (Table 6). Greenbuls (Pycnonotidae) and white-eyes (Zosteropidae), on the other hand, experienced low rates of parasitism by *Plasmodium* and high rates of parasitism by *Leucocytozoon* and *Haemoproteus*. Pigeons and doves (Columbidae) sampled in this study were primarily parasitized by *Haemoproteus* parasites in the subgenus *Parahaemoproteus* (unpublished molecular analyses). However, one individual (African olive pigeon) was parasitized by a novel *Haemoproteus* lineage that was most closely related to the strigiform parasite *Haemoproteus syrnii* (subgenus *Haemoproteus*). Notably, no nightjars (Caprimulgiformes) sampled in this study (n = 8) were parasitized. Caprimulgiform species sampled included Fiery-necked Nightjar (*Caprimulgus pectoralis*), Square-tailed Nightjar (*Scotornis fossii*), and Ruwenzori Nightjar (*Caprimulgus poliocephalus*), all of which are solitary, open cup, ground-nesting species. Host-parasite association data for all individuals sampled are summarized in S2 Table.

**Table 6:** Rates of parasitism by higher level avian taxa (Order and Family).

Host	Host	Host Species	Host Samples	Plasmodium		Haemoproteus		Leucocytozoon		Uninfected		Genus Unknown	
Order	Family	(n)	(n)	Infected (n)	%	Infected (n)	%	Infected (n)	%	(n)	%	(n)	%
Anseriformes	Anatidae	2	3	1	33%	0	0%	0	0%	2	67%	0	0%
Bucerotiformes	Bucerotidae	2	2	1	50%	0	0%	2	100%	0	0%	1	50%
Caprimulgiformes	Caprimulgidae	3	7	0	0%	0	0%	0	0%	7	100%	0	0%
Ciconiiformes	Ardeidae	2	3	0	0%	2	67%	0	0%	0	0%	1	33%
	Scopidae	1	1	0	0%	1	100%	1	100%	0	0%	0	0%
Coliiformes	Coliidae	1	2	1	50%	0	0%	0	0%	1	50%	0	0%
Columbiformes	Columbidae	5	10	0	0%	7	70%	1	10%	3	30%	0	0%
Coraciiformes	Alcedinidae	3	5	1	20%	3	60%	1	20%	1	20%	1	20%
	Coraciidae	1	1	0	0%	0	0%	1	100%	1	100%	0	0%
	Meropidae	2	8	1	13%	3	38%	1	13%	4	50%	0	0%
Cuculiformes	Cuculidae	3	4	1	25%	2	50%	0	0%	1	25%	0	0%
Falconiformes	Accipitridae	2	2	1	50%	1	50%	0	0%	0	0%	0	0%
Galliformes	Numididae	1	1	0	0%	1	100%	0	0%	0	0%	0	0%
	Phasianidae	3	6	1	17%	1	17%	2	33%	3	50%	1	17%
Gruiformes	Rallidae	1	2	2	100%	0	0%	0	0%	0	0%	0	0%
Musophagiformes	Musophagidae	1	2	0	0%	1	50%	0	0%	0	0%	1	50%
Passeriformes	Alaudidae	1	3	2	67%	0	0%	0	0%	1	33%	0	0%
	Cisticolidae	11	45	27	60%	4	9%	8	18%	9	20%	5	11%
	Corvidae	1	3	1	33%	1	33%	1	33%	1	33%	0	0%
	Dicruridae	1	5	2	40%	1	20%	0	0%	1	20%	1	20%
	Emberizidae	2	2	1	50%	1	50%	0	0%	0	0%	0	0%
	Estrildidae	11	44	18	41%	5	11%	15	34%	13	30%	2	5%
	Eurylaimidae	1	1	0	0%	0	0%	1	100%	0	0%	0	0%
	Fringillidae	4	23	8	35%	6	26%	9	39%	4	17%	4	17%
	Hirundidae	3	5	3	60%	1	20%	1	20%	0	0%	0	0%
	Laniidae	1	1	0	0%	0	0%	0	0%	0	0%	1	100%
	Malaconotidae	6	15	12	80%	0	0%	6	40%	2	13%	2	13%
	Monarchidae	2	5	1	20%	1	20%	3	60%	0	0%	0	0%
	Motacillidae	1	2	1	50%	0	0%	0	0%	1	50%	0	0%
	Muscicapidae	12	62	27	44%	14	23%	24	39%	14	23%	7	11%
	Nectariniidae	8	28	6	21%	14	50%	6	21%	6	21%	1	4%
	Oriolidae	1	2	0	0%	1	50%	0	0%	0	0%	1	50%
	Paridae	2	2	2	100%	0	0%	1	50%	0	0%	1	50%
	Passeridae	3	7	3	43%	2	29%	1	14%	1	14%	0	0%
	Platysteiridae	2	20	8	40%	3	15%	8	40%	4	20%	5	25%
	Ploceidae	9	57	38	67%	4	7%	16	28%	5	9%	6	11%
	Pycnonotidae	5	44	12	27%	15	34%	28	64%	4	9%	7	16%
	Remizidae	1	1	0	0%	0	0%	0	0%	1	100%	0	0%
	Stenostiridae	1	1	0	0%	0	0%	0	0%	1	100%	0	0%
	Sturnidae	4	12	4	33%	7	58%	4	33%	2	17%	1	8%
	Sylviidae	9	33	8	24%	9	27%	6	18%	13	39%	2	6%
	Timaliidae	2	6	1	17%	1	17%	4	67%	0	0%	2	33%
	Turdidae	4	15	5	33%	3	20%	8	53%	3	20%	1	7%
	Viduidae	1	1	0	0%	0	0%	0	0%	1	100%	0	0%
	Zosteropidae	1	14	3	21%	13	93%	10	71%	0	0%	1	7%
Piciformes	Indicatoridae	3	5	2	40%	1	20%	3	60%	3	60%	0	0%
	Picidae	2	3	1	33%	1	33%	1	33%	1	33%	0	0%
	Ramphastidae	3	6	0	0%	3	50%	0	0%	2	33%	1	17%
Psittaciformes	Psittacidae	1	1	1	100%	0	0%	0	0%	0	0%	0	0%
Trogoniformes	Trogonidae	1	2	1	50%	1	50%	1	50%	0	0%	1	50%

Based on confidence limits around the model-averaged regression coefficients, infection by *Plasmodium* was significantly higher in closed cup nesting birds (n = 168 individuals sampled; 40 host species) relative to cavity-nesting (n = 39 individuals sampled; 22 host species) or open cup nesting host species (n = 247 individuals sampled; 67 host species) (Fig. 2A-C). In contrast, infection by *Haemoproteus* was significantly lower in closed cup nesting birds relative to cavity-nesting or open cup nesting host species. Lastly, cavity-nesting species had a higher probability of *Leucocytozoon* infection than either open cup or closed cup nesting host species.

Parasitism by all three genera of haemosporidia generally increased with increasing nest height, excluding cliff/bank nesters (Fig. 2D-F). Nest location was the most important trait determining *Haemoproteus* infection rate, and was the second most important trait determining *Leucocytozoon* infection rate, based on the summed AIC-weights of the models containing these predictor variables. However, support for an effect of nest location on *Plasmodium* infection rates was relatively low compared to other variables (flocking behavior and nest type), based on this same criterion. Birds nesting in the canopy and sub-canopy were more likely than ground nesters to be infected by *Haemoproteus* or *Leucocytozoon*, and those nesting in the understory also were more likely to be infected by *Leucocytozoon* than were ground-nesting birds. Birds nesting on cliffs or banks were more likely than ground nesters to be infected by *Plasmodium*, but support for this effect was comparatively weak (Table 5).

Flocking behavior was associated with parasitism rates by *Plasmodium* and *Haemoproteus*, but had no effect on *Leucocytozoon* parasitism rates. Birds in single-species flocks had lower rates of *Plasmodium* infection and higher rates of *Haemoproteus* infection, relative to solitary birds or birds living in mixed-species flocks (Fig. 2G-H). Inversely, habitat was the most important predictor affecting *Leucocytozoon* infection rates (Fig. 2I), but habitat had no effect on *Plasmodium* or *Haemoproteus* infection rates. The probability of infection by *Leucocytozoon* was highest in birds of evergreen forest habitat (n = 163; 26 host species), and was higher for birds of grassland/marsh (n = 114; 32 host species) and forest edge (n = 115; 40 species) habitats, relative to birds of woodland habitat (n = 61; 30 host species).

We found no host phylogenetic effect on rates of *Leucocytozoon* infection, but random effects in our models suggest that there may be additional, phylogenetically-constrained traits influencing parasitism rates at the host species level for *Haemoproteus*, and at the host family level for *Plasmodium* (Table 3). We also found that the *Plasmodium* infection rate was higher and the *Leucocytozoon* infection rate lower at the dryer, low elevation site (Vwaza Marsh Wildlife Reserve), compared to infection rates at the wetter, higher elevation (Nyika National Park). No effect of site existed for the rate of *Haemoproteus* infection.

### Prevalence and diversity of haemosporidian parasite assemblages in Malawi

Of the 532 individual birds sampled in this study, 421 were infected by one or more haemosporidian parasite lineage. Prevalence among individual hosts was 48.2% for *Plasmodium*, 31.4% for *Haemoproteus* (subgenera. *Haemoproteus* and *Parahaemoproteus*), and 47.0% for *Leucocytozoon* respectively, with a total haemosporidian infection prevalence of 79.1% (Table 7). Two-hundred twenty-two (52.7%) of the infected individuals harbored coinfections. Of these coinfected individuals, 172 (40.9%) were infected by two or more identifiable parasite genera (Table 8). Thirty of the remaining fifty coinfected individuals (11.8% of all coinfections) were infected by at least one *Leucocytozoon* parasite.

**Table 7:** Haemosporidian abundance and diversity.

	*Plasmodium*	*Haemoproteus*	*Leucocytozoon*	Total
Individuals infected (n)	203	132	198	421
% of total infected	48.2%	31.4%	47.0%	79.1%
Novel cytochrome *b* lineages	59	53	89	201
Described cytochrome *b* lineages^a^	22	16	9	47
Total lineages identified	81	69	98	248

^a^See MalAvi database for lineage information [57].

**Table 8:** The distribution of resolved coinfections among three genera of parasites.

	P*P	H*H	L*L	P*H	P*L	H*L	P*H*L	TOTAL
Individuals (n)	28	1	16	10	64	48	6	**172**
% of total infected	6.7%	0.2%	3.8%	2.4%	15.2%	11.4%	1.4%	40.9%

P = *Plasmodium* spp.

H = *Haemoproteus* spp.

L = *Leucocytozoon* spp.

*Plasmodium* and *Leucocytozoon* coinfections occurred most frequently (n = 64 individuals; 15.2% of total infected), followed by *Haemoproteus* and *Leucocytozoon* coinfections (n = 48; 11.4%). Coinfection of a single host by congeneric parasites was rare, occurring most frequently with *Plasmodium* lineages (n = 28; 6.7%), about half as frequently for *Leucocytozoon* lineages (n = 16; 3.8%), and only once with *Haemoproteus* lineages (n = 1; 0.2%). Coinfection of individual hosts by all three parasite genera was also rare (n = 6; 1.4%).

From a total of 152 host species screened, we identified 248 unique haemosporidian mtDNA lineages, 201 (81%) of which have not been reported previously (Table 7), based on BLAST queries of cyt *b* haplotypes against the MalAvi database (Grand Alignment 27 August 2013 version; [57]). New lineages documented in this study included 59 of 81 (72%) *Plasmodium* haplotypes, 53 of 69 (77%) *Haemoproteus* haplotypes, and 89 of 98 (91%) *Leucocytozoon* haplotypes.

## Discussion

Relationships among haemosporidian infection rates and avian hosts’ nesting traits in northern Malawi were consistent with previously hypothesized effects of host nesting biology on the transmission of vector-borne parasites [9, 10, 17–19, 22]. Traits not directly related to nesting biology, such as flocking behavior and habitat, also appeared to have an effect on haemosporidian infection rates. Variation in the reliability of host traits to predict parasitism rates of the three haemosporidian parasite genera underscores the importance of vector biology in determining which host species are parasitized. The large number of novel parasite lineages we identified in our study suggests that either transmission to Palearctic migrant hosts occurs infrequently, that competent vectors of these novel African parasite lineages do not exist in the Palearctic, or that Palearctic migrant species wintering in Malawi nest in parts of the Palearctic that have not been well-surveyed for haemosporidians.

### Association between life history traits and parasite prevalence

Our study is the first to examine the relationship between life history traits and haemosporidian parasitism across a broad taxonomic scale in the Afrotropics, where haemosporidian parasite diversity generally has been thought to be low compared to temperate regions [39]. We found that multiple bird life history traits, some of which are related directly to nesting biology, are significantly associated with rates of parasitism by haemosporidia in Malawi. However, these effects are qualitatively different among the three parasite genera, mirroring studies in temperate regions, which have found mixed support for the ability of ecological variables to predict haemosporidian prevalence [25, 27, 72, 73]. Some have found a positive correlation between nest height and infection prevalence [25, 27], whereas others have found no such correlation, even with comparable sampling from the same general temperate region [73]. With respect to nest type, Palearctic species with closed cup nests have a lower probability of infection by haemosporidian parasites, possibly because closed cup nests physically reduce the access of vectors [39].

Several notable studies have considered the effects of environmental and ecological parameters on parasitism rates in focal Afrotropical bird species. For example, Sehgal *et al*. [74] constructed spatial models using climatological and satellite-based habitat and topography data to predict the prevalence of *Plasmodium* and *Trypanosoma* spp. in the olive sunbird (*Cyanomitra olivacea*). Another Afrotropical study of the olive sunbird and yellow-whiskered greenbul (*Andropadus latirostris*) found differences in parasite prevalence between disturbed and undisturbed habitat in Southern Cameroon [40].

An increasing number of studies examining a broad range of host taxa and their parasites have been conducted recently in the Neotropics, where analyses of host life history traits and ecological parameters are comparable to those we have conducted in Malawi. Two studies conducted in Brazil by Fecchio *et al*. [46, 75] examined the relationships between life history traits and prevalence of two haemosporidian genera (*Plasmodium* and *Haemoproteus*). In the first of these two studies, Fecchio *et al*. [75] analyzed microscopy-based parasite data from 7 host families (16 passerine and 1 non-passerine) and found correlations between parasite prevalence and host traits, including nest type, nest height, and flocking behavior. However, in a later molecular-based study by Fecchio *et al*. [46], only flocking behavior was found to significantly explain rates of parasite prevalence among 17 families examined (12 passerine, 5 non-passerine). Another, long-term Neotropical study in primary lowland forest used molecular methods to detect avian malaria infections in 22 passerine families, and found no correlation between *Plasmodium* or *Haemoproteus* prevalence and nest type [47]. Most recently, a microscopy-based Colombian study spanning 12 years and 41 families (21 passerine, 19 non-passerine) examined the relationship between parasitism rates and avian life history traits including nest height, nest type, foraging height, flocking behavior, and migratory behavior, as well as environmental variables such as elevation, seasonality, and habitat [76]. González *et al*. [76] found that both open and closed cup-nesting species experienced higher rates of parasitism by *Plasmodium*, whereas nest type had no effect on rates of parasitism by *Haemoproteus* or *Leucocotyzoon*. Species nesting at mid-understory height were more likely to be parasitized by *Haemoproteus*, but nest height had no effect on *Plasmodium* or *Leucocytozoon* parasitism rates. Lastly, species that participate in in mixed-species flocks experienced higher rates of parasitism by both *Haemoproteus* and *Leucocytozoon*, but flocking behavior had no effect on *Plasmodium* parasitism rates.

Our results from northern Malawi only partially match findings in the Neotropics. Similar to the results of [75], but in contrast to those of [47], we found that, in Malawi, birds building closed cup nests tend to have higher rates of *Plasmodium* infection and lower rates of *Haemoproteus* infection relative to birds building open cup nests. This pattern is consistent with the hypothesis that mosquitoes depend on kairomones (host-derived chemical cues), such as ammonia, 1-octen-3-ol, and CO_2_ compounds [60], to locate hosts for bloodmeals. Host behaviors that increase the accumulation of such chemical compounds are more likely to attract mosquitoes, and therefore more likely to expose hosts to vector-borne parasites. The accumulation of kairomones in closed cup nests may therefore lead to an increased rate of contact between avian hosts and *Plasmodium*-transmitting mosquitoes [58]. This hypothesis is further supported by reported positive correlations between nestling age, increasing metabolic mass (nestlings and adults), and CO_2_ content within the nest [59]. On the other hand, the lower rate of parasitism by *Haemoproteus* in closed cup nesting birds may be due to the hypothesized dependence of biting midges (the definitive hosts of *Haemoproteus* parasites) on visual cues rather than olfactory cues. Although many vectors rely on a number of sensory stimuli, including olfactory, gustatory, and visual cues [77–81], visual cues have been shown, both naturally and experimentally, to play a more important role than kairomones in the host-seeking responses of biting midges [82, 83]. Additional support for the prioritization of visual over olfactory cues comes from the observation that biting midge numbers are significantly reduced in outdoor areas that are covered, such as stables or sheds [84], which Bishop [82] attributes to the blocked vision of host-seeking midges.

Flocking behavior of birds from Malawi had significant and inverse associations with the probability of *Plasmodium* and *Haemoproteus* infections. Social species living in mixed- and single-species flocks have been reported to experience higher rates of *Haemoproteus* parasitism [46, 75]. However, in our study system we did not find a strong relationship between higher infection rates and species’ sociality, per se. Instead, we found that bird species aggregating in single-species flocks experienced a lower probability of infection by *Plasmodium* parasites and a higher probability of infection by *Haemoproteus* parasites, relative to birds that are solitary or living in mixed-species flocks. This result is the opposite of that found in Colombian birds by González *et al*. [76], who showed that birds of mixed-species flocks experienced higher rates of parasitism by *Haemoproteus*. A recent study by Janousek *et al*. [8] suggests that communal roosting in single-species flocks might be influenced by pathogen-mediated selection, driven by host-vector ratios and encounter rates of birds and virus-transmitting *Culex* mosquitoes [8]. Similar mechanisms may influence rates of parasitism by *Plasmodium* parasites, which are also vectored by *Culex* mosquitoes, and could explain the lower rates of infection by *Plasmodium* parasites we observed among birds aggregating in single-species flocks, particularly given that bird species in this category also tend to roost communally (e.g. queleas, widowbirds, starlings, weavers). It is unclear why rates of infection by *Haemoproteus* parasites for birds in single-species flocks are increased. This phenomenon may be related to the behavior and host specificity of vectors. The inverse effects of flocking behavior on *Plasmodium* versus *Haemoproteus* infection rates suggest that the respective vectors of these malaria parasites, the parasites themselves, or some combination thereof, respond differently to the species composition in groups of aggregating hosts.

Similar to Fecchio *et al*. [75], we found higher parasitism rates with increasing average nest height. This correlation is consistent with the observations of Bennett and Fallis [25] from the Nearctic, that vector abundance is vertically stratified, and thus birds nesting at higher strata should experience increased rates of parasitism due to increased contact with vectors. Although this hypothesis has not been tested for ornithophilic vectors in the east African tropics, our results suggest that mechanisms similar to those in the Nearctic may also influence host-vector contact rates in the Afrotropics.

The effect of habitat on parasitism rates can vary from region to region, and may be influenced by many factors in the study design, including the number of taxa sampled, number of habitats compared, and size of the study site/region [42, 45, 75]. We found no effects of habitat on *Plasmodium* or *Haemoproteus* infection rates. However, rate of *Leucocytozoon* infection is strongly affected by habitat. Specifically, when compared to birds from all other habitats sampled (excluding aquatic habitat due to inadequate sample sizes), those living in evergreen forest experienced the highest rates of *Leucocytozoon* infection, whereas birds from woodland habitats experienced the lowest rates of *Leucocytozoon* infection. This effect is likely caused by elements within these habitats that are related to vector ecology, and the same is possibly true for life history or habitat effects on rates of parasitism by *Plasmodium* and *Haemoproteus*. *Plasmodium*, *Haemoproteus*, and *Leucocytozoon* are vectored by different dipteran insects, therefore aspects of vector feeding behaviors must be considered when attempting to discern the underlying causes of variation in rates of parasitism. More research is certainly needed in this area.

### Prevalence, diversity, and geographic range of avian *Plasmodium*, *Haemoproteus*, and *Leucocytozoon* lineages found in Malawi

Total prevalence of haemosporidian parasites in our Malawi samples (78.1%) is high relative to studies of avian haemosporidian prevalence with comparable numbers of host species from other geographic regions (Table 9). If only considering *Plasmodium* and *Haemoproteus*, the prevalence from our Malawi study is 66.6% (43.9% and 25.8% respectively, with many individual hosts harboring coinfections). Ricklefs *et al*. [73] proposed that with sufficient sampling, the number of malaria parasite lineages within a local geographic area should approximate the number of host species. Our data support this hypothesis, finding 149 malaria parasites lineages (*Plasmodium* spp. and *Haemoproteus* spp.) in the 152 bird species sampled, far exceeding malaria parasite diversity previously described for this region by Loiseau *et al*. [42]. Our results also suggest that the Afrotropics harbor greater prevalence and genetic diversity of *Leucocytozoon* parasites than previously found in any other region of the world (Table 9).

**Table 9:** Comparative overview of some studies in avian haemosporidian prevalence and diversity.

Location	Length of study	Host Orders (n)	Host Families (n)	Host Species (n)	Individuals Sampled (n)	% *Plasmodium*	% *Haemoproteus*	% *Leucocytozoon*	Total prevalence among all individuals sampled*	Parasite detection method	Parasite lineages identified (n)	Reference
**AFRICA**												
Malawi	2009	16	50	152	532	50%	23.6%	36.1%	79.1%	PCR	248	This study
Cameroon, Gabon	2002–2004	9	29	93	527	45%	23%	7%	NA	PCR	117	Beadell *et al*. 2009 [36]
Madagascar	1993–2004	7	26	64	947	1.9%	17.4%	9.4%	NA	Microscopy	45	Savage *et al*. 2009 [85]
Cameroon Gabon Tanzania MalawiSouth Africa	NA	1	8	25	1364	20.1%	NA	NA	20.1%	PCR	34	Loiseau *et al*. 2011 [42]
Western Indian Ocean	1999–2002	1	6	21	150	32%	13.3%	NA	45.3%	PCR	16	Ishtiaq *et al*. 2012 [44]
**ASIA**												
Myanmar	1994–2004	5	52	133	335	20.3%	13.4%	NA	34%	PCR & Microscopy	75	Ishtiaq *et al*. 2007 [37]
India				43	183	27.9%	18%	NA				
South Korea				46	181	30.9%	11%	NA				
**AUSTRALIA**												
Australia	2002–2003	3	8	32	219	14%	28%	NA	44%	PCR	78	Beadell *et al*. 2004 [86]
Papua New Guinea	1991–2002			77	209	10%	31%	NA				
New Zealand	2003–2006	3	21	25	820	52.9%	NA	NA	52.9%		8	Ewen *et al*. 2012 [87]
**NORTH AMERICA**												
United States Canada	∼1940–1975	17	55	388	57026	3.8%	19.5%	17.7%	NA	Microscopy	NA	Greiner *et al*. 1975 [88]
Costa Rica	1987–1991	4	15	60	479	0.4%	9.4%	0.4%	11%	Microscopy	NA	Young *et al*. 1993 [89]
United States	1988	1	8	19	935	3.4%	22.8%	1.3%	NA	Microscopy	NA	Garvin & Remsen 1997 [27]
Lesser Antilles	NA	7	17	53	1975	10.3%	36.1%^†^	NA	27.6%	PCR & Microscopy	26	Fallon *et al*. 2005 [90]
United States	1999–2002	2	13	42	757	NA	NA	0%	∼38.6%	PCR & Microscopy	34	Ricklefs *et al*. 2005 [73]
**SOUTH AMERICA**												
Panama	1969–1976	4	36	281	3715	5%	9%	<1%	∼15%	Microscopy	NA	Sousa & Herman 1982 [91]
Bolivia	1988	7	25	135	641	1.1%	1.4%	NA	2.5%	Microscopy	NA	Bennett *et al*.1991 [92]
Brazil	2000	1	9	45	275	39.6%	NA	NA	39.6%	PCR & Microscopy	NA	Ribeiro *et al*. 2004 [93]
Guyana	1994–2000	4	10	53	195	24.6%	13.8%	NA	42.1%	PCR & Microscopy	59	Durrant *et al*. 2006 [94]
Uruguay	2002–2003		41	111	322	17.8%	3.7%	NA	24.2%			
Colombia	2001–2002	4	12	75	302	5.6%	2.6%	0.3%	8.6%	Microscopy	NA	Londoño *et al*. 2007 [95]
Chile	2003–2005	NA	NA	26	617	6.5%	5.0%	8.9%	15.4%	PCR & Microscopy	27	Merino *et al*. 2008 [96]
Colombia	2002–2003	5	14	40	136	8.1%	1.5%	21.3%	NA	Microscopy	NA	Rodríguez *et al*. 2009 [97]
Brazil	2007–2009	2	29	122	676	49% combined		NA	49%	PCR & Microscopy	21	Belo *et al*. 2011 [43]
Brazil	2005–2009	2	6	17	772	3.6%	7.1%	0%	10.7%	Microscopy	NA	Fecchio *et al*. 2011 [75]
Venezuela	2011	2	12	24	47	4.3%	6.4%	2.1%	10.6%	PCR	6	Mijares *et al*. 2012 [98]
Brazil	2005–2009	6	18	54	790	4.8%	11.3%	NA	16.1%	PCR	22	Fecchio *et al*. 2013 [46]
Brazil	2000–2006 2010	2	21	194	1545	23%	4.5%	NA	35.3%	PCR	110	Lacorte *et al*. 2013 [45]
Ecuador	2001–2010	1	22	144	2488	9%	6%	NA	21.7%	PCR	65	Svensson-Coelho *et al*. 2013 [47]
Colombia	1999–2011	NA	41	169	246	3.0%	5.0%	5.0%	12.8%	Microscopy	NA	González *et al*. 2014 [76]

*Calculations based on total individuals infected out of total individuals sampled N_i_/N; values are not always directly comparable between studies due to variation in haemosporidian genera examined.

^†^Lineages n = 3 found in doves but not assigned to haemosporidian genus were considered *Haemoproteus* Haemoproteus spp. and included in calculation of % *Haemoproteus* infection.

Our findings underscore the importance of broad host taxonomic sampling, as well as stringent detection methods. We attribute the high prevalence of infection detected in our study, in part, to the application of multiple PCR screens, which frequently detected new unique parasite lineages in each round of PCR. In another recent PCR-based study, Lacorte *et al*. [45] examined haemosporidian diversity in 200 Brazilian bird species from 21 families (predominantly Passeriformes). Of the 89 *Plasmodium* and 22 *Haemoproteus* lineages identified by Lacorte *et al*. [45], 86% constituted new lineages. Similar to our study, Lacorte *et al*. [45] also found relatively high parasite lineage diversity among a large number of host species sampled, although diversity of malaria parasites from Malawian birds was considerably higher.

The majority of parasite lineages we identified in Malawi (n = 201; 81% of total identified) have not been detected previously, and therefore have not been recorded in other geographic regions. Of those lineages we identified that have been described (n = 47), 25 have been found only in Africa, 10 in both Africa and the western Palearctic, 5 only in the western Palearctic, 3 globally, 2 in Asia, 1 in both Africa and the Middle East, and 1 in North America (Fig 2A-C; also see S2 Table; MalAvi database). The high occurrence of novel parasite lineages in our study may indicate endemic parasite species, and also suggests that avian Palearctic migrants are not suitable hosts for a number of malaria parasite lineages with which they geographically overlap during their wintering season in Africa. However, we cannot rule out the possibility that the absence of competent vectors and/or poor sampling in Palearctic regions (e.g., Western Asia) explains this pattern. We speculate that the high parasite diversity we observed, relative to other studies in Africa (e.g., [36, 41, 42]), might be a consequence of Malawi’s biogeographic position as a meeting place of avian taxa from the south, central, and eastern regions of Africa [99]. More data from neighboring regions will help resolve this question. In general, our findings are consistent with those of Lacorte *et al*. [45] and Clark et al. [48], suggesting that haemosporidian parasite diversity is much higher in both the New and Old World tropics than previous surveys [39] concluded on the basis of microscopy.

## Conclusions

We report new avian haemosporidian data from a broad survey of birds from southeast Africa, a relatively underrepresented region in the history of avian haemosporidian research. Using sensitive molecular methods and multiple screenings to overcome potentially low parasitemia levels in our samples, we found unprecedented prevalence of *Plasmodium*, *Haemoproteus*, and *Leucocytozoon* infections across a broad range of birds belonging to 16 orders, 50 families, 100 genera, and 152 species. Our analyses of avian host life history traits revealed a diversity of relationships between life history characteristics and haemosporidian infection rates for three different parasite genera. The positive correlation between nest height and probability of haemosporidian infection appears to be a consistent pattern across geographic regions. Similar to Neotropical studies, we found differing effects of nest type for each haemosporidian parasite genus. Birds with closed cup nests experienced increased rates of *Plasmodium* infection and decreased rates of *Haemoproteus* infection, whereas cavity-nesting birds experienced increased rates of *Leucocytozoon* infection. The effects of these host traits on haemosporidian infection rates have never been studied in the African tropics, and our data demonstrate that this system contains higher parasite diversity and is equally complex, if not more so, than Neotropical and temperate systems. Haemosporidian prevalence in birds from our Malawi study is higher than that found in other tropical regions, and we identify a relatively large number of new parasite lineages (n = 201; 81% of total identified). These results suggest that, as is the case with many other vector-borne pathogens, the Afrotropics is an area of high haemosporidian endemicity and diversity. The high prevalence, diversity, and possible endemicity of haemosporidians in this southeastern African region, as well as a presumably diverse array of dipteran vectors on which they rely for transmission, indicate their suitability as a model for research on vector-borne pathogens. Southeastern Africa is undeniably an important region in which to investigate the mechanisms underlying host-parasite associations, speciation, and the evolution of malaria parasites and other closely related haemosporidians.

## Supporting Information

S1 Table Primers and thermal cycling conditions. (PDF)

S2 Table Host and parasite lineage associations. (PDF)

S3 Table Host species and life history traits. (PDF)
