# Correction: Parasite Prevalence Corresponds to Host Life History in a Diverse Assemblage of Afrotropical Birds and Haemosporidian Parasites

**DOI:** 10.1371/journal.pone.0128851

**Published:** 2015-05-18

**Authors:** Holly L. Lutz, Wesley M. Hochachka, Joshua I. Engel, Jeffrey A. Bell, Vasyl V. Tkach, John M. Bates, Shannon J. Hackett, Jason D. Weckstein

There is an error in the fifth sentence of the first paragraph of the Results section. The correct sentence is: Pigeons and doves (Columbidae) sampled in this study were primarily parasitized by *Haemoproteus* parasites in the subgenus *Haemoproteus* (unpublished molecular analyses). However, one individual (African olive pigeon) was parasitized by a novel *Haemoproteus* lineage that was most closely related to the strigiform parasite *Haemoproteus syrnii* (subgenus *Parahaemoproteus*).

There is an error in the second sentence of the final paragraph of the Discussion section. The correct sentence is: Of those lineages we identified that have been described (n = 47), 25 have been found only in Africa, 10 in both Africa and the western Palearctic, 5 only in the western Palearctic, 3 globally, 2 in Asia, 1 in both Africa and the Middle East, and 1 in North America (also see *S2 Table; MalAvi database).
