# Supplementary material for: Parasite Prevalence Corresponds to Host Life History in a Diverse Assemblage of Afrotropical Birds and Haemosporidian Parasites
Source: PLoS One. 2015 Apr 8;10(4):e0121254. doi: 10.1371/journal.pone.0121254 (PMC4390322; doi:10.1371/journal.pone.0121254)
Supplement: S1 Table — (PDF) [file pone.0121254.s001.pdf]

Table S1. Primers and thermal cycling conditions

Protocol / Parasite genera	Primer	Primer Sequence	Thermal Cycling Conditions*
Nested PCR to amplify 479 bp of *Plasmodium* and *Haemoproteus* spp. cytochrome *b*			
Primer pair 1	HAEMNF[a]	5' - CATATATTAAGAGAATTATGGAG - 3'	[94/30, 50/30, 72/45] x 20
	HAEMNR2[a]	5' - AGAGGTGTAGCATATCTATCTAC - 3'	
Primer pair 2	HAEMF[b]	5' - ATGGTGCTTTCGATATATGCATG - 3'	[94/30, 50/30, 72/45] x 35
	HAEMR2[b]	5' - GCATTATCTGGATGTGATAATGGT - 3'	
Nested PCR to amplify 479 - 526 bp of *Leucotozoons* spp. cytochrome *b*			
Primer pair 1	HAEMNFI[c]	5' - CATATATTAAGAGAAITATGGAG - 3'	[94/30, 51/30, 72/45] x 20
	HAEMNR3[c]	5' - ATAGAAAGATAAGAAATACCATTC - 3'	
Primer pair 2	HAEMFL[c]	5' - ATGGTGTTTTAGATACTTACATT - 3'	[94/30, 51/30, 72/45] x 35
	HAEMR2L[c]	5' - CATTATCTGGATGAGATAATGGIGC - 3'	
Primer pair 2 (modified)	L350F[d]	5' - GGTGTTTTAGATACTTA -3'	[94/30, 51/30, 72/45] x 35
	L890R[d]	5' - TACAATATGTTGAGGTGTTTG - 3'	
Sequencing primers (modified)	L545F[d]	5' - ACAAATGAGTTTCTGGGGA - 3'	
	L825R[d]	5' - GCAATTCCAAATAAACTTTGAA - 3'	

* Temperature (˚C)/time (s) for denaturation, annealing, and extension steps; outer thermal cycling conditions included an initial denaturation period of 94˚C for 3 minutes and a final extension period of 72˚C for 10 minutes

[a] Waldenström et al., 2004

[b] Bensch et al., 2000

[c] Hellgren et al., 2004

[d] This study

References

Bensch S, Stjernman M, Hasselquist D, Örjan Ö, Hannson B, Westerdahl H, et al. Host specificity in avian blood parasites: a study of *Plasmodium* and *Haemoproteus* mitochondrial DNA amplified from birds. Proc Roy Soc Lond B. 2000; 267, doi: 10.1098/rspb.2000.1181.

Hellgren O, Waldenström H, Bensch S. A new PCR assay for simultaneous studies of *Leucocytozoon*, *Plasmodium* and *Haemoproteus* from avian blood. J Parasitol. 2004; 90: 797 - 802.

Waldenström J, Bensch S, Hasselquist D, Östman Ö. A new nested polymerase chain reaction methods very efficient in detecting Plasmodium and Haemoproteus infections from avian blood. J Parasitol. 2004; 90: 191 – 194.
