# Supplementary material for: Parasite Prevalence Corresponds to Host Life History in a Diverse Assemblage of Afrotropical Birds and Haemosporidian Parasites
Source: PLoS One. 2015 Apr 8;10(4):e0121254. doi: 10.1371/journal.pone.0121254 (PMC4390322; doi:10.1371/journal.pone.0121254)
Supplement: S2 Table — (PDF) [file pone.0121254.s002.pdf]

Table S2. Host and parasite lineage associations

Host Collection Number	Field Site*	Host Order	Host Family	Host Genus	Host species	Host subspecies	Host common name	Parasite name	Lineage name**	Parasite GenBank Accession Number
467839	1	Anseriformes	Anatidae	*Alopochen*	*aegyptiacus*		Egyptian Goose	0	NA	NA
467841	1	Anseriformes	Anatidae	*Sarkidiornis*	*melanotos*	*melanotos*	Knob-billed Duck	0	NA	NA
467840	1	Anseriformes	Anatidae	*Sarkidiornis*	*melanotos*	*melanotos*	Knob-billed Duck	*Plasmodium* sp.	P_BUL07	KM056642
467918	2	Bucerotiformes	Bucerotidae	*Tockus*	*alboterminatus*	*suahelicus*	Crowned Hornbill	*Leucocytozoon* sp.	L_AFR207	KM056525
467918	2	Bucerotiformes	Bucerotidae	*Tockus*	*alboterminatus*	*suahelicus*	Crowned Hornbill	*Plasmodium* sp.	P_ACCTAC01	KM056621
467919	1	Bucerotiformes	Bucerotidae	*Tockus*	*nasutus*	*caffer*	African Grey Hornbill	*Leucocytozoon* sp.	L_AFR209	KM056527
467886	1	Caprimulgiformes	Caprimulgidae	*Caprimulgus*	*pectoralis*	*fervidus*	Fiery-necked Nightjar	0	NA	NA
467887	1	Caprimulgiformes	Caprimulgidae	*Caprimulgus*	*pectoralis*	*fervidus*	Fiery-necked Nightjar	0	NA	NA
467888	2	Caprimulgiformes	Caprimulgidae	*Caprimulgus*	*poliocephalus*	*guttifer*	Ruwenzori Nightjar	0	NA	NA
467889	2	Caprimulgiformes	Caprimulgidae	*Caprimulgus*	*poliocephalus*	*guttifer*	Ruwenzori Nightjar	0	NA	NA
467890	2	Caprimulgiformes	Caprimulgidae	*Caprimulgus*	*poliocephalus*	*guttifer*	Ruwenzori Nightjar	0	NA	NA
467892	1	Caprimulgiformes	Caprimulgidae	*Scotornis*	*fossii*	*welwitschii*	Square-tailed Nightjar	0	NA	NA
467893	1	Caprimulgiformes	Caprimulgidae	*Scotornis*	*fossii*	*welwitschii*	Square-tailed Nightjar	0	NA	NA
467834	NA	Ciconiiformes	Ardeidae	*Ardea*	*melanocephala*		Black-headed Heron	*Parahaemoproteus* sp.	H_QUERY01	KM056416
467835	NA	Ciconiiformes	Ardeidae	*Bubulcus*	*ibis*		Cattle Egret	Unknown	Coinfection	NA
467836	NA	Ciconiiformes	Ardeidae	*Bubulcus*	*ibis*		Cattle Egret	*Parahaemoproteus* sp.	H_QUERY01	KM056416
467837	NA	Ciconiiformes	Scopidae	*Scopus*	*umbretta*		Hamerkop	*Parahaemoproteus* sp.	H_QUERY01	KM056416
467837	NA	Ciconiiformes	Scopidae	*Scopus*	*umbretta*		Hamerkop	*Leucocytozoon* sp.	L_AFR210	KM056528
467894	1	Coliiformes	Coliidae	*Colius*	*striatus*	*berlepschi*	Speckled Mousebird	0	NA	NA
467895	1	Coliiformes	Coliidae	*Colius*	*striatus*	*berlepschi*	Speckled Mousebird	*Plasmodium* sp.	P_AFR33	KM056595
467865	2	Columbiformes	Columbidae	*Columba*	*arquatrix*		African Olive-Pigeon	Unknown	Coinfection	NA
467866	2	Columbiformes	Columbidae	*Columba*	*arquatrix*		African Olive-Pigeon	Unknown	Coinfection	NA
467865	2	Columbiformes	Columbidae	*Columba*	*arquatrix*		African Olive-Pigeon	*Haemoproteus* sp.	H_AFR112	KM056423
467866	2	Columbiformes	Columbidae	*Columba*	*arquatrix*		African Olive-Pigeon	*Haemoproteus* sp.	H_AFR119	KM056425
467867	2	Columbiformes	Columbidae	*Columba*	*arquatrix*		African Olive-Pigeon	*Parahaemoproteus* sp.	H_AFR120	KM056426
467867	2	Columbiformes	Columbidae	*Columba*	*arquatrix*		African Olive-Pigeon	*Leucocytozoon* sp.	L_AFR178	KM056496
467867	2	Columbiformes	Columbidae	*Columba*	*arquatrix*		African Olive-Pigeon	*Leucocytozoon* sp.	L_AFR220	KM056535
467860	1	Columbiformes	Columbidae	*Streptopelia*	*capicola*	*tropica*	Cape Turtle Dove	0	NA	NA
467861	1	Columbiformes	Columbidae	*Streptopelia*	*capicola*	*tropica*	Cape Turtle Dove	0	NA	NA
467862	2	Columbiformes	Columbidae	*Streptopelia*	*semitorquata*	*semitorquata*	Red-eyed Dove	*Haemoproteus* sp.	H_AFR109	KM056421
467868	1	Columbiformes	Columbidae	*Treron*	*calva*	*schalowi*	African Green-Pigeon	*Haemoproteus* sp.	H_AFR70	KM056461
467870	1	Columbiformes	Columbidae	*Turtur*	*chalcospilos*	*chalcospilos*	Emerald-spotted Wood-Dove	*Haemoproteus* sp.	H_AFR44	KM056450
467871	1	Columbiformes	Columbidae	*Turtur*	*chalcospilos*	*chalcospilos*	Emerald-spotted Wood-Dove	*Haemoproteus* sp.	H_AFR44	KM056450
467869	1	Columbiformes	Columbidae	*Turtur*	*chalcospilos*	*chalcospilos*	Emerald-spotted Wood-Dove	0	NA	NA
467901	1	Coraciiformes	Alcedinidae	*Alcedo*	*cristata*	*cristata*	Malachite Kingfisher	0	NA	NA
467900	1	Coraciiformes	Alcedinidae	*Halcyon*	*senegalensis*	*cyanoleuca*	Woodland Kingfisher	*Parahaemoproteus* sp.	H_AFR151	KM056440
467900	1	Coraciiformes	Alcedinidae	*Halcyon*	*senegalensis*	*cyanoleuca*	Woodland Kingfisher	*Leucocytozoon* sp.	L_AFR208	KM056526
467905	1	Coraciiformes	Alcedinidae	*Ispidina*	*picta*	*natalensis*	African Pygmy-Kingfisher	Unknown	Coinfection	NA
467903	1	Coraciiformes	Alcedinidae	*Ispidina*	*picta*	*natalensis*	African Pygmy-Kingfisher	*Parahaemoproteus* sp.	H_AFR67	KM056460
467902	1	Coraciiformes	Alcedinidae	*Ispidina*	*picta*	*natalensis*	African Pygmy-Kingfisher	*Haemoproteus belopolskyi*	H_SW1	KM056409
467902	1	Coraciiformes	Alcedinidae	*Ispidina*	*picta*	*natalensis*	African Pygmy-Kingfisher	*Plasmodium* sp.	P_AFR46	KM056598
467902	1	Coraciiformes	Alcedinidae	*Ispidina*	*picta*	*natalensis*	African Pygmy-Kingfisher	*Plasmodium* sp.	P_AFR6	KM056605
467917	1	Coraciiformes	Coraciidae	*Eurystomus*	*glaucurus*		Broad-billed Roller	*Leucocytozoon* sp.	L_AFR162	KM056481
467915	1	Coraciiformes	Meropidae	*Merops*	*apiaster*		European Bee-eater	*Parahaemoproteus* sp.	H_QUERY01	KM056416
467915	1	Coraciiformes	Meropidae	*Merops*	*apiaster*		European Bee-eater	*Leucocytozoon* sp.	L_AFR251	KM056562
467914	1	Coraciiformes	Meropidae	*Merops*	*apiaster*		European Bee-eater	0	NA	NA
467909	1	Coraciiformes	Meropidae	*Merops*	*pusillus*	*meridionalis*	Little Bee-eater	*Parahaemoproteus* sp.	H_AFR35	KM056447
467911	1	Coraciiformes	Meropidae	*Merops*	*pusillus*	*meridionalis*	Little Bee-eater	*Parahaemoproteus* sp.	H_AFR35	KM056447
467910	1	Coraciiformes	Meropidae	*Merops*	*pusillus*	*meridionalis*	Little Bee-eater	0	NA	NA

467912	1	Coraciiformes	Meropidae	*Merops*	*pusillus*	*meridionalis*	Little Bee-eater	0	NA	NA
467913	1	Coraciiformes	Meropidae	*Merops*	*pusillus*	*meridionalis*	Little Bee-eater	0	NA	NA
467909	1	Coraciiformes	Meropidae	*Merops*	*pusillus*	*meridionalis*	Little Bee-eater	*Plasmodium* sp.	P_BUL07	KM056642
467877	1	Cuculiformes	Cuculidae	*Centropus*	*superciliosus*	*loandae*	White-browed Coucal	*Parahaemoproteus* sp.	H_AFR61	KM056457
467876	1	Cuculiformes	Cuculidae	*Centropus*	*superciliosus*	*loandae*	White-browed Coucal	*Plasmodium* sp.	P_BUL07	KM056642
467882	2	Cuculiformes	Cuculidae	*Chrysococcyx*	*klaas*	*klaas*	Klaas's Cuckoo	*Parahaemoproteus* sp.	H_AFR8	KM056468
467878	1	Cuculiformes	Cuculidae	*Cuculus*	*canorus*	*gularis*	African Cuckoo	0	NA	NA
467842	1	Falconiformes	Accipitridae	*Aquila*	*wahlbergi*		Wahlberg's Eagle	*Plasmodium* sp.	P_RTSR1	KM056623
467843	1	Falconiformes	Accipitridae	*Milvus*	*migrans*	*parasitus*	Yellow-billed Kite	*Parahaemoproteus* sp.	H_AFR48	KM056451
467850	1	Galliformes	Numididae	*Numida*	*meleagris*	*mitrata*	Helmeted Guineafowl	*Parahaemoproteus* sp.	H_AFR50	KM056452
467848	2	Galliformes	Phasianidae	*Coturnix*	*coturnix*	*africana*	Common Quail	0	NA	NA
467844	2	Galliformes	Phasianidae	*Francolinus*	*levaillantii*	*crawshayi*	Red-winged Francolin	*Leucocytozoon spp.*	Coinfection	*Leucocytozoon* spp.
467845	2	Galliformes	Phasianidae	*Francolinus*	*levaillantii*	*crawshayi*	Red-winged Francolin	*Leucocytozoon* sp.	L_AFR245	KM056558
467845	2	Galliformes	Phasianidae	*Francolinus*	*levaillantii*	*crawshayi*	Red-winged Francolin	*Plasmodium* sp.	P_BUL07	KM056642
468628	2	Galliformes	Phasianidae	*Gallus*	*gallus*		Chicken	*Parahaemoproteus* sp.	H_QUERY01	KM056416
468628	2	Galliformes	Phasianidae	*Gallus*	*gallus*		Chicken	*Leucocytozoon schoutedeni*	L_GALLUS06	KM056646
467880	1	Galliformes	Phasianidae	*Gallus*	*gallus*		Chicken	0	NA	NA
468623	1	Galliformes	Phasianidae	*Gallus*	*gallus*		Chicken	0	NA	NA
467853	2	Gruiformes	Rallidae	*Sarothrura*	*rufa*	*rufa*	Red-chested Flufftail	*Plasmodium* sp.	P_AFR118	KM056573
467853	2	Gruiformes	Rallidae	*Sarothrura*	*rufa*	*rufa*	Red-chested Flufftail	*Plasmodium* sp.	P_PSEGRI01	KM056637
467874	2	Musophagiformes	Musophagidae	*Tauraco*	*corythaix*	*schalowi*	Schalow's Turaco	Unknown	Coinfection	NA
467874	2	Musophagiformes	Musophagidae	*Tauraco*	*corythaix*	*schalowi*	Schalow's Turaco	*Parahaemoproteus* sp.	H_AFR59	KM056456
467947	1	Passeriformes	Alaudidae	*Mirafra*	*rufocinnamomea*	*fischeri*	Flappet Lark	0	NA	NA
467946	1	Passeriformes	Alaudidae	*Mirafra*	*rufocinnamomea*	*fischeri*	Flappet Lark	*Plasmodium* sp.	P_ACCTAC01	KM056621
467945	1	Passeriformes	Alaudidae	*Mirafra*	*rufocinnamomea*	*fischeri*	Flappet Lark	*Plasmodium* sp.	P_AFR69	KM056609
468254	1	Passeriformes	Cisticolidae	*Apalis*	*thoracica*	*youngi*	Bar-throated Apalis	*Leucocytozoon* sp.	L_AFR181	KM056499
468254	1	Passeriformes	Cisticolidae	*Apalis*	*thoracica*	*youngi*	Bar-throated Apalis	*Leucocytozoon* sp.	L_AFR182	KM056500
468251	2	Passeriformes	Cisticolidae	*Apalis*	*thoracica*	*youngi*	Bar-throated Apalis	0	NA	NA
468253	2	Passeriformes	Cisticolidae	*Apalis*	*thoracica*	*youngi*	Bar-throated Apalis	0	NA	NA
468260	1	Passeriformes	Cisticolidae	*Calamonastes*	*stierlingi*	*irwini*	Stierling's Wren-Warbler	*Leucocytozoon* sp.	L_AFR218	KM056533
468258	1	Passeriformes	Cisticolidae	*Calamonastes*	*stierlingi*	*irwini*	Stierling's Wren-Warbler	*Plasmodium* sp.	P_RFF1	KM056632
468260	1	Passeriformes	Cisticolidae	*Calamonastes*	*stierlingi*	*irwini*	Stierling's Wren-Warbler	*Plasmodium* sp.	P_WW4	KM056626
468204	1	Passeriformes	Cisticolidae	*Cisticola*	*brachyptera*	*isabellina*	Short-winged Cisticola	*Leucocytozoon* sp.	L_AFR211	KM056529
468204	1	Passeriformes	Cisticolidae	*Cisticola*	*brachyptera*	*isabellina*	Short-winged Cisticola	*Plasmodium* sp.	P_AFR5	KM056601
468205	1	Passeriformes	Cisticolidae	*Cisticola*	*brachyptera*	*isabellina*	Short-winged Cisticola	*Plasmodium* sp.	P_RFF1	KM056632
468232	1	Passeriformes	Cisticolidae	*Cisticola*	*erythrops*	*nyasa*	Red-faced Cisticola	Unknown	Coinfection	NA
468236	2	Passeriformes	Cisticolidae	*Cisticola*	*erythrops*	*nyasa*	Red-faced Cisticola	Unknown	Coinfection	NA
468234	1	Passeriformes	Cisticolidae	*Cisticola*	*erythrops*	*nyasa*	Red-faced Cisticola	0	NA	NA
468235	1	Passeriformes	Cisticolidae	*Cisticola*	*erythrops*	*nyasa*	Red-faced Cisticola	0	NA	NA
468225	1	Passeriformes	Cisticolidae	*Cisticola*	*erythrops*	*nyasa*	Red-faced Cisticola	*Plasmodium* sp.	P_AFR13	KM056579
468227	1	Passeriformes	Cisticolidae	*Cisticola*	*erythrops*	*nyasa*	Red-faced Cisticola	*Plasmodium* sp.	P_AFR26	KM056592
468226	1	Passeriformes	Cisticolidae	*Cisticola*	*erythrops*	*nyasa*	Red-faced Cisticola	*Plasmodium* sp.	P_PSEGRI01	KM056637
468233	1	Passeriformes	Cisticolidae	*Cisticola*	*erythrops*	*nyasa*	Red-faced Cisticola	*Plasmodium* sp.	P_PSEGRI01	KM056637
468228	1	Passeriformes	Cisticolidae	*Cisticola*	*erythrops*	*nyasa*	Red-faced Cisticola	*Plasmodium* sp.	P_RFF1	KM056632
468237	1	Passeriformes	Cisticolidae	*Cisticola*	*fulvicapilla*	*muelleri*	Piping Cisticola	*Haemoproteus lanii*	H_RBS4	KM056411
468220	1	Passeriformes	Cisticolidae	*Cisticola*	*natalensis*	*matengorum*	Croaking Cisticola	*Parahaemoproteus* sp.	H_AFR34	KM056446
468216	1	Passeriformes	Cisticolidae	*Cisticola*	*natalensis*	*matengorum*	Croaking Cisticola	*Plasmodium* sp.	P_AFR13	KM056579
468220	1	Passeriformes	Cisticolidae	*Cisticola*	*natalensis*	*matengorum*	Croaking Cisticola	*Plasmodium* sp.	P_AFR13	KM056579
468221	1	Passeriformes	Cisticolidae	*Cisticola*	*natalensis*	*matengorum*	Croaking Cisticola	*Plasmodium* sp.	P_AFR37	KM056596
468224	1	Passeriformes	Cisticolidae	*Cisticola*	*natalensis*	*matengorum*	Croaking Cisticola	*Plasmodium* sp.	P_LINOLI01	KM056629
468215	1	Passeriformes	Cisticolidae	*Cisticola*	*natalensis*	*matengorum*	Croaking Cisticola	*Plasmodium* sp.	P_RFF1	KM056632
468192	2	Passeriformes	Cisticolidae	*Cisticola*	*nigriloris*		Black-lored Cisticola	*Leucocytozoon spp.*	Coinfection	*Leucocytozoon* spp.
468298	2	Passeriformes	Cisticolidae	*Cisticola*	*nigriloris*		Black-lored Cisticola	Unknown	Coinfection	NA

468190	2	Passeriformes	Cisticolidae	*Cisticola*	*nigriloris*		Black-lored Cisticola	*Haemoproteus payevski*	H_RW1	KM056406
468193	2	Passeriformes	Cisticolidae	*Cisticola*	*nigriloris*		Black-lored Cisticola	*Leucocytozoon* sp.	L_AFR173	KM056491
468195	2	Passeriformes	Cisticolidae	*Cisticola*	*nigriloris*		Black-lored Cisticola	*Leucocytozoon* sp.	L_AFR211	KM056529
468188	2	Passeriformes	Cisticolidae	*Cisticola*	*nigriloris*		Black-lored Cisticola	0	NA	NA
468189	2	Passeriformes	Cisticolidae	*Cisticola*	*nigriloris*		Black-lored Cisticola	0	NA	NA
468191	2	Passeriformes	Cisticolidae	*Cisticola*	*nigriloris*		Black-lored Cisticola	*Plasmodium* sp.	P_AFR105	KM056566
468192	2	Passeriformes	Cisticolidae	*Cisticola*	*nigriloris*		Black-lored Cisticola	*Plasmodium* sp.	P_AFR106	KM056567
468194	2	Passeriformes	Cisticolidae	*Cisticola*	*nigriloris*		Black-lored Cisticola	*Plasmodium* sp.	P_AFR107	KM056568
468190	2	Passeriformes	Cisticolidae	*Cisticola*	*nigriloris*		Black-lored Cisticola	*Plasmodium* sp.	P_PSEGRI01	KM056637
468201	2	Passeriformes	Cisticolidae	*Cisticola*	*njombe*		Churring Cisticola	*Parahaemoproteus* sp.	H_AFR103	KM056420
468202	2	Passeriformes	Cisticolidae	*Cisticola*	*njombe*		Churring Cisticola	*Leucocytozoon* sp.	L_AFR211	KM056529
468196	2	Passeriformes	Cisticolidae	*Cisticola*	*njombe*		Churring Cisticola	0	NA	NA
468200	2	Passeriformes	Cisticolidae	*Cisticola*	*njombe*		Churring Cisticola	0	NA	NA
468197	2	Passeriformes	Cisticolidae	*Cisticola*	*njombe*		Churring Cisticola	*Plasmodium* sp.	P_PSEGRI01	KM056637
468202	2	Passeriformes	Cisticolidae	*Cisticola*	*njombe*		Churring Cisticola	*Plasmodium* sp.	P_PSEGRI01	KM056637
468210	1	Passeriformes	Cisticolidae	*Cisticola*	*rufilata*	*ansorgei*	Grey Cisticola	*Plasmodium* sp.	P_AFR13	KM056579
468207	1	Passeriformes	Cisticolidae	*Cisticola*	*rufilata*	*ansorgei*	Grey Cisticola	*Plasmodium* sp.	P_RFF1	KM056632
468208	1	Passeriformes	Cisticolidae	*Cisticola*	*rufilata*	*ansorgei*	Grey Cisticola	*Plasmodium* sp.	P_RFF1	KM056632
468209	1	Passeriformes	Cisticolidae	*Cisticola*	*rufilata*	*ansorgei*	Grey Cisticola	*Plasmodium* sp.	P_RFF1	KM056632
468214	2	Passeriformes	Cisticolidae	*Cisticola*	*woosnami*	*lufira*	Trilling Cisticola	Unknown	Coinfection	NA
468212	2	Passeriformes	Cisticolidae	*Cisticola*	*woosnami*	*lufira*	Trilling Cisticola	0	NA	NA
468213	2	Passeriformes	Cisticolidae	*Cisticola*	*woosnami*	*lufira*	Trilling Cisticola	*Plasmodium* sp.	P_AFR143	KM056585
468211	2	Passeriformes	Cisticolidae	*Cisticola*	*woosnami*	*lufira*	Trilling Cisticola	*Plasmodium* sp.	P_WW4	KM056626
468176	1	Passeriformes	Cisticolidae	*Prinia*	*erythroptera*	*rhodoptera*	Red-winged Warbler	*Leucocytozoon* sp.	L_AFR218	KM056533
468176	1	Passeriformes	Cisticolidae	*Prinia*	*erythroptera*	*rhodoptera*	Red-winged Warbler	*Plasmodium* sp.	P_SYBOR11	KM056638
468625	2	Passeriformes	Corvidae	*Corvus*	*albicollis*		White-necked Raven	*Parahaemoproteus* sp.	H_AFR2	KM056443
468627	2	Passeriformes	Corvidae	*Corvus*	*albicollis*		White-necked Raven	*Leucocytozoon* sp.	L_WW6	KM056645
468630	2	Passeriformes	Corvidae	*Corvus*	*albicollis*		White-necked Raven	0	NA	NA
468625	2	Passeriformes	Corvidae	*Corvus*	*albicollis*		White-necked Raven	*Plasmodium* sp.	P_BUL07	KM056642
468615	1	Passeriformes	Dicruridae	*Dicrurus*	*adsimilis*	*adsimilis*	Fork-tailed Drongo	Unknown	Coinfection	NA
468618	1	Passeriformes	Dicruridae	*Dicrurus*	*adsimilis*	*adsimilis*	Fork-tailed Drongo	Unknown	Coinfection	NA
468617	1	Passeriformes	Dicruridae	*Dicrurus*	*adsimilis*	*adsimilis*	Fork-tailed Drongo	*Haemoproteus lanii*	H_RBS4	KM056411
468616	1	Passeriformes	Dicruridae	*Dicrurus*	*adsimilis*	*adsimilis*	Fork-tailed Drongo	0	NA	NA
468614	1	Passeriformes	Dicruridae	*Dicrurus*	*adsimilis*	*adsimilis*	Fork-tailed Drongo	*Plasmodium* sp.	P_AFR10	KM056563
468614	1	Passeriformes	Dicruridae	*Dicrurus*	*adsimilis*	*adsimilis*	Fork-tailed Drongo	*Plasmodium* sp.	P_BUL07	KM056642
468355	1	Passeriformes	Emberizidae	*Emberiza*	*cabanisi*	*orientalis*	Cabanis's Bunting	*Parahaemoproteus* sp.	H_AFR90	KM056472
468354	1	Passeriformes	Emberizidae	*Emberiza*	*flaviventris*	*kalaharica*	African Golden-breasted Bunting	*Plasmodium* sp.	P_AFR47	KM056599
468354	1	Passeriformes	Emberizidae	*Emberiza*	*flaviventris*	*kalaharica*	kalaharica	*Plasmodium* sp.	P_AFR6	KM056605
468458	1	Passeriformes	Estrildidae	*Amandava*	*subflava*	*clarkei*	Zebra Waxbill	0	NA	NA
468482	1	Passeriformes	Estrildidae	*Amandava*	*subflava*	*clarkei*	Zebra Waxbill	0	NA	NA
468460	1	Passeriformes	Estrildidae	*Amandava*	*subflava*	*clarkei*	Zebra Waxbill	0	NA	NA
468461	1	Passeriformes	Estrildidae	*Amandava*	*subflava*	*clarkei*	Zebra Waxbill	0	NA	NA
468481	1	Passeriformes	Estrildidae	*Amandava*	*subflava*	*clarkei*	Zebra Waxbill	*Plasmodium* sp.	P_AFR40	KM056597
468459	1	Passeriformes	Estrildidae	*Amandava*	*subflava*	*clarkei*	Zebra Waxbill	*Plasmodium* sp.	P_AFR58	KM056604
468459	1	Passeriformes	Estrildidae	*Amandava*	*subflava*	*clarkei*	Zebra Waxbill	*Plasmodium* sp.	P_BUL07	KM056642
468427	2	Passeriformes	Estrildidae	*Cryptospiza*	*reichenovii*	*australis*	Red-faced Crimsonwing	Unknown	Coinfection	NA
468436	2	Passeriformes	Estrildidae	*Cryptospiza*	*reichenovii*	*australis*	Red-faced Crimsonwing	*Parahaemoproteus* sp.	H_AFR25	KM056444
468428	2	Passeriformes	Estrildidae	*Cryptospiza*	*reichenovii*	*australis*	Red-faced Crimsonwing	*Parahaemoproteus* sp.	H_AFR35	KM056447
468433	2	Passeriformes	Estrildidae	*Cryptospiza*	*reichenovii*	*australis*	Red-faced Crimsonwing	*Leucocytozoon* sp.	L_AFR192	KM056510
468425	2	Passeriformes	Estrildidae	*Cryptospiza*	*reichenovii*	*australis*	Red-faced Crimsonwing	*Leucocytozoon* sp.	L_AFR214	KM056531
468428	2	Passeriformes	Estrildidae	*Cryptospiza*	*reichenovii*	*australis*	Red-faced Crimsonwing	*Leucocytozoon* sp.	L_AFR214	KM056531
468438	2	Passeriformes	Estrildidae	*Cryptospiza*	*reichenovii*	*australis*	Red-faced Crimsonwing	*Leucocytozoon* sp.	L_AFR214	KM056531
468435	2	Passeriformes	Estrildidae	*Cryptospiza*	*reichenovii*	*australis*	Red-faced Crimsonwing	*Leucocytozoon* sp.	L_AFR214	KM056531

468436	2	Passeriformes	Estrildidae	*Cryptospiza*	*reichenovii*	*australis*	Red-faced Crimsonwing	*Leucocytozoon* sp.	L_AFR214	KM056531
468438	2	Passeriformes	Estrildidae	*Cryptospiza*	*reichenovii*	*australis*	Red-faced Crimsonwing	*Leucocytozoon* sp.	L_AFR222	KM056536
468432	2	Passeriformes	Estrildidae	*Cryptospiza*	*reichenovii*	*australis*	Red-faced Crimsonwing	*Leucocytozoon* sp.	L_AFR223	KM056537
468434	2	Passeriformes	Estrildidae	*Cryptospiza*	*reichenovii*	*australis*	Red-faced Crimsonwing	*Leucocytozoon* sp.	L_AFR223	KM056537
468431	2	Passeriformes	Estrildidae	*Cryptospiza*	*reichenovii*	*australis*	Red-faced Crimsonwing	0	NA	NA
468434	2	Passeriformes	Estrildidae	*Cryptospiza*	*reichenovii*	*australis*	Red-faced Crimsonwing	*Plasmodium* sp.	P_AFR132	KM056581
468432	2	Passeriformes	Estrildidae	*Cryptospiza*	*reichenovii*	*australis*	Red-faced Crimsonwing	*Plasmodium* sp.	P_PSEGRI01	KM056637
468457	2	Passeriformes	Estrildidae	*Estrilda*	*astrild*	*cavendishi*	Common Waxbill	Unknown	Coinfection	NA
468455	2	Passeriformes	Estrildidae	*Estrilda*	*astrild*	*cavendishi*	Common Waxbill	0	NA	NA
468456	2	Passeriformes	Estrildidae	*Estrilda*	*astrild*	*cavendishi*	Common Waxbill	0	NA	NA
468475	1	Passeriformes	Estrildidae	*Estrilda*	*astrild*	*cavendishi*	Common Waxbill	*Plasmodium* sp.	P_BUL07	KM056642
468465	2	Passeriformes	Estrildidae	*Estrilda*	*melanotis*	*stuartirwini*	Yellow-bellied Waxbill	*Leucocytozoon* sp.	L_AFR177	KM056495
468463	2	Passeriformes	Estrildidae	*Estrilda*	*melanotis*	*stuartirwini*	Yellow-bellied Waxbill	*Leucocytozoon* sp.	L_AFR214	KM056531
468462	2	Passeriformes	Estrildidae	*Estrilda*	*melanotis*	*stuartirwini*	Yellow-bellied Waxbill	0	NA	NA
468464	2	Passeriformes	Estrildidae	*Estrilda*	*melanotis*	*stuartirwini*	Yellow-bellied Waxbill	*Plasmodium* sp.	P_AFR22	KM056590
468441	2	Passeriformes	Estrildidae	*Hypargos*	*niveoguttatus*	*macrospilotus*	Peters's Twinspot	*Parahaemoproteus* sp.	H_AFR59	KM056456
468439	2	Passeriformes	Estrildidae	*Hypargos*	*niveoguttatus*	*macrospilotus*	Peters's Twinspot	*Leucocytozoon* sp.	L_AFR212	KM056530
468440	2	Passeriformes	Estrildidae	*Hypargos*	*niveoguttatus*	*macrospilotus*	Peters's Twinspot	*Plasmodium* sp.	P_BUL07	KM056642
468442	1	Passeriformes	Estrildidae	*Lagonosticta*	*rubricata*	*haematocephala*	African Firefinch	*Leucocytozoon* sp.	L_AFR211	KM056529
468444	1	Passeriformes	Estrildidae	*Lagonosticta*	*rubricata*	*haematocephala*	African Firefinch	0	NA	NA
468443	1	Passeriformes	Estrildidae	*Lagonosticta*	*rubricata*	*haematocephala*	African Firefinch	*Plasmodium* sp.	P_RFF1	KM056632
479646	1	Passeriformes	Estrildidae	*Lagonosticta*	*rubricata*		African Firefinch	*Leucocytozoon* sp.	L_AFR157	KM056477
479646	1	Passeriformes	Estrildidae	*Lagonosticta*	*rubricata*		African Firefinch	*Leucocytozoon* sp.	L_AFR235	KM056549
479646	1	Passeriformes	Estrildidae	*Lagonosticta*	*rubricata*		African Firefinch	*Plasmodium* sp.	P_AFR31	KM056594
468468	1	Passeriformes	Estrildidae	*Lonchura*	*cucullata*	*scutata*	Bronze Mannikin	*Parahaemoproteus* sp.	H_AFR25	KM056444
468472	1	Passeriformes	Estrildidae	*Lonchura*	*cucullata*	*scutata*	Bronze Mannikin	*Parahaemoproteus* sp.	H_AFR57	KM056455
468470	1	Passeriformes	Estrildidae	*Lonchura*	*cucullata*	*scutata*	Bronze Mannikin	0	NA	NA
468466	1	Passeriformes	Estrildidae	*Lonchura*	*cucullata*	*scutata*	Bronze Mannikin	*Plasmodium* sp.	P_BUL07	KM056642
468417	1	Passeriformes	Estrildidae	*Pytilia*	*afra*		Orange-winged Pytilia	*Leucocytozoon* sp.	L_AFR214	KM056531
468414	1	Passeriformes	Estrildidae	*Pytilia*	*afra*		Orange-winged Pytilia	0	NA	NA
468415	1	Passeriformes	Estrildidae	*Pytilia*	*afra*		Orange-winged Pytilia	0	NA	NA
468420	1	Passeriformes	Estrildidae	*Pytilia*	*afra*		Orange-winged Pytilia	*Plasmodium* sp.	P_AFR13	KM056579
468421	1	Passeriformes	Estrildidae	*Pytilia*	*afra*		Orange-winged Pytilia	*Plasmodium* sp.	P_AFR91	KM056615
468421	1	Passeriformes	Estrildidae	*Pytilia*	*afra*		Orange-winged Pytilia	*Plasmodium* sp.	P_AFR92	KM056616
468414	1	Passeriformes	Estrildidae	*Pytilia*	*afra*		Orange-winged Pytilia	*Plasmodium* sp.	P_RFF1	KM056632
468417	1	Passeriformes	Estrildidae	*Pytilia*	*afra*		Orange-winged Pytilia	*Plasmodium* sp.	P_RFF1	KM056632
468420	1	Passeriformes	Estrildidae	*Pytilia*	*afra*		Orange-winged Pytilia	*Plasmodium* sp.	P_RFF1	KM056632
468422	1	Passeriformes	Estrildidae	*Pytilia*	*melba*	*melba*	Green-winged Pytilia	*Plasmodium* sp.	P_AFR10	KM056563
468422	1	Passeriformes	Estrildidae	*Pytilia*	*melba*	*melba*	Green-winged Pytilia	*Plasmodium* sp.	P_COLL7	KM056625
468447	1	Passeriformes	Estrildidae	*Uraeginthus*	*angolensis*	*niassensis*	Southern Cordonbleu	0	NA	NA
468624	1	Passeriformes	Estrildidae	*Uraeginthus*	*angolensis*	*niassensis*	Southern Cordonbleu	*Plasmodium* sp.	P_GRW09	KM056631
468448	1	Passeriformes	Estrildidae	*Uraeginthus*	*angolensis*	*niassensis*	Southern Cordonbleu	*Plasmodium* sp.	P_RFF1	KM056632
467943	2	Passeriformes	Eurylaimidae	*Smithornis*	*capensis*	*albigularis*	African Broadbill	*Leucocytozoon* sp.	L_AFR175	KM056493
468382	2	Passeriformes	Fringillidae	*Serinus*	*canicollis*	*sassii*	Cape Canary	*Leucocytozoon* sp.	L_ZOABY02	KM056650
468389	2	Passeriformes	Fringillidae	*Serinus*	*canicollis*	*sassii*	Cape Canary	*Leucocytozoon* sp.	L_ZOABY02	KM056650
468383	2	Passeriformes	Fringillidae	*Serinus*	*canicollis*	*sassii*	Cape Canary	*Plasmodium* sp.	P_AFR101	KM056564
468356	2	Passeriformes	Fringillidae	*Serinus*	*citrinelloides*	*hypostictus*	African Citril	*Leucocytozoon* sp.	L_AFR244	KM056557
468376	1	Passeriformes	Fringillidae	*Serinus*	*mozambicus*	*mozambicus*	Yellow-fronted Canary	*Parahaemoproteus* sp.	H_AFR51	KM056453
468378	1	Passeriformes	Fringillidae	*Serinus*	*mozambicus*	*mozambicus*	Yellow-fronted Canary	*Parahaemoproteus* sp.	H_AFR63	KM056459
468374	1	Passeriformes	Fringillidae	*Serinus*	*mozambicus*	*mozambicus*	Yellow-fronted Canary	*Parahaemoproteus* sp.	H_AFR72	KM056463
468374	1	Passeriformes	Fringillidae	*Serinus*	*mozambicus*	*mozambicus*	Yellow-fronted Canary	*Parahaemoproteus* sp.	H_AFR73	KM056464
468375	1	Passeriformes	Fringillidae	*Serinus*	*mozambicus*	*mozambicus*	Yellow-fronted Canary	*Parahaemoproteus* sp.	H_AFR75	KM056465
468375	1	Passeriformes	Fringillidae	*Serinus*	*mozambicus*	*mozambicus*	Yellow-fronted Canary	*Leucocytozoon* sp.	L_AFR161	KM056480

468374	1	Passeriformes	Fringillidae	*Serinus*	*mozambicus*	*mozambicus*	Yellow-fronted Canary	*Leucocytozoon* sp.	L_REB7	KM056647
468377	1	Passeriformes	Fringillidae	*Serinus*	*mozambicus*	*mozambicus*	Yellow-fronted Canary	*Plasmodium* sp.	P_AFR83	KM056612
468377	1	Passeriformes	Fringillidae	*Serinus*	*mozambicus*	*mozambicus*	Yellow-fronted Canary	*Plasmodium* sp.	P_GRW09	KM056631
468375	1	Passeriformes	Fringillidae	*Serinus*	*mozambicus*	*mozambicus*	Yellow-fronted Canary	*Plasmodium* sp.	P_WW3	NA
468358	2	Passeriformes	Fringillidae	*Serinus*	*striolatus*	*whytii*	Yellow-browed Seedeater	*Parahaemoproteus* sp.	H_PYERY01	KM056418
468362	2	Passeriformes	Fringillidae	*Serinus*	*striolatus*	*whytii*	Yellow-browed Seedeater	*Leucocytozoon* sp.	L_RECOB3	KM056648
468364	2	Passeriformes	Fringillidae	*Serinus*	*striolatus*	*whytii*	Yellow-browed Seedeater	*Leucocytozoon* sp.	L_RECOB3	KM056648
468359	2	Passeriformes	Fringillidae	*Serinus*	*striolatus*	*whytii*	Yellow-browed Seedeater	*Leucocytozoon* sp.	L_ZOABY02	KM056650
468361	2	Passeriformes	Fringillidae	*Serinus*	*striolatus*	*whytii*	Yellow-browed Seedeater	*Leucocytozoon* sp.	L_ZOABY02	KM056650
468365	2	Passeriformes	Fringillidae	*Serinus*	*striolatus*	*whytii*	Yellow-browed Seedeater	0	NA	NA
468366	2	Passeriformes	Fringillidae	*Serinus*	*striolatus*	*whytii*	Yellow-browed Seedeater	0	NA	NA
468367	2	Passeriformes	Fringillidae	*Serinus*	*striolatus*	*whytii*	Yellow-browed Seedeater	0	NA	NA
468370	2	Passeriformes	Fringillidae	*Serinus*	*striolatus*	*whytii*	Yellow-browed Seedeater	0	NA	NA
468368	2	Passeriformes	Fringillidae	*Serinus*	*striolatus*	*whytii*	Yellow-browed Seedeater	*Plasmodium* sp.	P_AFR28	KM056593
468362	2	Passeriformes	Fringillidae	*Serinus*	*striolatus*	*whytii*	Yellow-browed Seedeater	*Plasmodium* sp.	P_AFR99	KM056620
468361	2	Passeriformes	Fringillidae	*Serinus*	*striolatus*	*whytii*	Yellow-browed Seedeater	*Plasmodium* sp.	P_BUL07	KM056642
468369	2	Passeriformes	Fringillidae	*Serinus*	*striolatus*	*whytii*	Yellow-browed Seedeater	*Plasmodium* sp.	P_MALNI02	KM056641
468359	2	Passeriformes	Fringillidae	*Serinus*	*striolatus*	*whytii*	Yellow-browed Seedeater	*Plasmodium* sp.	P_PSEGRI01	KM056637
467954	1	Passeriformes	Hirundinidae	*Delichon*	*urbica*	*urbica*	House Martin	*Leucocytozoon* sp.	L_AFR167	KM056486
467952	2	Passeriformes	Hirundinidae	*Hirundo*	*angolensis*	*angolensis*	Angola Swallow	*Parahaemoproteus* sp.	H_AFR103	KM056420
467953	2	Passeriformes	Hirundinidae	*Hirundo*	*angolensis*	*angolensis*	Angola Swallow	*Plasmodium* sp.	P_BUL07	KM056642
467949	2	Passeriformes	Hirundinidae	*Psalidoprocne*	*albiceps*	*albiceps*	White-headed Sawwing	*Plasmodium* sp.	P_GRW09	KM056631
467950	2	Passeriformes	Hirundinidae	*Psalidoprocne*	*albiceps*	*albiceps*	White-headed Sawwing	*Plasmodium* sp.	P_GRW09	KM056631
468040	1	Passeriformes	Laniidae	*Lanius*	*collaris*	*capelli*	Common Fiscal	Unknown	Coinfection	NA
468038	1	Passeriformes	Malaconotidae	*Laniarius*	*ferrugineus*	*mossambicus*	Tropical Boubou	*Leucocytozoon spp.*	Coinfection	*Leucocytozoon* spp.
468030	2	Passeriformes	Malaconotidae	*Laniarius*	*ferrugineus*	*mossambicus*	Tropical Boubou	*Leucocytozoon* sp.	L_AFR203	KM056521
468027	1	Passeriformes	Malaconotidae	*Laniarius*	*ferrugineus*	*mossambicus*	Tropical Boubou	*Leucocytozoon* sp.	L_AFR218	KM056533
468030	2	Passeriformes	Malaconotidae	*Laniarius*	*ferrugineus*	*mossambicus*	Tropical Boubou	*Leucocytozoon* sp.	L_AFR233	KM056547
468030	2	Passeriformes	Malaconotidae	*Laniarius*	*ferrugineus*	*mossambicus*	Tropical Boubou	*Plasmodium* sp.	P_AFR6	KM056605
468026	1	Passeriformes	Malaconotidae	*Laniarius*	*ferrugineus*	*mossambicus*	Tropical Boubou	*Plasmodium* sp.	P_AFR6	KM056605
468037	1	Passeriformes	Malaconotidae	*Laniarius*	*ferrugineus*	*mossambicus*	Tropical Boubou	*Plasmodium* sp.	P_AFR6	KM056605
468029	1	Passeriformes	Malaconotidae	*Laniarius*	*ferrugineus*	*mossambicus*	Tropical Boubou	*Plasmodium* sp.	P_AFR6	KM056605
468027	1	Passeriformes	Malaconotidae	*Laniarius*	*ferrugineus*	*mossambicus*	Tropical Boubou	*Plasmodium* sp.	P_AFR9	KM056614
468037	1	Passeriformes	Malaconotidae	*Laniarius*	*ferrugineus*	*mossambicus*	Tropical Boubou	*Plasmodium* sp.	P_AFR9	KM056614
468038	1	Passeriformes	Malaconotidae	*Laniarius*	*ferrugineus*	*mossambicus*	Tropical Boubou	*Plasmodium* sp.	P_BUL07	KM056642
468031	2	Passeriformes	Malaconotidae	*Laniarius*	*fulleborni*	*fulleborni*	Fuelleborn's Boubou	*Leucocytozoon* sp.	L_AFR219	KM056534
468033	2	Passeriformes	Malaconotidae	*Laniarius*	*fulleborni*	*fulleborni*	Fuelleborn's Boubou	*Leucocytozoon* sp.	L_AFR219	KM056534
468032	2	Passeriformes	Malaconotidae	*Laniarius*	*fulleborni*	*fulleborni*	Fuelleborn's Boubou	*Leucocytozoon* sp.	L_AFR242	KM056555
468031	2	Passeriformes	Malaconotidae	*Laniarius*	*fulleborni*	*fulleborni*	Fuelleborn's Boubou	*Plasmodium* sp.	P_AFR6	KM056605
468032	2	Passeriformes	Malaconotidae	*Laniarius*	*fulleborni*	*fulleborni*	Fuelleborn's Boubou	*Plasmodium* sp.	P_BUL07	KM056642
468036	1	Passeriformes	Malaconotidae	*Malaconotus*	*blanchoti*	*hypopyrrhus*	Grey-headed Bush-Shrike	*Plasmodium* sp.	P_AFR22	KM056590
468025	2	Passeriformes	Malaconotidae	*Tchagra*	*australis*	*congener*	Brown-crowned Tchagra	*Leucocytozoon* sp.	L_AFR222	KM056536
468025	2	Passeriformes	Malaconotidae	*Tchagra*	*australis*	*congener*	Brown-crowned Tchagra	*Plasmodium* sp.	P_AFR145	KM056586
468025	2	Passeriformes	Malaconotidae	*Tchagra*	*australis*	*congener*	Brown-crowned Tchagra	*Plasmodium* sp.	P_AFR146	KM056587
468021	1	Passeriformes	Malaconotidae	*Tchagra*	*minuta*	*anchietae*	Anchieta's Tchagra	*Leucocytozoon spp.*	Coinfection	*Leucocytozoon* spp.
468022	1	Passeriformes	Malaconotidae	*Tchagra*	*senegala*	*armena*	Black-crowned Tchagra	0	NA	NA
468023	1	Passeriformes	Malaconotidae	*Tchagra*	*senegala*	*armena*	Black-crowned Tchagra	0	NA	NA
468289	1	Passeriformes	Monarchidae	*Terpsiphone*	*viridis*	*plumbeiceps*	African Paradise-flycatcher	*Parahaemoproteus* sp.	H_TERUF01	KM056415
468293	2	Passeriformes	Monarchidae	*Trochocercus*	*albonotatus*	*albonotatus*	White-tailed Crested-Flycatcher	*Leucocytozoon* sp.	L_AFR183	KM056501
468294	2	Passeriformes	Monarchidae	*Trochocercus*	*albonotatus*	*albonotatus*	White-tailed Crested-Flycatcher	*Leucocytozoon* sp.	L_AFR234	KM056548
468292	2	Passeriformes	Monarchidae	*Trochocercus*	*albonotatus*	*albonotatus*	White-tailed Crested-Flycatcher	*Leucocytozoon* sp.	L_WW6	KM056645
468296	2	Passeriformes	Monarchidae	*Trochocercus*	*albonotatus*	*albonotatus*	White-tailed Crested-Flycatcher	*Plasmodium* sp.	P_AFR140	KM056584
468296	2	Passeriformes	Monarchidae	*Trochocercus*	*albonotatus*	*albonotatus*	White-tailed Crested-Flycatcher	*Plasmodium* sp.	P_BUL07	KM056642

467960	2	Passeriformes	Motacillidae	*Anthus*	*novaeseelandiae*	*lichenya*	African Pipit	0	NA	NA
467959	2	Passeriformes	Motacillidae	*Anthus*	*novaeseelandiae*	*lichenya*	African Pipit	*Plasmodium* sp.	P_PBPIP1	KM056639
468102	2	Passeriformes	Muscicapidae	*Cossypha*	*anomala*	*macclounii*	Olive-flanked Robin-Chat	*Leucocytozoon spp.*	Coinfection	*Leucocytozoon* spp.
468097	2	Passeriformes	Muscicapidae	*Cossypha*	*anomala*	*macclounii*	Olive-flanked Robin-Chat	*Parahaemoproteus* sp.	H_AFR121	KM056427
468098	2	Passeriformes	Muscicapidae	*Cossypha*	*anomala*	*macclounii*	Olive-flanked Robin-Chat	*Parahaemoproteus* sp.	H_AFR122	KM056428
468103	2	Passeriformes	Muscicapidae	*Cossypha*	*anomala*	*macclounii*	Olive-flanked Robin-Chat	*Leucocytozoon* sp.	L_AFR186	KM056504
468107	2	Passeriformes	Muscicapidae	*Cossypha*	*anomala*	*macclounii*	Olive-flanked Robin-Chat	*Leucocytozoon* sp.	L_AFR222	KM056536
468100	2	Passeriformes	Muscicapidae	*Cossypha*	*anomala*	*macclounii*	Olive-flanked Robin-Chat	*Leucocytozoon* sp.	L_AFR226	KM056540
468097	2	Passeriformes	Muscicapidae	*Cossypha*	*anomala*	*macclounii*	Olive-flanked Robin-Chat	*Leucocytozoon* sp.	L_AFR239	KM056553
468098	2	Passeriformes	Muscicapidae	*Cossypha*	*anomala*	*macclounii*	Olive-flanked Robin-Chat	*Leucocytozoon* sp.	L_AFR239	KM056553
468101	2	Passeriformes	Muscicapidae	*Cossypha*	*anomala*	*macclounii*	Olive-flanked Robin-Chat	*Leucocytozoon* sp.	L_WW6	KM056645
468106	2	Passeriformes	Muscicapidae	*Cossypha*	*anomala*	*macclounii*	Olive-flanked Robin-Chat	*Leucocytozoon* sp.	L_WW6	KM056645
468106	2	Passeriformes	Muscicapidae	*Cossypha*	*anomala*	*macclounii*	Olive-flanked Robin-Chat	*Leucocytozoon* sp.	L_ZOABY02	KM056650
468105	2	Passeriformes	Muscicapidae	*Cossypha*	*anomala*	*macclounii*	Olive-flanked Robin-Chat	0	NA	NA
468103	2	Passeriformes	Muscicapidae	*Cossypha*	*anomala*	*macclounii*	Olive-flanked Robin-Chat	*Plasmodium* sp.	P_BUL07	KM056642
468107	2	Passeriformes	Muscicapidae	*Cossypha*	*anomala*	*macclounii*	Olive-flanked Robin-Chat	*Plasmodium* sp.	P_MALNI02	KM056641
468091	2	Passeriformes	Muscicapidae	*Cossypha*	*caffra*	*iolaema*	Cape Robin-Chat	*Leucocytozoon* sp.	L_AFR215	KM056532
468094	2	Passeriformes	Muscicapidae	*Cossypha*	*caffra*	*iolaema*	Cape Robin-Chat	*Leucocytozoon* sp.	L_AFR222	KM056536
468086	2	Passeriformes	Muscicapidae	*Cossypha*	*caffra*	*iolaema*	Cape Robin-Chat	*Leucocytozoon* sp.	L_AFR225	KM056539
468096	2	Passeriformes	Muscicapidae	*Cossypha*	*caffra*	*iolaema*	Cape Robin-Chat	*Leucocytozoon* sp.	L_AFR229	KM056543
468086	2	Passeriformes	Muscicapidae	*Cossypha*	*caffra*	*iolaema*	Cape Robin-Chat	*Leucocytozoon* sp.	L_RECOB3	KM056648
468092	2	Passeriformes	Muscicapidae	*Cossypha*	*caffra*	*iolaema*	Cape Robin-Chat	*Leucocytozoon* sp.	L_RECOB3	KM056648
468089	2	Passeriformes	Muscicapidae	*Cossypha*	*caffra*	*iolaema*	Cape Robin-Chat	*Leucocytozoon* sp.	L_ZOABY02	KM056650
468093	2	Passeriformes	Muscicapidae	*Cossypha*	*caffra*	*iolaema*	Cape Robin-Chat	0	NA	NA
468095	2	Passeriformes	Muscicapidae	*Cossypha*	*caffra*	*iolaema*	Cape Robin-Chat	0	NA	NA
468091	2	Passeriformes	Muscicapidae	*Cossypha*	*caffra*	*iolaema*	Cape Robin-Chat	*Plasmodium* sp.	P_AFR108	KM056569
468085	2	Passeriformes	Muscicapidae	*Cossypha*	*caffra*	*iolaema*	Cape Robin-Chat	*Plasmodium* sp.	P_AFR93	KM056617
468085	2	Passeriformes	Muscicapidae	*Cossypha*	*caffra*	*iolaema*	Cape Robin-Chat	*Plasmodium* sp.	P_AFR94	KM056618
468090	2	Passeriformes	Muscicapidae	*Cossypha*	*caffra*	*iolaema*	Cape Robin-Chat	*Plasmodium* sp.	P_BT8	KM056624
468096	2	Passeriformes	Muscicapidae	*Cossypha*	*caffra*	*iolaema*	Cape Robin-Chat	*Plasmodium* sp.	P_BUL07	KM056642
468081	2	Passeriformes	Muscicapidae	*Cossypha*	*heuglini*	*heuglini*	White-browed Robin-Chat	*Parahaemoproteus* sp.	H_AFR144	KM056436
468083	2	Passeriformes	Muscicapidae	*Cossypha*	*heuglini*	*heuglini*	White-browed Robin-Chat	*Parahaemoproteus* sp.	H_AFR59	KM056456
468082	2	Passeriformes	Muscicapidae	*Cossypha*	*heuglini*	*heuglini*	White-browed Robin-Chat	*Parahaemoproteus* sp.	H_QUERY01	KM056416
468083	2	Passeriformes	Muscicapidae	*Cossypha*	*heuglini*	*heuglini*	White-browed Robin-Chat	*Leucocytozoon* sp.	L_AFR205	KM056523
468084	2	Passeriformes	Muscicapidae	*Cossypha*	*heuglini*	*heuglini*	White-browed Robin-Chat	*Leucocytozoon* sp.	L_AFR212	KM056530
468080	1	Passeriformes	Muscicapidae	*Cossypha*	*heuglini*	*heuglini*	White-browed Robin-Chat	*Leucocytozoon* sp.	L_AFR218	KM056533
468078	1	Passeriformes	Muscicapidae	*Cossypha*	*heuglini*	*heuglini*	White-browed Robin-Chat	0	NA	NA
468083	2	Passeriformes	Muscicapidae	*Cossypha*	*heuglini*	*heuglini*	White-browed Robin-Chat	*Plasmodium* sp.	P_AFR147	KM056588
468080	1	Passeriformes	Muscicapidae	*Cossypha*	*heuglini*	*heuglini*	White-browed Robin-Chat	*Plasmodium* sp.	P_COLL11	KM056636
468084	2	Passeriformes	Muscicapidae	*Cossypha*	*heuglini*	*heuglini*	White-browed Robin-Chat	*Plasmodium* sp.	P_GRW09	KM056631
468131	2	Passeriformes	Muscicapidae	*Erithacus*	*sharpei*	*sharpei*	Sharpe's Akalat	*Parahaemoproteus* sp.	H_AFR122	KM056428
468133	2	Passeriformes	Muscicapidae	*Erithacus*	*sharpei*	*sharpei*	Sharpe's Akalat	*Parahaemoproteus* sp.	H_AFR122	KM056428
468138	2	Passeriformes	Muscicapidae	*Erithacus*	*sharpei*	*sharpei*	Sharpe's Akalat	*Parahaemoproteus* sp.	H_AFR139	KM056434
468133	2	Passeriformes	Muscicapidae	*Erithacus*	*sharpei*	*sharpei*	Sharpe's Akalat	*Leucocytozoon* sp.	L_AFR211	KM056529
468135	2	Passeriformes	Muscicapidae	*Erithacus*	*sharpei*	*sharpei*	Sharpe's Akalat	*Leucocytozoon* sp.	L_AFR220	KM056535
468134	2	Passeriformes	Muscicapidae	*Erithacus*	*sharpei*	*sharpei*	Sharpe's Akalat	*Leucocytozoon* sp.	L_AFR229	KM056543
468132	2	Passeriformes	Muscicapidae	*Erithacus*	*sharpei*	*sharpei*	Sharpe's Akalat	*Plasmodium* sp.	P_BUL07	KM056642
468135	2	Passeriformes	Muscicapidae	*Erithacus*	*sharpei*	*sharpei*	Sharpe's Akalat	*Plasmodium* sp.	P_BUL07	KM056642
468044	1	Passeriformes	Muscicapidae	*Erythropygia*	*barbata*		Miombo Scrub-Robin	*Plasmodium* sp.	P_AFR65	KM056607
468043	1	Passeriformes	Muscicapidae	*Erythropygia*	*barbata*		Miombo Scrub-Robin	*Plasmodium* sp.	P_COLL11	KM056636
468045	1	Passeriformes	Muscicapidae	*Erythropygia*	*leucophrys*	*zambesiana*	Red-backed Scrub Robin	*Leucocytozoon* sp.	L_AFR154	KM056474
468045	1	Passeriformes	Muscicapidae	*Erythropygia*	*leucophrys*	*zambesiana*	Red-backed Scrub Robin	*Plasmodium* sp.	P_COLL11	KM056636
468046	1	Passeriformes	Muscicapidae	*Erythropygia*	*leucophrys*	*zambesiana*	Red-backed Scrub Robin	*Plasmodium* sp.	P_COLL11	KM056636

468047	1	Passeriformes	Muscicapidae	*Erythropygia*	*leucophrys*	*zambesiana*	Red-backed Scrub Robin	*Plasmodium* sp.	P_COLL11	KM056636
468048	1	Passeriformes	Muscicapidae	*Erythropygia*	*leucophrys*	*zambesiana*	Red-backed Scrub Robin	*Plasmodium* sp.	P_PSEGRI01	KM056637
468309	2	Passeriformes	Muscicapidae	*Ficedula*	*albicollis*	*albicollis*	Collared Flycatcher	*Haemoproteus pallidus*	H_COLL2	KM056413
468301	2	Passeriformes	Muscicapidae	*Muscicapa*	*adusta*	*subadusta*	African Dusky Flycatcher	Unknown	Coinfection	NA
468302	2	Passeriformes	Muscicapidae	*Muscicapa*	*adusta*	*subadusta*	African Dusky Flycatcher	*Parahaemoproteus* sp.	H_AFR150	KM056439
468307	2	Passeriformes	Muscicapidae	*Muscicapa*	*coerulescens*	*impavida*	Ashy Flycatcher	*Parahaemoproteus* sp.	H_AFR148	KM056437
468111	1	Passeriformes	Muscicapidae	*Myrmecocichla*	*arnotti*	*arnotti*	White-headed Black-Chat	*Plasmodium* sp.	P_AFR23	KM056591
468113	1	Passeriformes	Muscicapidae	*Myrmecocichla*	*arnotti*	*arnotti*	White-headed Black-Chat	*Plasmodium* sp.	P_AFR68	KM056608
468111	1	Passeriformes	Muscicapidae	*Myrmecocichla*	*arnotti*	*arnotti*	White-headed Black-Chat	*Plasmodium* sp.	P_BT8	KM056624
468113	1	Passeriformes	Muscicapidae	*Myrmecocichla*	*arnotti*	*arnotti*	White-headed Black-Chat	*Plasmodium* sp.	P_RFF1	KM056632
468066	2	Passeriformes	Muscicapidae	*Pogonocichla*	*stellata*	*orientalis*	White-starred Robin	*Parahaemoproteus* sp.	H_AFR126	KM056429
468059	2	Passeriformes	Muscicapidae	*Pogonocichla*	*stellata*	*orientalis*	White-starred Robin	*Parahaemoproteus* sp.	H_AFR153	KM056441
468060	2	Passeriformes	Muscicapidae	*Pogonocichla*	*stellata*	*orientalis*	White-starred Robin	*Parahaemoproteus* sp.	H_AFR25	KM056444
468068	2	Passeriformes	Muscicapidae	*Pogonocichla*	*stellata*	*orientalis*	White-starred Robin	*Leucocytozoon* sp.	L_AFR196	KM056514
468063	2	Passeriformes	Muscicapidae	*Pogonocichla*	*stellata*	*orientalis*	White-starred Robin	*Leucocytozoon* sp.	L_AFR211	KM056529
468070	2	Passeriformes	Muscicapidae	*Pogonocichla*	*stellata*	*orientalis*	White-starred Robin	*Leucocytozoon* sp.	L_AFR237	KM056551
468062	2	Passeriformes	Muscicapidae	*Pogonocichla*	*stellata*	*orientalis*	White-starred Robin	*Leucocytozoon* sp.	L_RECOB3	KM056648
468056	2	Passeriformes	Muscicapidae	*Pogonocichla*	*stellata*	*orientalis*	White-starred Robin	0	NA	NA
468057	2	Passeriformes	Muscicapidae	*Pogonocichla*	*stellata*	*orientalis*	White-starred Robin	0	NA	NA
468061	2	Passeriformes	Muscicapidae	*Pogonocichla*	*stellata*	*orientalis*	White-starred Robin	0	NA	NA
468067	2	Passeriformes	Muscicapidae	*Pogonocichla*	*stellata*	*orientalis*	White-starred Robin	0	NA	NA
468062	2	Passeriformes	Muscicapidae	*Pogonocichla*	*stellata*	*orientalis*	White-starred Robin	*Plasmodium* sp.	P_AFR124	KM056575
468064	2	Passeriformes	Muscicapidae	*Pogonocichla*	*stellata*	*orientalis*	White-starred Robin	*Plasmodium* sp.	P_AFR125	KM056576
468069	2	Passeriformes	Muscicapidae	*Pogonocichla*	*stellata*	*orientalis*	White-starred Robin	*Plasmodium* sp.	P_AFR134	KM056582
468062	2	Passeriformes	Muscicapidae	*Pogonocichla*	*stellata*	*orientalis*	White-starred Robin	*Plasmodium* sp.	P_BUL07	KM056642
468058	2	Passeriformes	Muscicapidae	*Pogonocichla*	*stellata*	*orientalis*	White-starred Robin	*Plasmodium* sp.	P_GRW09	KM056631
468065	2	Passeriformes	Muscicapidae	*Pogonocichla*	*stellata*	*orientalis*	White-starred Robin	*Plasmodium* sp.	P_GRW09	KM056631
468055	2	Passeriformes	Muscicapidae	*Pogonocichla*	*stellata*	*orientalis*	White-starred Robin	*Plasmodium* sp.	P_MALNI02	KM056641
468050	2	Passeriformes	Muscicapidae	*Pogonocichla*	*stellata*	*orientalis*	White-starred Robin	*Plasmodium* sp.	P_PSEGRI01	KM056637
468062	2	Passeriformes	Muscicapidae	*Pogonocichla*	*stellata*	*orientalis*	White-starred Robin	*Plasmodium* sp.	P_PSEGRI01	KM056637
468064	2	Passeriformes	Muscicapidae	*Pogonocichla*	*stellata*	*orientalis*	White-starred Robin	*Plasmodium* sp.	P_PSEGRI01	KM056637
468070	2	Passeriformes	Muscicapidae	*Pogonocichla*	*stellata*	*orientalis*	White-starred Robin	*Plasmodium* sp.	P_PSEGRI01	KM056637
468115	1	Passeriformes	Muscicapidae	*Saxicola*	*torquata*	*promiscua*	Common Stonechat	0	NA	NA
468116	2	Passeriformes	Muscicapidae	*Saxicola*	*torquata*	*promiscua*	Common Stonechat	0	NA	NA
468339	2	Passeriformes	Nectariniidae	*Nectarinia*	*afra*	*whytei*	Montane Double-collared Sunbird	*Parahaemoproteus* sp.	H_AFR111	KM056422
468344	2	Passeriformes	Nectariniidae	*Nectarinia*	*afra*	*whytei*	Montane Double-collared Sunbird	*Parahaemoproteus* sp.	H_AFR116	KM056424
468341	2	Passeriformes	Nectariniidae	*Nectarinia*	*afra*	*whytei*	Montane Double-collared Sunbird	*Parahaemoproteus* sp.	H_AFR8	KM056468
468342	2	Passeriformes	Nectariniidae	*Nectarinia*	*afra*	*whytei*	Montane Double-collared Sunbird	*Leucocytozoon* sp.	L_AFR214	KM056531
468343	2	Passeriformes	Nectariniidae	*Nectarinia*	*afra*	*whytei*	Montane Double-collared Sunbird	0	NA	NA
468345	2	Passeriformes	Nectariniidae	*Nectarinia*	*afra*	*whytei*	Montane Double-collared Sunbird	0	NA	NA
468351	1	Passeriformes	Nectariniidae	*Nectarinia*	*amethystina*	*kirkii*	Amethyst Sunbird	*Parahaemoproteus* sp.	H_AFR85	KM056471
468351	1	Passeriformes	Nectariniidae	*Nectarinia*	*amethystina*	*kirkii*	Amethyst Sunbird	*Leucocytozoon* sp.	L_AFR164	KM056483
468324	2	Passeriformes	Nectariniidae	*Nectarinia*	*famosa*	*aeneigularis*	Malachite Sunbird	0	NA	NA
468326	2	Passeriformes	Nectariniidae	*Nectarinia*	*famosa*	*aeneigularis*	Malachite Sunbird	0	NA	NA
468352	2	Passeriformes	Nectariniidae	*Nectarinia*	*kilimensis*	*arturi*	Bronze Sunbird	*Parahaemoproteus* sp.	H_AFR116	KM056424
468333	2	Passeriformes	Nectariniidae	*Nectarinia*	*mediocris*	*fuelleborni*	Eastern Double-collard Sunbird	*Parahaemoproteus* sp.	H_AFR116	KM056424
468334	2	Passeriformes	Nectariniidae	*Nectarinia*	*mediocris*	*fuelleborni*	Eastern Double-collard Sunbird	*Parahaemoproteus* sp.	H_ZOSMAD01	KM056404
468335	2	Passeriformes	Nectariniidae	*Nectarinia*	*mediocris*	*fuelleborni*	Eastern Double-collard Sunbird	*Leucocytozoon* sp.	L_AFR184	KM056502
468334	2	Passeriformes	Nectariniidae	*Nectarinia*	*mediocris*	*fuelleborni*	Eastern Double-collard Sunbird	*Leucocytozoon* sp.	L_AFR219	KM056534
468338	2	Passeriformes	Nectariniidae	*Nectarinia*	*mediocris*	*fuelleborni*	Eastern Double-collard Sunbird	*Leucocytozoon* sp.	L_AFR246	KM056559
468331	2	Passeriformes	Nectariniidae	*Nectarinia*	*mediocris*	*fuelleborni*	Eastern Double-collard Sunbird	0	NA	NA
468332	2	Passeriformes	Nectariniidae	*Nectarinia*	*mediocris*	*fuelleborni*	Eastern Double-collard Sunbird	0	NA	NA
468335	2	Passeriformes	Nectariniidae	*Nectarinia*	*mediocris*	*fuelleborni*	Eastern Double-collard Sunbird	*Plasmodium* sp.	P_AFR127	KM056577

468337	2	Passeriformes	Nectariniidae	*Nectarinia*	*mediocris*	*fuelleborni*	Eastern Double-collard Sunbird	*Plasmodium* sp.	P_AFR129	KM056578
468336	2	Passeriformes	Nectariniidae	*Nectarinia*	*mediocris*	*fuelleborni*	Eastern Double-collard Sunbird	*Plasmodium* sp.	P_AFR152	KM056589
468335	2	Passeriformes	Nectariniidae	*Nectarinia*	*mediocris*	*fuelleborni*	Eastern Double-collard Sunbird	*Plasmodium* sp.	P_AFR6	KM056605
468336	2	Passeriformes	Nectariniidae	*Nectarinia*	*mediocris*	*fuelleborni*	Eastern Double-collard Sunbird	*Plasmodium* sp.	P_PSEGRI01	KM056637
468330	2	Passeriformes	Nectariniidae	*Nectarinia*	*mediocris*	*fuelleborni*	Eastern Double-collard Sunbird	*Plasmodium* sp.	P_PSEGRI01	KM056637
468318	2	Passeriformes	Nectariniidae	*Nectarinia*	*olivacea*	*alfredi*	Olive Sunbird	Unknown	Coinfection	NA
468318	2	Passeriformes	Nectariniidae	*Nectarinia*	*olivacea*	*alfredi*	Olive Sunbird	*Haemoproteus cyanomitrae*	H_CYAOLI05	KM056417
468319	2	Passeriformes	Nectariniidae	*Nectarinia*	*olivacea*	*alfredi*	Olive Sunbird	*Haemoproteus cyanomitrae*	H_CYAOLI05	KM056417
468320	2	Passeriformes	Nectariniidae	*Nectarinia*	*olivacea*	*alfredi*	Olive Sunbird	*Haemoproteus cyanomitrae*	H_CYAOLI05	KM056417
468321	2	Passeriformes	Nectariniidae	*Nectarinia*	*olivacea*	*alfredi*	Olive Sunbird	*Haemoproteus cyanomitrae*	H_CYAOLI05	KM056417
468323	2	Passeriformes	Nectariniidae	*Nectarinia*	*olivacea*	*alfredi*	Olive Sunbird	*Haemoproteus cyanomitrae*	H_CYAOLI05	KM056417
468323	2	Passeriformes	Nectariniidae	*Nectarinia*	*olivacea*	*alfredi*	Olive Sunbird	*Leucocytozoon* sp.	L_AFR204	KM056522
468323	2	Passeriformes	Nectariniidae	*Nectarinia*	*olivacea*	*alfredi*	Olive Sunbird	*Leucocytozoon* sp.	L_ANLAT16	KM056643
468350	1	Passeriformes	Nectariniidae	*Nectarinia*	*senegalensis*	*gutturalis*	Scarlet-chested Sunbird	*Parahaemoproteus* sp.	H_AFR78	KM056467
468348	1	Passeriformes	Nectariniidae	*Nectarinia*	*senegalensis*	*gutturalis*	Scarlet-chested Sunbird	*Plasmodium* sp.	P_CYAOLI04	KM056640
468346	1	Passeriformes	Nectariniidae	*Nectarinia*	*venusta*	*falkensteini*	Variable Sunbird	*Parahaemoproteus* sp.	H_AFR62	KM056458
468347	1	Passeriformes	Nectariniidae	*Nectarinia*	*venusta*	*falkensteini*	Variable Sunbird	*Plasmodium* sp.	P_AFR10	KM056563
468347	1	Passeriformes	Nectariniidae	*Nectarinia*	*venusta*	*falkensteini*	Variable Sunbird	*Plasmodium* sp.	P_COLL7	KM056625
468613	1	Passeriformes	Oriolidae	*Oriolus*	*auratus*	*notatus*	African Golden-Oriole	Unknown	Coinfection	NA
468609	1	Passeriformes	Oriolidae	*Oriolus*	*auratus*	*notatus*	African Golden-Oriole	*Parahaemoproteus* sp.	H_AFR53	KM056454
468315	2	Passeriformes	Paridae	*Parus*	*griseiventris*		Miombo Tit	*Leucocytozoon spp.*	Coinfection	*Leucocytozoon* spp.
468315	2	Passeriformes	Paridae	*Parus*	*griseiventris*		Miombo Tit	*Plasmodium* sp.	P_AFR110	KM056570
468312	1	Passeriformes	Paridae	*Parus*	*niger*	*niger*	Southern Black Tit	*Leucocytozoon* sp.	L_AFR159	KM056479
468312	1	Passeriformes	Paridae	*Parus*	*niger*	*niger*	Southern Black Tit	*Plasmodium* sp.	P_AFR60	KM056606
468314	1	Passeriformes	Passeridae	*Parus*	*rufiventris*		Rufous-bellied Tit	*Leucocytozoon* sp.	L_AFR163	KM056482
468486	1	Passeriformes	Passeridae	*Petronia*	*superciliaris*		Yellow-throated Petronia	*Parahaemoproteus* sp.	H_AFR29	KM056445
468488	1	Passeriformes	Passeridae	*Petronia*	*superciliaris*		Yellow-throated Petronia	*Parahaemoproteus* sp.	H_AFR42	KM056449
468493	1	Passeriformes	Passeridae	*Petronia*	*superciliaris*		Yellow-throated Petronia	*Plasmodium* sp.	P_AFR10	KM056563
468493	1	Passeriformes	Passeridae	*Petronia*	*superciliaris*		Yellow-throated Petronia	*Plasmodium* sp.	P_BUL07	KM056642
468491	1	Passeriformes	Passeridae	*Petronia*	*superciliaris*		Yellow-throated Petronia	*Plasmodium* sp.	P_GRW09	KM056631
468495	1	Passeriformes	Passeridae	*Plocepasser*	*rufoscapulatus*		Chestnut-backed Sparrow-Weaver	0	NA	NA
468496	1	Passeriformes	Passeridae	*Plocepasser*	*rufoscapulatus*		Chestnut-backed Sparrow-Weaver	*Plasmodium* sp.	P_PSEGRI01	KM056637
468273	2	Passeriformes	Platysteiridae	*Batis*	*dimorpha*	*sola*	Malawi Batis	*Leucocytozoon spp.*	Coinfection	*Leucocytozoon* spp.
468274	2	Passeriformes	Platysteiridae	*Batis*	*dimorpha*	*sola*	Malawi Batis	*Leucocytozoon spp.*	Coinfection	*Leucocytozoon* spp.
468276	2	Passeriformes	Platysteiridae	*Batis*	*dimorpha*	*sola*	Malawi Batis	*Leucocytozoon spp.*	Coinfection	*Leucocytozoon* spp.
468288	2	Passeriformes	Platysteiridae	*Batis*	*dimorpha*	*sola*	Malawi Batis	*Leucocytozoon spp.*	Coinfection	*Leucocytozoon* spp.
468278	2	Passeriformes	Platysteiridae	*Batis*	*dimorpha*	*sola*	Malawi Batis	Unknown	Coinfection	NA
468287	2	Passeriformes	Platysteiridae	*Batis*	*dimorpha*	*sola*	Malawi Batis	*Parahaemoproteus* sp.	H_AFR149	KM056438
468286	2	Passeriformes	Platysteiridae	*Batis*	*dimorpha*	*sola*	Malawi Batis	*Parahaemoproteus* sp.	H_AFR59	KM056456
468278	2	Passeriformes	Platysteiridae	*Batis*	*dimorpha*	*sola*	Malawi Batis	*Parahaemoproteus* sp.	H_AFR8	KM056468
468279	2	Passeriformes	Platysteiridae	*Batis*	*dimorpha*	*sola*	Malawi Batis	*Leucocytozoon* sp.	L_AFR180	KM056498
468282	2	Passeriformes	Platysteiridae	*Batis*	*dimorpha*	*sola*	Malawi Batis	*Leucocytozoon* sp.	L_AFR190	KM056508
468283	2	Passeriformes	Platysteiridae	*Batis*	*dimorpha*	*sola*	Malawi Batis	*Leucocytozoon* sp.	L_AFR193	KM056511
468287	2	Passeriformes	Platysteiridae	*Batis*	*dimorpha*	*sola*	Malawi Batis	*Leucocytozoon* sp.	L_AFR206	KM056524
468266	2	Passeriformes	Platysteiridae	*Batis*	*dimorpha*	*sola*	Malawi Batis	*Leucocytozoon* sp.	L_AFR211	KM056529
468279	2	Passeriformes	Platysteiridae	*Batis*	*dimorpha*	*sola*	Malawi Batis	*Leucocytozoon* sp.	L_AFR224	KM056538
468280	2	Passeriformes	Platysteiridae	*Batis*	*dimorpha*	*sola*	Malawi Batis	*Leucocytozoon* sp.	L_AFR224	KM056538
468281	2	Passeriformes	Platysteiridae	*Batis*	*dimorpha*	*sola*	Malawi Batis	*Leucocytozoon* sp.	L_AFR224	KM056538
468282	2	Passeriformes	Platysteiridae	*Batis*	*dimorpha*	*sola*	Malawi Batis	*Leucocytozoon* sp.	L_AFR229	KM056543
468286	2	Passeriformes	Platysteiridae	*Batis*	*dimorpha*	*sola*	Malawi Batis	*Leucocytozoon* sp.	L_AFR247	KM056560
468267	2	Passeriformes	Platysteiridae	*Batis*	*dimorpha*	*sola*	Malawi Batis	0	NA	NA
468272	2	Passeriformes	Platysteiridae	*Batis*	*dimorpha*	*sola*	Malawi Batis	0	NA	NA
468275	2	Passeriformes	Platysteiridae	*Batis*	*dimorpha*	*sola*	Malawi Batis	0	NA	NA

468277	2	Passeriformes	Platysteiridae	*Batis*	*dimorpha*	*sola*	Malawi Batis	*Plasmodium* sp.	P_AFR117	KM056572
468281	2	Passeriformes	Platysteiridae	*Batis*	*dimorpha*	*sola*	Malawi Batis	*Plasmodium* sp.	P_AFR131	KM056580
468286	2	Passeriformes	Platysteiridae	*Batis*	*dimorpha*	*sola*	Malawi Batis	*Plasmodium* sp.	P_AFR135	KM056583
468283	2	Passeriformes	Platysteiridae	*Batis*	*dimorpha*	*sola*	Malawi Batis	*Plasmodium* sp.	P_BUL07	KM056642
468268	2	Passeriformes	Platysteiridae	*Batis*	*dimorpha*	*sola*	Malawi Batis	*Plasmodium* sp.	P_COLL11	KM056636
468274	2	Passeriformes	Platysteiridae	*Batis*	*dimorpha*	*sola*	Malawi Batis	*Plasmodium* sp.	P_MALNI02	KM056641
468276	2	Passeriformes	Platysteiridae	*Batis*	*dimorpha*	*sola*	Malawi Batis	*Plasmodium* sp.	P_PSEGRI01	KM056637
468265	1	Passeriformes	Platysteiridae	*Batis*	*molitor*	*palliditergum*	Chinspot Batis	0	NA	NA
468264	1	Passeriformes	Platysteiridae	*Batis*	*molitor*	*palliditergum*	Chinspot Batis	*Plasmodium* sp.	P_AFR28	KM056593
468558	1	Passeriformes	Ploceidae	*Euplectes*	*albonotatus*	*albonotatus*	White-winged Widowbird	*Leucocytozoon* sp.	L_AFR225	KM056539
468557	1	Passeriformes	Ploceidae	*Euplectes*	*albonotatus*	*albonotatus*	White-winged Widowbird	0	NA	NA
468563	1	Passeriformes	Ploceidae	*Euplectes*	*albonotatus*	*albonotatus*	White-winged Widowbird	*Plasmodium* sp.	P_AFR86	KM056613
468559	1	Passeriformes	Ploceidae	*Euplectes*	*albonotatus*	*albonotatus*	White-winged Widowbird	*Plasmodium* sp.	P_COLL7	KM056625
468560	1	Passeriformes	Ploceidae	*Euplectes*	*albonotatus*	*albonotatus*	White-winged Widowbird	*Plasmodium* sp.	P_COLL7	KM056625
468558	1	Passeriformes	Ploceidae	*Euplectes*	*albonotatus*	*albonotatus*	White-winged Widowbird	*Plasmodium* sp.	P_GRW10	KM056622
468559	1	Passeriformes	Ploceidae	*Euplectes*	*albonotatus*	*albonotatus*	White-winged Widowbird	*Plasmodium* sp.	P_RFF1	KM056632
468554	1	Passeriformes	Ploceidae	*Euplectes*	*ardens*	*ardens*	Red-collared Widowbird	*Parahaemoproteus* sp.	H_AFR19	KM056442
468554	1	Passeriformes	Ploceidae	*Euplectes*	*ardens*	*ardens*	Red-collared Widowbird	*Leucocytozoon* sp.	L_AFR156	KM056476
468554	1	Passeriformes	Ploceidae	*Euplectes*	*ardens*	*ardens*	Red-collared Widowbird	*Leucocytozoon* sp.	L_REB6	KM056644
468548	1	Passeriformes	Ploceidae	*Euplectes*	*ardens*	*ardens*	Red-collared Widowbird	*Leucocytozoon* sp.	L_SATEC01	NA
468547	1	Passeriformes	Ploceidae	*Euplectes*	*ardens*	*ardens*	Red-collared Widowbird	0	NA	NA
468553	1	Passeriformes	Ploceidae	*Euplectes*	*ardens*	*ardens*	Red-collared Widowbird	*Plasmodium* sp.	P_MALNI02	KM056641
468548	1	Passeriformes	Ploceidae	*Euplectes*	*ardens*	*ardens*	Red-collared Widowbird	*Plasmodium* sp.	P_RFF1	KM056632
468552	1	Passeriformes	Ploceidae	*Euplectes*	*ardens*	*ardens*	Red-collared Widowbird	*Plasmodium* sp.	P_WW3	NA
468552	1	Passeriformes	Ploceidae	*Euplectes*	*ardens*	*ardens*	Red-collared Widowbird	*Plasmodium* sp.	P_WW4	KM056626
468546	1	Passeriformes	Ploceidae	*Euplectes*	*capensis*	*crassirostris*	Yellow Bishop	Unknown	Coinfection	NA
468542	1	Passeriformes	Ploceidae	*Euplectes*	*capensis*	*crassirostris*	Yellow Bishop	*Leucocytozoon* sp.	L_AFR218	KM056533
468542	1	Passeriformes	Ploceidae	*Euplectes*	*capensis*	*crassirostris*	Yellow Bishop	*Plasmodium* sp.	P_AFR10	KM056563
468545	1	Passeriformes	Ploceidae	*Euplectes*	*capensis*	*crassirostris*	Yellow Bishop	*Plasmodium* sp.	P_GRW10	KM056622
468538	1	Passeriformes	Ploceidae	*Euplectes*	*capensis*	*crassirostris*	Yellow Bishop	*Plasmodium* sp.	P_RFF1	KM056632
468533	2	Passeriformes	Ploceidae	*Euplectes*	*hartlaubi*	*psammocromius*	Marsh Widowbird	*Leucocytozoon spp.*	Coinfection	*Leucocytozoon* spp.
468535	2	Passeriformes	Ploceidae	*Euplectes*	*hartlaubi*	*psammocromius*	Marsh Widowbird	*Leucocytozoon* sp.	L_AFR169	KM056488
468535	2	Passeriformes	Ploceidae	*Euplectes*	*hartlaubi*	*psammocromius*	Marsh Widowbird	*Leucocytozoon* sp.	L_AFR214	KM056531
468536	2	Passeriformes	Ploceidae	*Euplectes*	*hartlaubi*	*psammocromius*	Marsh Widowbird	0	NA	NA
468533	2	Passeriformes	Ploceidae	*Euplectes*	*hartlaubi*	*psammocromius*	Marsh Widowbird	*Plasmodium* sp.	P_BUL07	KM056642
468505	2	Passeriformes	Ploceidae	*Ploceus*	*baglafecht*	*nyikae*	Baglafecht Weaver	*Leucocytozoon spp.*	Coinfection	*Leucocytozoon* spp.
468497	2	Passeriformes	Ploceidae	*Ploceus*	*baglafecht*	*nyikae*	Baglafecht Weaver	*Leucocytozoon* sp.	L_AFR168	KM056487
468498	2	Passeriformes	Ploceidae	*Ploceus*	*baglafecht*	*nyikae*	Baglafecht Weaver	*Leucocytozoon* sp.	L_AFR170	KM056489
468502	2	Passeriformes	Ploceidae	*Ploceus*	*baglafecht*	*nyikae*	Baglafecht Weaver	*Leucocytozoon* sp.	L_AFR172	KM056490
468498	2	Passeriformes	Ploceidae	*Ploceus*	*baglafecht*	*nyikae*	Baglafecht Weaver	*Leucocytozoon* sp.	L_AFR228	KM056542
468500	2	Passeriformes	Ploceidae	*Ploceus*	*baglafecht*	*nyikae*	Baglafecht Weaver	*Leucocytozoon* sp.	L_RECOB3	KM056648
468499	2	Passeriformes	Ploceidae	*Ploceus*	*baglafecht*	*nyikae*	Baglafecht Weaver	*Leucocytozoon* sp.	L_SATEC01	NA
468502	2	Passeriformes	Ploceidae	*Ploceus*	*baglafecht*	*nyikae*	Baglafecht Weaver	*Leucocytozoon* sp.	L_SATEC01	NA
468503	2	Passeriformes	Ploceidae	*Ploceus*	*baglafecht*	*nyikae*	Baglafecht Weaver	*Leucocytozoon* sp.	L_YMWD2	KM056649
468506	2	Passeriformes	Ploceidae	*Ploceus*	*baglafecht*	*nyikae*	Baglafecht Weaver	0	NA	NA
468501	2	Passeriformes	Ploceidae	*Ploceus*	*baglafecht*	*nyikae*	Baglafecht Weaver	*Plasmodium* sp.	P_AFR28	KM056593
468504	2	Passeriformes	Ploceidae	*Ploceus*	*baglafecht*	*nyikae*	Baglafecht Weaver	*Plasmodium* sp.	P_AFR6	KM056605
468505	2	Passeriformes	Ploceidae	*Ploceus*	*baglafecht*	*nyikae*	Baglafecht Weaver	*Plasmodium* sp.	P_LINOLI01	KM056629
468522	1	Passeriformes	Ploceidae	*Ploceus*	*ocularis*	*suahelicus*	Spectacled Weaver	*Leucocytozoon* sp.	L_AFR218	KM056533
468523	1	Passeriformes	Ploceidae	*Ploceus*	*ocularis*	*suahelicus*	Spectacled Weaver	*Plasmodium* sp.	P_AFR10	KM056563
468521	1	Passeriformes	Ploceidae	*Ploceus*	*ocularis*	*suahelicus*	Spectacled Weaver	*Plasmodium* sp.	P_AFR49	KM056600
468520	1	Passeriformes	Ploceidae	*Ploceus*	*ocularis*	*suahelicus*	Spectacled Weaver	*Plasmodium* sp.	P_MALNI02	KM056641
468521	1	Passeriformes	Ploceidae	*Ploceus*	*ocularis*	*suahelicus*	Spectacled Weaver	*Plasmodium* sp.	P_MALNI02	KM056641

468522	1	Passeriformes	Ploceidae	*Ploceus*	*ocularis*	*suahelicus*	Spectacled Weaver	*Plasmodium* sp.	P_MALNI02	KM056641
468576	1	Passeriformes	Ploceidae	*Ploceus*	*ocularis*	*suahelicus*	Spectacled Weaver	*Plasmodium* sp.	P_WW3	NA
468529	1	Passeriformes	Ploceidae	*Ploceus*	*velatus*	*shelleyi*	Southern Masked Weaver	*Leucocytozoon spp.*	Coinfection	*Leucocytozoon* spp.
468526	1	Passeriformes	Ploceidae	*Ploceus*	*velatus*	*shelleyi*	Southern Masked Weaver	*Leucocytozoon* sp.	L_AFR211	KM056529
468526	1	Passeriformes	Ploceidae	*Ploceus*	*velatus*	*shelleyi*	Southern Masked Weaver	*Plasmodium* sp.	P_AFR10	KM056563
468525	1	Passeriformes	Ploceidae	*Ploceus*	*velatus*	*shelleyi*	Southern Masked Weaver	*Plasmodium* sp.	P_BT8	KM056624
468527	1	Passeriformes	Ploceidae	*Ploceus*	*velatus*	*shelleyi*	Southern Masked Weaver	*Plasmodium* sp.	P_BT8	KM056624
468526	1	Passeriformes	Ploceidae	*Ploceus*	*velatus*	*shelleyi*	Southern Masked Weaver	*Plasmodium relictum*	P_LZFUS01	KM056627
468528	1	Passeriformes	Ploceidae	*Ploceus*	*velatus*	*shelleyi*	Southern Masked Weaver	*Plasmodium* sp.	P_MALNI02	KM056641
468529	1	Passeriformes	Ploceidae	*Ploceus*	*velatus*	*shelleyi*	Southern Masked Weaver	*Plasmodium* sp.	P_MALNI02	KM056641
468507	1	Passeriformes	Ploceidae	*Ploceus*	*xanthops*		Holub's Golden-Weaver	*Leucocytozoon* sp.	L_AFR155	KM056475
468509	1	Passeriformes	Ploceidae	*Ploceus*	*xanthops*		Holub's Golden-Weaver	*Leucocytozoon* sp.	L_AFR219	KM056534
468517	1	Passeriformes	Ploceidae	*Ploceus*	*xanthops*		Holub's Golden-Weaver	0	NA	NA
468514	1	Passeriformes	Ploceidae	*Ploceus*	*xanthops*		Holub's Golden-Weaver	*Plasmodium* sp.	P_BT8	KM056624
468507	1	Passeriformes	Ploceidae	*Ploceus*	*xanthops*		Holub's Golden-Weaver	*Plasmodium* sp.	P_MALNI02	KM056641
468508	1	Passeriformes	Ploceidae	*Ploceus*	*xanthops*		Holub's Golden-Weaver	*Plasmodium* sp.	P_MALNI02	KM056641
468509	1	Passeriformes	Ploceidae	*Ploceus*	*xanthops*		Holub's Golden-Weaver	*Plasmodium* sp.	P_MALNI02	KM056641
468510	1	Passeriformes	Ploceidae	*Ploceus*	*xanthops*		Holub's Golden-Weaver	*Plasmodium* sp.	P_MALNI02	KM056641
468511	1	Passeriformes	Ploceidae	*Ploceus*	*xanthops*		Holub's Golden-Weaver	*Plasmodium* sp.	P_MALNI02	KM056641
468513	1	Passeriformes	Ploceidae	*Ploceus*	*xanthops*		Holub's Golden-Weaver	*Plasmodium* sp.	P_MALNI02	KM056641
468514	1	Passeriformes	Ploceidae	*Ploceus*	*xanthops*		Holub's Golden-Weaver	*Plasmodium* sp.	P_MALNI02	KM056641
468515	1	Passeriformes	Ploceidae	*Ploceus*	*xanthops*		Holub's Golden-Weaver	*Plasmodium* sp.	P_MALNI02	KM056641
468516	1	Passeriformes	Ploceidae	*Ploceus*	*xanthops*		Holub's Golden-Weaver	*Plasmodium* sp.	P_MALNI02	KM056641
468518	1	Passeriformes	Ploceidae	*Ploceus*	*xanthops*		Holub's Golden-Weaver	*Plasmodium* sp.	P_MALNI02	KM056641
468512	1	Passeriformes	Ploceidae	*Ploceus*	*xanthops*		Holub's Golden-Weaver	*Plasmodium megalogluobularis*	P_PYSUN1	KM056628
468582	1	Passeriformes	Ploceidae	*Quelea*	*quelea*	*lathami*	Red-billed Quelea	Unknown	Coinfection	NA
468583	1	Passeriformes	Ploceidae	*Quelea*	*quelea*	*lathami*	Red-billed Quelea	Unknown	Coinfection	NA
468580	1	Passeriformes	Ploceidae	*Quelea*	*quelea*	*lathami*	Red-billed Quelea	*Parahaemoproteus* sp.	H_AFR29	KM056445
468581	1	Passeriformes	Ploceidae	*Quelea*	*quelea*	*lathami*	Red-billed Quelea	*Parahaemoproteus* sp.	H_QUERY01	KM056416
468585	1	Passeriformes	Ploceidae	*Quelea*	*quelea*	*lathami*	Red-billed Quelea	*Parahaemoproteus* sp.	H_RBQ11	KM056419
468585	1	Passeriformes	Ploceidae	*Quelea*	*quelea*	*lathami*	Red-billed Quelea	*Leucocytozoon* sp.	L_AFR166	KM056485
468577	1	Passeriformes	Ploceidae	*Quelea*	*quelea*	*lathami*	Red-billed Quelea	*Plasmodium* sp.	P_AFR10	KM056563
468579	1	Passeriformes	Ploceidae	*Quelea*	*quelea*	*lathami*	Red-billed Quelea	*Plasmodium* sp.	P_AFR10	KM056563
468584	1	Passeriformes	Ploceidae	*Quelea*	*quelea*	*lathami*	Red-billed Quelea	*Plasmodium* sp.	P_GRW09	KM056631
468577	1	Passeriformes	Ploceidae	*Quelea*	*quelea*	*lathami*	Red-billed Quelea	*Plasmodium* sp.	P_PLOVEL01	KM056630
468004	2	Passeriformes	Pycnonotidae	*Phyllastrephus*	*cerviniventris*		Grey-olive Greenbul	*Parahaemoproteus* sp.	H_AFR141	KM056435
468005	2	Passeriformes	Pycnonotidae	*Phyllastrephus*	*cerviniventris*		Grey-olive Greenbul	*Parahaemoproteus* sp.	H_AFR141	KM056435
468006	2	Passeriformes	Pycnonotidae	*Phyllastrephus*	*cerviniventris*		Grey-olive Greenbul	*Parahaemoproteus* sp.	H_AFR141	KM056435
468008	2	Passeriformes	Pycnonotidae	*Phyllastrephus*	*cerviniventris*		Grey-olive Greenbul	*Parahaemoproteus* sp.	H_QUERY01	KM056416
468008	2	Passeriformes	Pycnonotidae	*Phyllastrephus*	*cerviniventris*		Grey-olive Greenbul	*Leucocytozoon* sp.	L_AFR237	KM056551
468007	2	Passeriformes	Pycnonotidae	*Phyllastrephus*	*cerviniventris*		Grey-olive Greenbul	0	NA	NA
468012	2	Passeriformes	Pycnonotidae	*Phyllastrephus*	*flavostriatus*	*alfredi*	Sharpe's Yellow-streaked Greenbul	*Leucocytozoon spp.*	Coinfection	*Leucocytozoon* spp.
468013	2	Passeriformes	Pycnonotidae	*Phyllastrephus*	*flavostriatus*	*alfredi*	Sharpe's Yellow-streaked Greenbul	*Leucocytozoon spp.*	Coinfection	*Leucocytozoon* spp.
468011	2	Passeriformes	Pycnonotidae	*Phyllastrephus*	*flavostriatus*	*alfredi*	Sharpe's Yellow-streaked Greenbul	*Leucocytozoon* sp.	L_AFR226	KM056540
468015	2	Passeriformes	Pycnonotidae	*Phyllastrephus*	*flavostriatus*	*alfredi*	Sharpe's Yellow-streaked Greenbul	*Leucocytozoon* sp.	L_AFR226	KM056540
468010	2	Passeriformes	Pycnonotidae	*Phyllastrephus*	*flavostriatus*	*alfredi*	Sharpe's Yellow-streaked Greenbul	*Leucocytozoon* sp.	L_AFR227	KM056541
468009	2	Passeriformes	Pycnonotidae	*Phyllastrephus*	*flavostriatus*	*alfredi*	Sharpe's Yellow-streaked Greenbul	*Leucocytozoon* sp.	L_AFR234	KM056548
468010	2	Passeriformes	Pycnonotidae	*Phyllastrephus*	*flavostriatus*	*alfredi*	Sharpe's Yellow-streaked Greenbul	*Plasmodium* sp.	P_AFR123	KM056574
468009	2	Passeriformes	Pycnonotidae	*Phyllastrephus*	*flavostriatus*	*alfredi*	Sharpe's Yellow-streaked Greenbul	*Plasmodium* sp.	P_BUL07	KM056642
468010	2	Passeriformes	Pycnonotidae	*Phyllastrephus*	*flavostriatus*	*alfredi*	Sharpe's Yellow-streaked Greenbul	*Plasmodium* sp.	P_BUL07	KM056642
467999	1	Passeriformes	Pycnonotidae	*Pycnonotus*	*barbatus*	*layardi*	Dark-capped Bulbul	*Leucocytozoon spp.*	Coinfection	*Leucocytozoon* spp.
468001	1	Passeriformes	Pycnonotidae	*Pycnonotus*	*barbatus*	*layardi*	Dark-capped Bulbul	*Haemoproteus sanguinus*	H_BUL2	KM056412
467995	1	Passeriformes	Pycnonotidae	*Pycnonotus*	*barbatus*	*layardi*	Dark-capped Bulbul	*Haemoproteus belopolskyi*	H_MW1	KM056408

467994	1	Passeriformes	Pycnonotidae	*Pycnonotus*	*barbatus*	*layardi*	Dark-capped Bulbul	*Haemoproteus belopolskyi*	H_SW1	KM056409
467993	1	Passeriformes	Pycnonotidae	*Pycnonotus*	*barbatus*	*layardi*	Dark-capped Bulbul	*Leucocytozoon* sp.	L_AFR158	KM056478
468001	1	Passeriformes	Pycnonotidae	*Pycnonotus*	*barbatus*	*layardi*	Dark-capped Bulbul	*Leucocytozoon* sp.	L_AFR165	KM056484
468002	2	Passeriformes	Pycnonotidae	*Pycnonotus*	*barbatus*	*layardi*	Dark-capped Bulbul	*Leucocytozoon* sp.	L_AFR174	KM056492
468003	2	Passeriformes	Pycnonotidae	*Pycnonotus*	*barbatus*	*layardi*	Dark-capped Bulbul	*Leucocytozoon* sp.	L_AFR225	KM056539
467995	1	Passeriformes	Pycnonotidae	*Pycnonotus*	*barbatus*	*layardi*	Dark-capped Bulbul	*Leucocytozoon* sp.	L_AFR238	KM056552
467991	1	Passeriformes	Pycnonotidae	*Pycnonotus*	*barbatus*	*layardi*	Dark-capped Bulbul	0	NA	NA
468000	1	Passeriformes	Pycnonotidae	*Pycnonotus*	*barbatus*	*layardi*	Dark-capped Bulbul	*Plasmodium* sp.	P_BUL07	KM056642
468003	2	Passeriformes	Pycnonotidae	*Pycnonotus*	*barbatus*	*layardi*	Dark-capped Bulbul	*Plasmodium* sp.	P_BUL07	KM056642
467999	1	Passeriformes	Pycnonotidae	*Pycnonotus*	*barbatus*	*layardi*	Dark-capped Bulbul	*Plasmodium* sp.	P_GRW10	KM056622
467996	1	Passeriformes	Pycnonotidae	*Pycnonotus*	*barbatus*	*layardi*	Dark-capped Bulbul	*Plasmodium relictum*	P_LZFUS01	KM056627
467994	1	Passeriformes	Pycnonotidae	*Pycnonotus*	*barbatus*	*layardi*	Dark-capped Bulbul	*Plasmodium* sp.	P_PSEGRI01	KM056637
467966	2	Passeriformes	Pycnonotidae	*Pycnonotus*	*milanjensis*	*olivaceiceps*	Stripe-cheeked Greenbul	*Leucocytozoon spp.*	Coinfection	*Leucocytozoon* spp.
467964	2	Passeriformes	Pycnonotidae	*Pycnonotus*	*milanjensis*	*olivaceiceps*	Stripe-cheeked Greenbul	*Leucocytozoon* sp.	L_AFR194	KM056512
467965	2	Passeriformes	Pycnonotidae	*Pycnonotus*	*milanjensis*	*olivaceiceps*	Stripe-cheeked Greenbul	*Leucocytozoon* sp.	L_AFR220	KM056535
467965	2	Passeriformes	Pycnonotidae	*Pycnonotus*	*milanjensis*	*olivaceiceps*	Stripe-cheeked Greenbul	*Leucocytozoon* sp.	L_AFR230	KM056544
467964	2	Passeriformes	Pycnonotidae	*Pycnonotus*	*milanjensis*	*olivaceiceps*	Stripe-cheeked Greenbul	*Plasmodium* sp.	P_GRW09	KM056631
467966	2	Passeriformes	Pycnonotidae	*Pycnonotus*	*milanjensis*	*olivaceiceps*	Stripe-cheeked Greenbul	*Plasmodium* sp.	P_PSEGRI01	KM056637
467973	2	Passeriformes	Pycnonotidae	*Pycnonotus*	*tephrolaemus*	*fusciceps*	Southern Mountain Greenbul	*Leucocytozoon spp.*	Coinfection	*Leucocytozoon* spp.
467977	2	Passeriformes	Pycnonotidae	*Pycnonotus*	*tephrolaemus*	*fusciceps*	Southern Mountain Greenbul	*Leucocytozoon spp.*	Coinfection	*Leucocytozoon* spp.
467969	2	Passeriformes	Pycnonotidae	*Pycnonotus*	*tephrolaemus*	*fusciceps*	Southern Mountain Greenbul	Unknown	Coinfection	NA
467969	2	Passeriformes	Pycnonotidae	*Pycnonotus*	*tephrolaemus*	*fusciceps*	Southern Mountain Greenbul	*Parahaemoproteus* sp.	H_AFR103	KM056420
467971	2	Passeriformes	Pycnonotidae	*Pycnonotus*	*tephrolaemus*	*fusciceps*	Southern Mountain Greenbul	*Parahaemoproteus* sp.	H_AFR103	KM056420
467974	2	Passeriformes	Pycnonotidae	*Pycnonotus*	*tephrolaemus*	*fusciceps*	Southern Mountain Greenbul	*Parahaemoproteus* sp.	H_AFR103	KM056420
467978	2	Passeriformes	Pycnonotidae	*Pycnonotus*	*tephrolaemus*	*fusciceps*	Southern Mountain Greenbul	*Parahaemoproteus* sp.	H_AFR103	KM056420
467982	2	Passeriformes	Pycnonotidae	*Pycnonotus*	*tephrolaemus*	*fusciceps*	Southern Mountain Greenbul	*Parahaemoproteus* sp.	H_AFR103	KM056420
467980	2	Passeriformes	Pycnonotidae	*Pycnonotus*	*tephrolaemus*	*fusciceps*	Southern Mountain Greenbul	*Parahaemoproteus* sp.	H_AFR130	KM056430
467983	2	Passeriformes	Pycnonotidae	*Pycnonotus*	*tephrolaemus*	*fusciceps*	Southern Mountain Greenbul	*Parahaemoproteus* sp.	H_AFR130	KM056430
467981	2	Passeriformes	Pycnonotidae	*Pycnonotus*	*tephrolaemus*	*fusciceps*	Southern Mountain Greenbul	*Parahaemoproteus* sp.	H_AFR8	KM056468
467980	2	Passeriformes	Pycnonotidae	*Pycnonotus*	*tephrolaemus*	*fusciceps*	Southern Mountain Greenbul	*Leucocytozoon* sp.	L_AFR188	KM056506
467982	2	Passeriformes	Pycnonotidae	*Pycnonotus*	*tephrolaemus*	*fusciceps*	Southern Mountain Greenbul	*Leucocytozoon* sp.	L_AFR189	KM056507
467983	2	Passeriformes	Pycnonotidae	*Pycnonotus*	*tephrolaemus*	*fusciceps*	Southern Mountain Greenbul	*Leucocytozoon* sp.	L_AFR191	KM056509
467986	2	Passeriformes	Pycnonotidae	*Pycnonotus*	*tephrolaemus*	*fusciceps*	Southern Mountain Greenbul	*Leucocytozoon* sp.	L_AFR195	KM056513
467990	2	Passeriformes	Pycnonotidae	*Pycnonotus*	*tephrolaemus*	*fusciceps*	Southern Mountain Greenbul	*Leucocytozoon* sp.	L_AFR200	KM056518
467976	2	Passeriformes	Pycnonotidae	*Pycnonotus*	*tephrolaemus*	*fusciceps*	Southern Mountain Greenbul	*Leucocytozoon* sp.	L_AFR212	KM056530
467978	2	Passeriformes	Pycnonotidae	*Pycnonotus*	*tephrolaemus*	*fusciceps*	Southern Mountain Greenbul	*Leucocytozoon* sp.	L_AFR212	KM056530
467985	2	Passeriformes	Pycnonotidae	*Pycnonotus*	*tephrolaemus*	*fusciceps*	Southern Mountain Greenbul	*Leucocytozoon* sp.	L_AFR215	KM056532
467967	2	Passeriformes	Pycnonotidae	*Pycnonotus*	*tephrolaemus*	*fusciceps*	Southern Mountain Greenbul	*Leucocytozoon* sp.	L_AFR223	KM056537
467970	2	Passeriformes	Pycnonotidae	*Pycnonotus*	*tephrolaemus*	*fusciceps*	Southern Mountain Greenbul	*Leucocytozoon* sp.	L_AFR223	KM056537
467981	2	Passeriformes	Pycnonotidae	*Pycnonotus*	*tephrolaemus*	*fusciceps*	Southern Mountain Greenbul	*Leucocytozoon* sp.	L_AFR231	KM056545
467987	2	Passeriformes	Pycnonotidae	*Pycnonotus*	*tephrolaemus*	*fusciceps*	Southern Mountain Greenbul	*Leucocytozoon* sp.	L_AFR231	KM056545
467971	2	Passeriformes	Pycnonotidae	*Pycnonotus*	*tephrolaemus*	*fusciceps*	Southern Mountain Greenbul	*Leucocytozoon* sp.	L_AFR236	KM056550
467974	2	Passeriformes	Pycnonotidae	*Pycnonotus*	*tephrolaemus*	*fusciceps*	Southern Mountain Greenbul	*Leucocytozoon* sp.	L_AFR236	KM056550
467988	2	Passeriformes	Pycnonotidae	*Pycnonotus*	*tephrolaemus*	*fusciceps*	Southern Mountain Greenbul	*Leucocytozoon* sp.	L_AFR236	KM056550
467989	2	Passeriformes	Pycnonotidae	*Pycnonotus*	*tephrolaemus*	*fusciceps*	Southern Mountain Greenbul	*Leucocytozoon* sp.	L_AFR236	KM056550
467981	2	Passeriformes	Pycnonotidae	*Pycnonotus*	*tephrolaemus*	*fusciceps*	Southern Mountain Greenbul	*Leucocytozoon* sp.	L_AFR239	KM056553
467972	2	Passeriformes	Pycnonotidae	*Pycnonotus*	*tephrolaemus*	*fusciceps*	Southern Mountain Greenbul	0	NA	NA
467975	2	Passeriformes	Pycnonotidae	*Pycnonotus*	*tephrolaemus*	*fusciceps*	Southern Mountain Greenbul	0	NA	NA
467986	2	Passeriformes	Pycnonotidae	*Pycnonotus*	*tephrolaemus*	*fusciceps*	Southern Mountain Greenbul	*Plasmodium* sp.	P_AFR65	KM056607
467990	2	Passeriformes	Pycnonotidae	*Pycnonotus*	*tephrolaemus*	*fusciceps*	Southern Mountain Greenbul	*Plasmodium* sp.	P_BUL07	KM056642
467967	2	Passeriformes	Pycnonotidae	*Pycnonotus*	*tephrolaemus*	*fusciceps*	Southern Mountain Greenbul	*Plasmodium* sp.	P_COLL7	KM056625
468316	1	Passeriformes	Remizidae	*Anthoscopus*	*caroli*	*robertsi*	African Penduline-Tit	0	NA	NA
468291	2	Passeriformes	Stenostiridae	*Elminia*	*albonotata*		White-tailed Crested-Flycatcher	0	NA	NA

468602	1	Passeriformes	Sturnidae	*Lamprotornis*	*chalybaeus*	*sycobius*	Greater Blue-eared Starling	Unknown	Coinfection	NA
468601	1	Passeriformes	Sturnidae	*Lamprotornis*	*chalybaeus*	*sycobius*	Greater Blue-eared Starling	*Parahaemoproteus* sp.	H_ZOSMAD01	KM056404
468601	1	Passeriformes	Sturnidae	*Lamprotornis*	*chalybaeus*	*sycobius*	Greater Blue-eared Starling	*Leucocytozoon* sp.	L_AFR211	KM056529
468601	1	Passeriformes	Sturnidae	*Lamprotornis*	*chalybaeus*	*sycobius*	Greater Blue-eared Starling	*Plasmodium* sp.	P_BUL07	KM056642
468603	1	Passeriformes	Sturnidae	*Lamprotornis*	*chloropterus*	*elisabeth*	Lesser Blue-eared Glossy-Starling	*Parahaemoproteus* sp.	H_AFR41	KM056448
468607	1	Passeriformes	Sturnidae	*Lamprotornis*	*chloropterus*	*elisabeth*	Lesser Blue-eared Glossy-Starling	*Parahaemoproteus* sp.	H_AFR76	KM056466
468605	1	Passeriformes	Sturnidae	*Lamprotornis*	*chloropterus*	*elisabeth*	Lesser Blue-eared Glossy-Starling	0	NA	NA
468608	1	Passeriformes	Sturnidae	*Lamprotornis*	*chloropterus*	*elisabeth*	Lesser Blue-eared Glossy-Starling	0	NA	NA
468590	1	Passeriformes	Sturnidae	*Neocichla*	*gutturalis*	*angusta*	Babbling Starling	*Parahaemoproteus* sp.	H_AFR8	KM056468
468595	1	Passeriformes	Sturnidae	*Neocichla*	*gutturalis*	*angusta*	Babbling Starling	*Parahaemoproteus* sp.	H_AFR81	KM056469
468600	1	Passeriformes	Sturnidae	*Neocichla*	*gutturalis*	*angusta*	Babbling Starling	*Parahaemoproteus* sp.	H_AFR84	KM056470
468594	1	Passeriformes	Sturnidae	*Neocichla*	*gutturalis*	*angusta*	Babbling Starling	*Leucocytozoon* sp.	L_AFR211	KM056529
468593	1	Passeriformes	Sturnidae	*Neocichla*	*gutturalis*	*angusta*	Babbling Starling	*Leucocytozoon* sp.	L_AFR228	KM056542
468594	1	Passeriformes	Sturnidae	*Neocichla*	*gutturalis*	*angusta*	Babbling Starling	*Plasmodium* sp.	P_ACCTAC01	KM056621
468593	1	Passeriformes	Sturnidae	*Neocichla*	*gutturalis*	*angusta*	Babbling Starling	*Plasmodium* sp.	P_AFR80	KM056610
468595	1	Passeriformes	Sturnidae	*Neocichla*	*gutturalis*	*angusta*	Babbling Starling	*Plasmodium* sp.	P_AFR82	KM056611
468589	2	Passeriformes	Sturnidae	*Onychognathus*	*tenuirostris*	*theresae*	Slender-billed Starling	*Parahaemoproteus* sp.	H_AFR137	KM056432
468589	2	Passeriformes	Sturnidae	*Onychognathus*	*tenuirostris*	*theresae*	Slender-billed Starling	*Leucocytozoon* sp.	L_AFR215	KM056532
468172	1	Passeriformes	Sylviidae	*Acrocephalus*	*cinnamomeus*	*cinnamomeus*	Cinnamon Bracken-Warbler	*Haemoproteus belopolskyi*	H_ARW1	KM056407
468171	1	Passeriformes	Sylviidae	*Acrocephalus*	*cinnamomeus*	*cinnamomeus*	Cinnamon Bracken-Warbler	*Haemoproteus belopolskyi*	H_MW1	KM056408
468170	1	Passeriformes	Sylviidae	*Acrocephalus*	*cinnamomeus*	*cinnamomeus*	Cinnamon Bracken-Warbler	*Haemoproteus payevski*	H_RW1	KM056406
468169	1	Passeriformes	Sylviidae	*Acrocephalus*	*cinnamomeus*	*cinnamomeus*	Cinnamon Bracken-Warbler	0	NA	NA
468173	1	Passeriformes	Sylviidae	*Acrocephalus*	*cinnamomeus*	*cinnamomeus*	Cinnamon Bracken-Warbler	0	NA	NA
468168	1	Passeriformes	Sylviidae	*Bradypterus*	*baboecala*	*tongensis*	African Bush-Warbler	*Parahaemoproteus* sp.	H_AFR85	KM056471
468162	1	Passeriformes	Sylviidae	*Bradypterus*	*baboecala*	*tongensis*	African Bush-Warbler	*Haemoproteus pallidus*	H_COLL2	KM056413
468164	1	Passeriformes	Sylviidae	*Bradypterus*	*baboecala*	*tongensis*	African Bush-Warbler	0	NA	NA
468162	1	Passeriformes	Sylviidae	*Bradypterus*	*baboecala*	*tongensis*	African Bush-Warbler	*Plasmodium* sp.	P_BUL07	KM056642
468168	1	Passeriformes	Sylviidae	*Bradypterus*	*baboecala*	*tongensis*	African Bush-Warbler	*Plasmodium* sp.	P_BUL07	KM056642
468167	1	Passeriformes	Sylviidae	*Bradypterus*	*baboecala*	*tongensis*	African Bush-Warbler	*Plasmodium* sp.	P_GRW09	KM056631
468150	2	Passeriformes	Sylviidae	*Bradypterus*	*cinnamomeus*	*nyassae*	Cinnamon Bracken-Warbler	Unknown	Coinfection	NA
468156	2	Passeriformes	Sylviidae	*Bradypterus*	*cinnamomeus*	*nyassae*	Cinnamon Bracken-Warbler	*Parahaemoproteus* sp.	H_AFR116	KM056424
468157	2	Passeriformes	Sylviidae	*Bradypterus*	*cinnamomeus*	*nyassae*	Cinnamon Bracken-Warbler	*Haemoproteus micronuclearis*	H_QUERY01	KM056416
468155	2	Passeriformes	Sylviidae	*Bradypterus*	*cinnamomeus*	*nyassae*	Cinnamon Bracken-Warbler	*Leucocytozoon* sp.	L_AFR176	KM056494
468149	2	Passeriformes	Sylviidae	*Bradypterus*	*cinnamomeus*	*nyassae*	Cinnamon Bracken-Warbler	0	NA	NA
468147	2	Passeriformes	Sylviidae	*Bradypterus*	*cinnamomeus*	*nyassae*	Cinnamon Bracken-Warbler	0	NA	NA
468153	2	Passeriformes	Sylviidae	*Bradypterus*	*cinnamomeus*	*nyassae*	Cinnamon Bracken-Warbler	0	NA	NA
468154	2	Passeriformes	Sylviidae	*Bradypterus*	*cinnamomeus*	*nyassae*	Cinnamon Bracken-Warbler	0	NA	NA
468155	2	Passeriformes	Sylviidae	*Bradypterus*	*cinnamomeus*	*nyassae*	Cinnamon Bracken-Warbler	*Plasmodium* sp.	P_AFR115	KM056571
468152	2	Passeriformes	Sylviidae	*Bradypterus*	*cinnamomeus*	*nyassae*	Cinnamon Bracken-Warbler	*Plasmodium* sp.	P_MALNI02	KM056641
468155	2	Passeriformes	Sylviidae	*Bradypterus*	*cinnamomeus*	*nyassae*	Cinnamon Bracken-Warbler	*Plasmodium* sp.	P_MALNI02	KM056641
468247	2	Passeriformes	Sylviidae	*Chloropeta*	*natalensis*	*massaica*	Dark-capped Yellow Warbler	*Leucocytozoon* sp.	L_AFR241	KM056554
468248	2	Passeriformes	Sylviidae	*Chloropeta*	*natalensis*	*massaica*	Dark-capped Yellow Warbler	0	NA	NA
468247	2	Passeriformes	Sylviidae	*Chloropeta*	*natalensis*	*massaica*	Dark-capped Yellow Warbler	*Plasmodium* sp.	P_AFR104	KM056565
468240	2	Passeriformes	Sylviidae	*Chloropeta*	*similis*		Mountain Yellow Warbler	*Leucocytozoon* sp.	L_RECOB3	KM056648
468239	2	Passeriformes	Sylviidae	*Chloropeta*	*similis*		Mountain Yellow Warbler	*Leucocytozoon* sp.	L_WW6	KM056645
468243	2	Passeriformes	Sylviidae	*Chloropeta*	*similis*		Mountain Yellow Warbler	0	NA	NA
468245	2	Passeriformes	Sylviidae	*Chloropeta*	*similis*		Mountain Yellow Warbler	0	NA	NA
468244	2	Passeriformes	Sylviidae	*Chloropeta*	*similis*		Mountain Yellow Warbler	*Plasmodium* sp.	P_SYBOR11	KM056638
468179	2	Passeriformes	Sylviidae	*Phylloscopus*	*ruficapilla*	*johnstoni*	Yellow-throated Woodland-Warbler	*Leucocytozoon* sp.	L_AFR198	KM056516
468179	2	Passeriformes	Sylviidae	*Phylloscopus*	*ruficapilla*	*johnstoni*	Yellow-throated Woodland-Warbler	*Leucocytozoon* sp.	L_AFR199	KM056517
468178	2	Passeriformes	Sylviidae	*Phylloscopus*	*ruficapilla*	*johnstoni*	Yellow-throated Woodland-Warbler	0	NA	NA
468177	2	Passeriformes	Sylviidae	*Phylloscopus*	*ruficapilla*	*johnstoni*	Yellow-throated Woodland-Warbler	*Plasmodium* sp.	P_BUL07	KM056642
468183	1	Passeriformes	Sylviidae	*Phylloscopus*	*trochilus*		Willow Warbler	*Parahaemoproteus* sp.	H_WW1	KM056414

468184	1	Passeriformes	Sylviidae	*Phylloscopus*	*trochilus*		Willow Warbler	0	NA	NA
468187	2	Passeriformes	Sylviidae	*Sylvia*	*atricapilla*	*atricapilla*	Eurasian Blackcap	0	NA	NA
468185	1	Passeriformes	Sylviidae	*Sylvia*	*borin*		Garden Warbler	Unknown	Coinfection	NA
468186	2	Passeriformes	Sylviidae	*Sylvia*	*borin*		Garden Warbler	*Haemoproteus parabelopolskyi*	H_SYBOR01	KM056410
468186	2	Passeriformes	Sylviidae	*Sylvia*	*borin*		Garden Warbler	*Leucocytozoon* sp.	L_AFR211	KM056529
468143	2	Passeriformes	Timaliidae	*Alcippe*	*abyssinica*	*stierlingi*	African Hill Babbler	*Leucocytozoon spp.*	Coinfection	*Leucocytozoon* spp.
468141	2	Passeriformes	Timaliidae	*Alcippe*	*abyssinica*	*stierlingi*	African Hill Babbler	*Leucocytozoon* sp.	L_AFR185	KM056503
468142	2	Passeriformes	Timaliidae	*Alcippe*	*abyssinica*	*stierlingi*	African Hill Babbler	*Leucocytozoon* sp.	L_AFR212	KM056530
468140	2	Passeriformes	Timaliidae	*Alcippe*	*abyssinica*	*stierlingi*	African Hill Babbler	*Leucocytozoon* sp.	L_AFR215	KM056532
468142	2	Passeriformes	Timaliidae	*Alcippe*	*abyssinica*	*stierlingi*	African Hill Babbler	*Leucocytozoon* sp.	L_AFR215	KM056532
468139	2	Passeriformes	Timaliidae	*Alcippe*	*abyssinica*	*stierlingi*	African Hill Babbler	*Leucocytozoon* sp.	L_AFR243	KM056556
468140	2	Passeriformes	Timaliidae	*Alcippe*	*abyssinica*	*stierlingi*	African Hill Babbler	*Plasmodium* sp.	P_BUL07	KM056642
468144	2	Passeriformes	Timaliidae	*Trichastoma*	*pyrrhopterum*		Mountain Illadopsis	Unknown	Coinfection	NA
468144	2	Passeriformes	Timaliidae	*Trichastoma*	*pyrrhopterum*		Mountain Illadopsis	*Parahaemoproteus* sp.	H_AFR71	KM056462
468072	2	Passeriformes	Turdidae	*Alethe*	*fuelleborni*	*fuelleborni*	White-chested Alethe (nominate)	*Parahaemoproteus* sp.	H_AFR103	KM056420
468075	2	Passeriformes	Turdidae	*Alethe*	*fuelleborni*	*fuelleborni*	White-chested Alethe (nominate)	*Leucocytozoon* sp.	L_AFR179	KM056497
468074	2	Passeriformes	Turdidae	*Alethe*	*fuelleborni*	*fuelleborni*	White-chested Alethe (nominate)	*Leucocytozoon* sp.	L_AFR220	KM056535
468075	2	Passeriformes	Turdidae	*Alethe*	*fuelleborni*	*fuelleborni*	White-chested Alethe (nominate)	*Leucocytozoon* sp.	L_AFR220	KM056535
468073	2	Passeriformes	Turdidae	*Alethe*	*fuelleborni*	*fuelleborni*	White-chested Alethe (nominate)	*Leucocytozoon* sp.	L_AFR226	KM056540
468073	2	Passeriformes	Turdidae	*Alethe*	*fuelleborni*	*fuelleborni*	White-chested Alethe (nominate)	*Leucocytozoon* sp.	L_AFR232	KM056546
468071	2	Passeriformes	Turdidae	*Alethe*	*fuelleborni*	*fuelleborni*	White-chested Alethe (nominate)	0	NA	NA
468075	2	Passeriformes	Turdidae	*Alethe*	*fuelleborni*	*fuelleborni*	White-chested Alethe (nominate)	*Plasmodium* sp.	P_PSEGRI01	KM056637
468127	2	Passeriformes	Turdidae	*Turdus*	*abyssinicus*	*nyikae*	Olive Thrush	*Parahaemoproteus* sp.	H_AFR103	KM056420
468127	2	Passeriformes	Turdidae	*Turdus*	*abyssinicus*	*nyikae*	Olive Thrush	*Leucocytozoon* sp.	L_AFR236	KM056550
468130	2	Passeriformes	Turdidae	*Turdus*	*abyssinicus*	*nyikae*	Olive Thrush	*Leucocytozoon* sp.	L_AFR248	KM056561
468128	2	Passeriformes	Turdidae	*Turdus*	*abyssinicus*	*nyikae*	Olive Thrush	*Plasmodium* sp.	P_RFF1	KM056632
468122	1	Passeriformes	Turdidae	*Turdus*	*libonyanus*	*tropicalis*	Kurrichane Thrush	0	NA	NA
468123	1	Passeriformes	Turdidae	*Turdus*	*libonyanus*	*tropicalis*	Kurrichane Thrush	*Plasmodium* sp.	P_AFTRU4	KM056635
468126	1	Passeriformes	Turdidae	*Turdus*	*libonyanus*	*tropicalis*	Kurrichane Thrush	*Plasmodium* sp.	P_AFTRU4	KM056635
468118	2	Passeriformes	Turdidae	*Zoothera*	*gurneyi*	*otomitra*	Orange Ground-Thrush	*Leucocytozoon spp.*	Coinfection	*Leucocytozoon* spp.
468119	2	Passeriformes	Turdidae	*Zoothera*	*gurneyi*	*otomitra*	Orange Ground-Thrush	*Parahaemoproteus* sp.	H_AFR130	KM056430
468119	2	Passeriformes	Turdidae	*Zoothera*	*gurneyi*	*otomitra*	Orange Ground-Thrush	*Leucocytozoon* sp.	L_AFR187	KM056505
468121	2	Passeriformes	Turdidae	*Zoothera*	*gurneyi*	*otomitra*	Orange Ground-Thrush	*Leucocytozoon* sp.	L_AFR201	KM056519
468120	2	Passeriformes	Turdidae	*Zoothera*	*gurneyi*	*otomitra*	Orange Ground-Thrush	0	NA	NA
468121	2	Passeriformes	Turdidae	*Zoothera*	*gurneyi*	*otomitra*	Orange Ground-Thrush	*Plasmodium elongatum*	P_GRW06	KM056633
468484	1	Passeriformes	Viduidae	*Vidua*	*macroura*		Pin-tailed Whydah	0	NA	NA
468398	2	Passeriformes	Zosteropidae	*Zosterops*	*senegalensis*	*stierlingi*	Yellow White-eye	Unknown	Coinfection	NA
468394	2	Passeriformes	Zosteropidae	*Zosterops*	*senegalensis*	*stierlingi*	Yellow White-eye	*Parahaemoproteus* sp.	H_AFR96	KM056473
468395	2	Passeriformes	Zosteropidae	*Zosterops*	*senegalensis*	*stierlingi*	Yellow White-eye	*Parahaemoproteus* sp.	H_AFR96	KM056473
468399	2	Passeriformes	Zosteropidae	*Zosterops*	*senegalensis*	*stierlingi*	Yellow White-eye	*Parahaemoproteus* sp.	H_AFR96	KM056473
468400	2	Passeriformes	Zosteropidae	*Zosterops*	*senegalensis*	*stierlingi*	Yellow White-eye	*Parahaemoproteus* sp.	H_AFR96	KM056473
468396	2	Passeriformes	Zosteropidae	*Zosterops*	*senegalensis*	*stierlingi*	Yellow White-eye	*Parahaemoproteus* sp.	H_YEWE2	KM056405
468401	2	Passeriformes	Zosteropidae	*Zosterops*	*senegalensis*	*stierlingi*	Yellow White-eye	*Parahaemoproteus* sp.	H_YEWE2	KM056405
468402	2	Passeriformes	Zosteropidae	*Zosterops*	*senegalensis*	*stierlingi*	Yellow White-eye	*Parahaemoproteus* sp.	H_YEWE2	KM056405
468398	2	Passeriformes	Zosteropidae	*Zosterops*	*senegalensis*	*stierlingi*	Yellow White-eye	*Parahaemoproteus* sp.	H_ZOSMAD01	KM056404
468393	2	Passeriformes	Zosteropidae	*Zosterops*	*senegalensis*	*stierlingi*	Yellow White-eye	*Parahaemoproteus* sp.	H_ZOSMAD01	KM056404
468397	2	Passeriformes	Zosteropidae	*Zosterops*	*senegalensis*	*stierlingi*	Yellow White-eye	*Parahaemoproteus* sp.	H_ZOSMAD01	KM056404
468405	2	Passeriformes	Zosteropidae	*Zosterops*	*senegalensis*	*stierlingi*	Yellow White-eye	*Parahaemoproteus* sp.	H_ZOSMAD01	KM056404
468406	2	Passeriformes	Zosteropidae	*Zosterops*	*senegalensis*	*stierlingi*	Yellow White-eye	*Parahaemoproteus* sp.	H_ZOSMAD01	KM056404
468409	2	Passeriformes	Zosteropidae	*Zosterops*	*senegalensis*	*stierlingi*	Yellow White-eye	*Parahaemoproteus* sp.	H_ZOSMAD01	KM056404
468408	2	Passeriformes	Zosteropidae	*Zosterops*	*senegalensis*	*stierlingi*	Yellow White-eye	*Leucocytozoon* sp.	L_AFR197	KM056515
468395	2	Passeriformes	Zosteropidae	*Zosterops*	*senegalensis*	*stierlingi*	Yellow White-eye	*Leucocytozoon* sp.	L_AFR211	KM056529
468397	2	Passeriformes	Zosteropidae	*Zosterops*	*senegalensis*	*stierlingi*	Yellow White-eye	*Leucocytozoon* sp.	L_AFR211	KM056529

468399	2	Passeriformes	Zosteropidae	*Zosterops*	*senegalensis*	*stierlingi*	Yellow White-eye	*Leucocytozoon* sp.	L_AFR211	KM056529
468400	2	Passeriformes	Zosteropidae	*Zosterops*	*senegalensis*	*stierlingi*	Yellow White-eye	*Leucocytozoon* sp.	L_AFR211	KM056529
468401	2	Passeriformes	Zosteropidae	*Zosterops*	*senegalensis*	*stierlingi*	Yellow White-eye	*Leucocytozoon* sp.	L_AFR211	KM056529
468402	2	Passeriformes	Zosteropidae	*Zosterops*	*senegalensis*	*stierlingi*	Yellow White-eye	*Leucocytozoon* sp.	L_AFR211	KM056529
468405	2	Passeriformes	Zosteropidae	*Zosterops*	*senegalensis*	*stierlingi*	Yellow White-eye	*Leucocytozoon* sp.	L_AFR212	KM056530
468406	2	Passeriformes	Zosteropidae	*Zosterops*	*senegalensis*	*stierlingi*	Yellow White-eye	*Leucocytozoon* sp.	L_AFR212	KM056530
468409	2	Passeriformes	Zosteropidae	*Zosterops*	*senegalensis*	*stierlingi*	Yellow White-eye	*Leucocytozoon* sp.	L_AFR212	KM056530
468396	2	Passeriformes	Zosteropidae	*Zosterops*	*senegalensis*	*stierlingi*	Yellow White-eye	*Plasmodium* sp.	P_AFR97	KM056619
468398	2	Passeriformes	Zosteropidae	*Zosterops*	*senegalensis*	*stierlingi*	Yellow White-eye	*Plasmodium* sp.	P_BUL07	KM056642
468402	2	Passeriformes	Zosteropidae	*Zosterops*	*senegalensis*	*stierlingi*	Yellow White-eye	*Plasmodium* sp.	P_MALNI02	KM056641
467924	1	Piciformes	Indicatoridae	*Indicator*	*indicator*		Greater Honeyguide	0	NA	NA
467926	1	Piciformes	Indicatoridae	*Indicator*	*indicator*		Greater Honeyguide	*Plasmodium* sp.	P_AFR54	KM056602
467926	1	Piciformes	Indicatoridae	*Indicator*	*indicator*		Greater Honeyguide	*Plasmodium* sp.	P_AFR55	KM056603
467922	1	Piciformes	Indicatoridae	*Indicator*	*minor*	*teitensis*	Lesser Honeyguide	0	NA	NA
467928	2	Piciformes	Indicatoridae	*Indicator*	*variegatus*	*variegatus*	Scaly-throated Honeyguide	0	NA	NA
467927	2	Piciformes	Indicatoridae	*Indicator*	*variegatus*	*variegatus*	Scaly-throated Honeyguide	*Plasmodium* sp.	P_BUL07	KM056642
467939	1	Piciformes	Picidae	*Campethera*	*abingoni*	*suahelica*	Golden-tailed Woodpecker	*Parahaemoproteus* sp.	H_AFR71	KM056462
467939	1	Piciformes	Picidae	*Campethera*	*abingoni*	*suahelica*	Golden-tailed Woodpecker	*Leucocytozoon* sp.	L_AFR228	KM056542
467942	1	Piciformes	Picidae	*Dendropicos*	*fuscescens*	*camacupae*	Cardinal Woodpecker	0	NA	NA
467941	1	Piciformes	Picidae	*Dendropicos*	*fuscescens*	*camacupae*	Cardinal Woodpecker	*Plasmodium* sp.	P_PSEGRI01	KM056637
467934	1	Piciformes	Ramphastidae	*Lybius*	*torquatus*	*pumilio*	Black-collared Barbet	*Parahaemoproteus* sp.	H_AFR71	KM056462
467933	1	Piciformes	Ramphastidae	*Lybius*	*torquatus*	*pumilio*	Black-collared Barbet	0	NA	NA
467932	2	Piciformes	Ramphastidae	*Pogoniulus*	*leucomystax*	*leucomystax*	Moustached Green-Tinkerbird	Unknown	Coinfection	NA
467931	2	Piciformes	Ramphastidae	*Pogoniulus*	*leucomystax*	*leucomystax*	Moustached Green-Tinkerbird	*Parahaemoproteus* sp.	H_AFR133	KM056431
467930	2	Piciformes	Ramphastidae	*Pogoniulus*	*leucomystax*	*leucomystax*	Moustached Green-Tinkerbird	0	NA	NA
467935	1	Piciformes	Ramphastidae	*Trachyphonus*	*vaillantii*	*suahelicus*	Crested Barbet	*Parahaemoproteus* sp.	H_AFR2	KM056443
467872	1	Psittaciformes	Psittacidae	*Poicephalus*	*meyeri*	*matschiei*	Meyer's Parrot	*Plasmodium* sp.	P_MALNI02	KM056641
467897	2	Trogoniformes	Trogonidae	*Heterotrogon*	*vittatus*	*vittatus*	Bar-tailed Trogon	Unknown	Coinfection	NA
467897	2	Trogoniformes	Trogonidae	*Heterotrogon*	*vittatus*	*vittatus*	Bar-tailed Trogon	*Parahaemoproteus* sp.	H_AFR138	KM056433
467896	2	Trogoniformes	Trogonidae	*Heterotrogon*	*vittatus*	*vittatus*	Bar-tailed Trogon	*Leucocytozoon* sp.	L_AFR202	KM056520
467896	2	Trogoniformes	Trogonidae	*Heterotrogon*	*vittatus*	*vittatus*	Bar-tailed Trogon	*Plasmodium* sp.	P_BUL07	KM056642

* Site 1: Malawi; Rumphi District; Vwaza Wildlife Reserve, 11˚ 08.033’ S, 33˚ 39.307’ E, 1071 – 1170 m
Site 2: Malawi; Rumphi District; Nyika National Park, 10˚ 35.307’ S, 33˚ 48.670’ E, 1647 – 2347 m
NA = Collected between sites 1 and 2
** 0 = Uninfected; no infection detected after five PCR screens
