# Supplementary material for: Parasite Prevalence Corresponds to Host Life History in a Diverse Assemblage of Afrotropical Birds and Haemosporidian Parasites
Source: PLoS One. 2015 Apr 8;10(4):e0121254. doi: 10.1371/journal.pone.0121254 (PMC4390322; doi:10.1371/journal.pone.0121254)
Supplement: S3 Table — (PDF) [file pone.0121254.s003.pdf]

Table S3. Host species and life history traits

Order	Family	Genus	Species	Subspecies	Common Name	Nest Type	Nest Location	Flocking Behavior	Habitat
Anseriformes	Anatidae	*Alopochen*	*aegyptiaca*		Egyptian Goose	1	1	2	4
Anseriformes	Anatidae	*Sarkidiornis*	*melanotos*	*melanotos*	Knob-billed Duck	3	3	2	4
Bucerotiformes	Bucerotidae	*Tockus*	*alboterminatus*	*suahelicus*	Crowned Hornbill	3	3	1	1
Bucerotiformes	Bucerotidae	*Tockus*	*nasutus*	*epirhinus*	African Gray Hornbill	3	3	1	3
Caprimulgiformes	Caprimulgidae	*Caprimulgus*	*pectoralis*	*fervidus*	Fiery-necked Nightjar	1	1	1	3
Caprimulgiformes	Caprimulgidae	*Caprimulgus*	*ruwenzorii*	*guttifer*	Montane Nightjar	1	1	1	3
Caprimulgiformes	Caprimulgidae	*Caprimulgus*	*fossii*	*welwitschii*	Square-tailed Nightjar	1	1	1	2
Ciconiiformes	Ardeidae	*Ardea*	*melanocephala*		Black-headed Heron	1	3	1	4
Ciconiiformes	Ardeidae	*Bubulcus*	*ibis*		Cattle Egret	1	3	2	2
Ciconiiformes	Scopidae	*Scopus*	*umbretta*		Hamerkop	1	3	1	4
Coliiformes	Coliidae	*Colius*	*striatus*	*berlepschi*	Speckled Mousebird	1	2	2	3
Columbiformes	Columbidae	*Columba*	*arquatrix*		Rameron Pigeon	1	3	2	3
Columbiformes	Columbidae	*Streptopelia*	*capicola*	*tropica*	Cape Turtle-Dove	1	3	1	2
Columbiformes	Columbidae	*Streptopelia*	*semitorquata*	*semitorquata*	Red-eyed Dove	1	3	1	3
Columbiformes	Columbidae	*Treron*	*calva*	*schalowi*	African Green-Pigeon	1	3	2	5
Columbiformes	Columbidae	*Turtur*	*chalcospilos*	*chalcospilos*	Emerald-spotted Wood-Dove	1	2	1	1
Coraciiformes	Alcedinidae	*Alcedo*	*cristata*	*cristata*	Malachite Kingfisher	3	1	1	4
Coraciiformes	Alcedinidae	*Halcyon*	*senegalensis*	*cyanoleuca*	Woodland Kingfisher	3	3	1	3
Coraciiformes	Alcedinidae	*Ispidina*	*picta*	*natalensis*	African Pygmy-Kingfisher	3	1	1	1
Coraciiformes	Coraciidae	*Eurystomus*	*glaucurus*	*glaucurus*	Broad-billed Roller	3	3	1	3
Coraciiformes	Meropidae	*Merops*	*apiaster*		European Bee-eater	3	1	2	2
Coraciiformes	Meropidae	*Merops*	*pusillus*	*meridionalis*	Little Bee-eater	3	1	2	2
Cuculiformes	Cuculidae	*Centropus*	*superciliosus*	*loandae*	White-browed Coucal	1	2	1	3
Cuculiformes	Cuculidae	*Chrysococcyx*	*klaas*	*klaas*	Klaas's Cuckoo	4	5	1	3
Cuculiformes	Cuculidae	*Cuculus*	*canorus*	*gularis*	African Cuckoo	4	3	1	1
Falconiformes	Accipitridae	*Aquila*	*wahlbergi*		Wahlberg's Eagle	1	3	1	3
Falconiformes	Accipitridae	*Milvus*	*migrans*	*parasitus*	Black (Yellow-billed) Kite	1	3	1	3
Galliformes	Numididae	*Numida*	*meleagris*	*mitrata*	Helmeted Guineafowl	1	1	2	3
Galliformes	Phasianidae	*Coturnix*	*coturnix*	*africana*	Common Quail	1	1	1	2
Galliformes	Phasianidae	*Francolinus*	*levaillantii*	*crawshayi*	Red-winged Francolin	1	1	1	2
Galliformes	Phasianidae	*Gallus*	*gallus*		Chicken	0	0	0	0
Gruiformes	Rallidae	*Sarothrura*	*rufa*	*rufa*	Red-chested Flufftail	1	1	1	2
Musophagiformes	Musophagidae	*Tauraco*	*corythaix*	*schalowi*	Schalow's Turaco	1	3	1	2
Passeriformes	Alaudidae	*Mirafra*	*rufocinnamomea*	*fischeri*	Flappet Lark	2	1	1	1
Passeriformes	Cisticolidae	*Apalis*	*thoracica*	*youngi*	Bar-throated Apalis	2	2	1	5
Passeriformes	Cisticolidae	*Calamonastes*	*undosus*	*stierlingi*	Miombo Wren-Warbler	2	2	1	1
Passeriformes	Cisticolidae	*Cisticola*	*brachyptera*	*isabellina*	Short-winged Cisticola	2	2	1	2
Passeriformes	Cisticolidae	*Cisticola*	*erythrops*	*nyasa*	Red-faced Cisticola	2	2	1	2
Passeriformes	Cisticolidae	*Cisticola*	*fulvicapilla*	*muelleri*	Piping Cisticola	2	2	1	1
Passeriformes	Cisticolidae	*Cisticola*	*natalensis*	*matengorum*	Croaking Cisticola	2	2	1	2
Passeriformes	Cisticolidae	*Cisticola*	*nigriloris*		Black-lored Cisticola	2	2	2	2
Passeriformes	Cisticolidae	*Cisticola*	*njombe**		Churring Cisticola	2	2	1	2
Passeriformes	Cisticolidae	*Cisticola*	*rufilata*	*ansorgei*	Grey Cisticola	2	2	1	1
Passeriformes	Cisticolidae	*Cisticola*	*woosnami*	*lufira*	Trilling Cisticola	2	2	1	3
Passeriformes	Cisticolidae	*Prinia*	*erythroptera*	*rhodoptera*	Red-winged Warbler	2	2	1	2
Passeriformes	Corvidae	*Corvus*	*albicollis*		White-necked Raven	1	4	1	2
Passeriformes	Dicruridae	*Dicrurus*	*adsimilis*	*adsimilis*	Fork-tailed Drongo	1	3	3	3
Passeriformes	Emberizidae	*Emberiza*	*cabanisi*	*orientalis*	Cabanis's Bunting	1	3	1	1
Passeriformes	Emberizidae	*Emberiza*	*flaviventris*	*kalaharica*	Golden-brested Bunting	1	2	1	1
Passeriformes	Estrildidae	*Sporaeginthus*	*subflavus*	*clarkei*	Zebra Waxbill	2	2	2	2
Passeriformes	Estrildidae	*Cryptospiza*	*reichenovii*	*australis*	Red-faced Crimsonwing	2	2	1	5
Passeriformes	Estrildidae	*Estrilda*	*astrild*	*cavendishi*	Common Waxbill	2	1	2	2
Passeriformes	Estrildidae	*Estrilda*	*melanotis*	*stuartirwini*	Yellow-bellied Waxbill	2	2	2	3
Passeriformes	Estrildidae	*Hypargos*	*niveoguttatus*	*macrospilotus*	Peters's Twinspot	2	2	1	3
Passeriformes	Estrildidae	*Lagonosticta*	*rubricata*	*haematocephala*	African Firefinch	2	2	1	3
Passeriformes	Estrildidae	*Lonchura*	*cucullata*	*scutata*	Bronze Mannikin	2	2	2	2
Passeriformes	Estrildidae	*Pytilia*	*afra*		Orange-winged Pytilia	2	2	1	3
Passeriformes	Estrildidae	*Pytilia*	*melba*	*melba*	Green-winged Pytilia	2	2	1	3
Passeriformes	Estrildidae	*Uraeginthus*	*angolensis*	*niassensis*	Southern Cordonbleu	2	2	2	3
Passeriformes	Eurylaimidae	*Smithornis*	*capensis*	*albigularis*	African Broadbill	2	2	1	5
Passeriformes	Fringilidae	*Serinus*	*flavivertex*	*sassii*	Yellow-crowned Canary	1	2	2	2
Passeriformes	Fringilidae	*Serinus*	*hypostictus**	*hypostictus*	Southern Citril	1	2	2	3
Passeriformes	Fringillidae	*Serinus*	*mozambicus*	*mozambicus*	Yellow-fronted Canary	1	3	3	3
Passeriformes	Fringillidae	*Serinus*	*striolatus**	*whytii*	Yellow-browed Seedeater	1	2	1	3
Passeriformes	Hirundinidae	*Delichon*	*urbica*	*urbica*	House Martin	1	4	3	2
Passeriformes	Hirundinidae	*Hirundo*	*angolensis*	*angolensis*	Angola Swallow	1	4	2	2
Passeriformes	Hirundinidae	*Psalidoprocne*	*albiceps*	*albiceps*	White-headed Sawwing	3	4	1	3
Passeriformes	Laniidae	*Lanius*	*collaris*	*capelli*	Common Fiscal	1	5	1	2
Passeriformes	Malaconotidae	*Laniarius*	*ferrugineus*	*mossambicus*	Tropical Boubou	1	5	1	3
Passeriformes	Malaconotidae	*Laniarius*	*fulleborni**	*fulleborni*	Fuelleborn's Boubou	1	2	1	5
Passeriformes	Malaconotidae	*Malaconotus*	*blanchoti*	*hypopyrrhus*	Grey-headed Bush-Shrike	1	3	1	1
Passeriformes	Malaconotidae	*Tchagra*	*australis*	*congener*	Brown-crowned Tchagra	1	2	3	3
Passeriformes	Malaconotidae	*Tchagra*	*minutus*	*anchietae*	Marsh Tchagra	1	2	1	2
Passeriformes	Malaconotidae	*Tchagra*	*senegalus*	*armenus*	Black-crowned Tchagra	1	2	1	1
Passeriformes	Monarchidae	*Terpsiphone*	*viridis*	*plumbeiceps*	African Paradise-flycatcher	1	3	3	1
Passeriformes	Monarchidae	*Trochocercus*	*albonotatus*	*albonotatus*	White-tailed Crested-Flycatcher	1	2	3	5
Passeriformes	Motacillidae	*Anthus*	*cinnamomeus*	*lichenya*	African Pipit	1	1	1	2
Passeriformes	Muscicapidae	*Cossypha*	*anomala*	*macclounii*	Olive-flanked Robin-Chat	1	2	1	5
Passeriformes	Muscicapidae	*Cossypha*	*caffra*	*iolaema*	Cape Robin-Chat	1	2	1	3
Passeriformes	Muscicapidae	*Cossypha*	*heuglini*	*heuglini*	White-browed Robin-Chat	1	2	1	3
Passeriformes	Muscicapidae	*Sheppardia*	*sharpei*	*sharpei*	Sharpe's Akalat	1	2	1	5

Passeriformes	Muscicapidae	*Erythropygia*	*barbata*		Miombo Scrub-Robin	1	2	1	1
Passeriformes	Muscicapidae	*Erythropygia*	*leucophrys*	*zambesiana*	Red-backed Scrub Robin	1	2	1	1
Passeriformes	Muscicapidae	*Pseudalethe*	*fuelleborni*		White-chested Alethe	1	2	1	5
Passeriformes	Muscicapidae	*Ficedula*	*albicollis*	*albicollis*	Collared Flycatcher	3	3	3	1
Passeriformes	Muscicapidae	*Muscicapa*	*adusta*	*subadusta*	African Dusky Flycatcher	1	3	1	3
Passeriformes	Muscicapidae	*Muscicapa*	*coerulescens*	*impavida*	Ashy Flycatcher	1	3	1	3
Passeriformes	Muscicapidae	*Myrmecocichla*	*arnotti*	*arnotti*	White-headed Black-Chat	1	3	3	1
Passeriformes	Muscicapidae	*Pogonocichla*	*stellata*	*orientalis*	White-starred Robin	2	1	1	5
Passeriformes	Muscicapidae	*Saxicola*	*torquata*	*promiscua*	Common Stonechat	1	1	1	2
Passeriformes	Nectariniidae	*Nectarinia*	*afra*	*whytei*	Montane Double-collared Sunbird	2	5	1	3
Passeriformes	Nectariniidae	*Nectarinia*	*amethystina*	*kirkii*	Amethyst Sunbird	2	3	3	3
Passeriformes	Nectariniidae	*Nectarinia*	*famosa*	*aeneigularis/ cuprec*	Malachite Sunbird	2	2	1	3
Passeriformes	Nectariniidae	*Nectarinia*	*kilimensis*	*arturi*	Bronze Sunbird	2	2	1	3
Passeriformes	Nectariniidae	*Nectarinia*	*mediocris*	*fuelleborni*	Eastern Double-collard Sunbird	2	2	3	5
Passeriformes	Nectariniidae	*Nectarinia*	*olivacea*	*alfredi*	Olive Sunbird	2	2	1	5
Passeriformes	Nectariniidae	*Nectarinia*	*senegalensis*	*gutturalis*	Scarlet-chested Sunbird	2	3	3	1
Passeriformes	Nectariniidae	*Nectarinia*	*venusta*	*falkensteini*	Variable Sunbird	2	2	1	3
Passeriformes	Oriolidae	*Oriolus*	*auratus*	*notatus*	African Golden-Oriole	1	3	3	1
Passeriformes	Paridae	*Melaniparus*	*griseiventris*		Miombo Tit	3	3	3	1
Passeriformes	Paridae	*Melaniparus*	*niger*	*niger*	Southern Black-Tit	3	3	3	1
Passeriformes	Paridae	*Parus*	*rufiventris*	*???*	Rufous-bellied Tit	3	3	3	1
Passeriformes	Passeridae	*Petronia*	*superciliaris*		Yellow-throated Petronia	3	3	3	1
Passeriformes	Passeridae	*Plocepasser*	*rufoscapulatus*		Chestnut-backed Sparrow-Weaver	2	3	2	1
Passeriformes	Pellorneidae	*Illadopsis*	*pyrrhoptera*	*nyasae*	Mountain Illadopsis	1	2	2	5
Passeriformes	Platysteiridae	*Batis*	*dimorpha*	*sola*	Malawi Batis	1	3	3	5
Passeriformes	Platysteiridae	*Batis*	*molitor*	*palliditergum*	Chinspot Batis	1	3	3	1
Passeriformes	Ploceidae	*Euplectes*	*albonotatus*	*albonotatus*	White-winged Widowbird	2	2	3	2
Passeriformes	Ploceidae	*Euplectes*	*ardens*	*ardens*	Red-collared Widowbird	2	2	3	2
Passeriformes	Ploceidae	*Euplectes*	*capensis*	*crassirostris*	Yellow Bishop	2	2	3	2
Passeriformes	Ploceidae	*Euplectes*	*psammocromius*		Buff-shouldered Widowbird	2	2	2	2
Passeriformes	Ploceidae	*Ploceus*	*baglafecht*	*nyikae*	Baglafecht Weaver	2	5	2	3
Passeriformes	Ploceidae	*Ploceus*	*ocularis*	*suahelicus*	Spectacled Weaver	2	3	3	3
Passeriformes	Ploceidae	*Ploceus*	*velatus*	*shelleyi*	Southern Masked-Weaver	2	3	3	3
Passeriformes	Ploceidae	*Ploceus*	*xanthops*		Holub's Golden-Weaver	2	2	3	2
Passeriformes	Ploceidae	*Quelea*	*quelea*	*lathami*	Red-billed Quelea	2	5	2	2
Passeriformes	Pycnonotidae	*Phyllastrephus*	*cerviniventris*		Grey-olive Greenbul	1	2	2	1
Passeriformes	Pycnonotidae	*Phyllastrephus*	*flavostriatus*	*alfredi*	Yellow-streaked Greenbul	1	2	3	5
Passeriformes	Pycnonotidae	*Pycnonotus*	*barbatus*	*layardi*	Dark-capped Bulbul	1	3	1	3
Passeriformes	Pycnonotidae	*Arizelocichla*	*milanjensis*	*olivaceiceps*	Stripe-cheeked Greenbul	1	3	3	5
Passeriformes	Pycnonotidae	*Arizelocichla*	*nigriceps*	*fusciceps*	Eastern Mountain-Greenbul	1	2	3	5
Passeriformes	Remizidae	*Anthoscopus*	*caroli*	*robertsi*	African Penduline-Tit	2	3	1	1
Passeriformes	Stenostiridae	*Elminia*	*albonotata*	*albonotata*	White-tailed Crested-Flycatcher	1	2	3	5
Passeriformes	Sturnidae	*Lamprotornis*	*chalybaeus*	*sycobius*	Greater Blue-eared Glossy-Starling	3	3	2	3
Passeriformes	Sturnidae	*Lamprotornis*	*chloropterus*	*elisabeth*	Lesser Blue-eared Glossy-Starling	3	3	2	1
Passeriformes	Sturnidae	*Neocichla*	*gutturalis*	*angusta*	Babbling Starling	3	0	2	1
Passeriformes	Sturnidae	*Onychognathus*	*tenuirostris*	*theresae*	Slender-billed Starling	3	3	2	3
Passeriformes	Sylviidae	*Acrocephalus*	*cinnamomeus*	*cinnamomeus*	Cinnamon Bracken-Warbler	1	2	1	2
Passeriformes	Sylviidae	*Bradypterus*	*baboecala*	*tongensis*	African Bush-Warbler	1	2	1	2
Passeriformes	Sylviidae	*Bradypterus*	*cinnamomeus*	*nyassae*	Cinnamon Bracken-Warbler	1	2	1	5
Passeriformes	Sylviidae	*Iduna*	*natalensis*	*massaica*	African Yellow-Warbler	1	2	1	2
Passeriformes	Sylviidae	*Iduna*	*similis*		Mountain Yellow Warbler	1	2	1	2
Passeriformes	Sylviidae	*Phylloscopus*	*ruficapilla*	*johnstoni*	Yellow-throated Woodland-Warbler	1	1	3	5
Passeriformes	Sylviidae	*Phylloscopus*	*trochilus*		Willow Warbler	2	1	3	1
Passeriformes	Sylviidae	*Sylvia*	*abyssinica*	*stierlingi*	African Hill Babbler	1	2	1	5
Passeriformes	Sylviidae	*Sylvia*	*atricapilla*	*atricapilla*	Blackcap	1	2	3	3
Passeriformes	Sylviidae	*Sylvia*	*borin*		Garden Warbler	1	2	1	3
Passeriformes	Pellorneidae	*Illadopsis*	*pyrrhoptera*	*nyasae*	Mountain Illadopsis	1	2	2	5
Passeriformes	Turdidae	*Turdus*	*abyssinicus*	*nyikae*	Olive Thrush	1	3	1	5
Passeriformes	Turdidae	*Turdus*	*libonyanus*	*tropicalis*	Kurrichane Thrush	1	3	1	1
Passeriformes	Turdidae	*Zoothera*	*gurneyi*	*otomitra*	Orange Ground-Thrush	1	2	1	5
Passeriformes	Viduidae	*Vidua*	*macroura*		Pin-tailed Whydah	5	2	2	2
Passeriformes	Zosteropidae	*Zosterops*	*senegalensis***	*stierlingi*	Yellow White-eye	1	3	2	5
Piciformes	Indicatoridae	*Indicator*	*indicator*		Greater Honeyguide	5	5	1	3
Piciformes	Indicatoridae	*Indicator*	*minor*	*teitensis*	Lesser Honeyguide	5	5	1	3
Piciformes	Indicatoridae	*Indicator*	*variegatus*	*variegatus*	Scaly-throated Honeyguide	5	5	1	5
Piciformes	Lybiidae	*Lybius*	*torquatus*	*pumilio*	Black-collared Barbet	3	3	1	3
Piciformes	Lybiidae	*Pogoniulus*	*leucomystax*		Moustached Tinkerbird	3	3	3	5
Piciformes	Lybiidae	*Trachyphonus*	*vaillantii*	*suahelicus*	Crested Barbet	3	3	3	3
Piciformes	Picidae	*Campethera*	*abingoni*	*suahelica*	Golden-tailed Woodpecker	3	2	3	1
Piciformes	Picidae	*Dendropicos*	*fuscescens*	*camacupae*	Cardinal Woodpecker	3	2	3	1
Psittaciformes	Psittacidae	*Poicephalus*	*meyeri*	*matschiei*	Meyer's Parrot	3	3	2	1
Trogoniformes	Trogonidae	*Aploderma*	*vittatum*	*vittatum*	Bar-tailed Trogon	3	3	1	5

Nest Type: 1 = Open cup, 2 = Closed cup, 3 = Cavity, 4 = Open cup nest parasite, 5 = Cavity nest parasite
Nest Location: 1 = Ground, 2 = Understory, 3 = Canopy/subcanopy, 4 = Cliff or bank, 5 = Variable
Social System: 1 = Solitary (or in pairs), 2 = Single-species flock or family group, 3 = Mixed-species flock
Habitat: 1 = Woodland/riparian woodland, 2 = Grassland/marsh, 3 = Forest edge, 4 = Aquatic, 5 = Evergreen forest

*nest type, location, and/or habits for *Cisticola njombe*, *Serinus citrenilloides*, *Serinus striolatus*, *Laniarius fuelleborni*, and *Euplectes psammocromius* are inferred from the habits of close relatives
***Zosterops senegalensis* occurs in multiple habitat types; ours were caught in evergreen forest, so are given a 5 for habitat
